# Meta-Analysis of Randomized Controlled Trials on Yoga, Psychosocial, and Mindfulness-Based Interventions for Cancer-Related Fatigue: What Intervention Characteristics Are Related to Higher Efficacy?

**DOI:** 10.3390/cancers14082016

**Published:** 2022-04-15

**Authors:** Alexander Haussmann, Martina E. Schmidt, Mona L. Illmann, Marleen Schröter, Thomas Hielscher, Holger Cramer, Imad Maatouk, Markus Horneber, Karen Steindorf

**Affiliations:** 1 Division of Physical Activity, Prevention, and Cancer, German Cancer Research Center (DKFZ) and National Center for Tumor Diseases (NCT), Im Neuenheimer Feld 581, 69120 Heidelberg, Germany; alexander.haussmann@nct-heidelberg.de (A.H.); m.schmidt@dkfz.de (M.E.S.); monaluisa.illmann@dkfz.de (M.L.I.); 2 Department of Internal and Integrative Medicine, Evang. Kliniken Essen-Mitte and Faculty of Medicine, University of Duisburg-Essen, Am Deimelsberg 34a, 45276 Essen, Germany; m.schroeter@kem-med.com (M.S.); h.cramer@kem-med.com (H.C.); 3 Division of Biostatistics, German Cancer Research Center (DKFZ), Im Neuenheimer Feld 581, 69120 Heidelberg, Germany; t.hielscher@dkfz.de; 4 Division of Medical Psychosomatics, University Hospital Würzburg, Oberdürrbacher Straße 6, 97080 Würzburg, Germany; maatouk_i@ukw.de; 5 Division of Pneumology, Klinikum Nürnberg, Paracelsus Medical University, Prof.-Ernst-Nathan-Str. 1, 90340 Nürnberg, Germany; markus.horneber@klinikum-nuernberg.de

**Keywords:** fatigue, cancer, psychosocial, mindfulness, yoga, quality of life, patient-reported outcomes

## Abstract

**Simple Summary:**

Many individuals with cancer suffer from persistent exhaustion due to cancer therapy, known as cancer-related fatigue (CRF). Yoga, psychosocial, and mindfulness-based interventions are recommended to reduce CRF. However, it is not clear yet how interventions need to be designed to maximize their efficacy. This meta-analysis aimed to identify intervention characteristics associated with greater reductions in CRF. A total of 70 interventions with 6387 participants were included in the analysis. Our results found a positive effect of yoga, psychosocial, and mindfulness-based interventions, while all invention types revealed large differences in intervention effects. In psychosocial interventions, using a group setting and working on cognition was related to higher efficacy. Regarding yoga and mindfulness-based interventions, no specific intervention characteristics emerged as more favorable than others. Overall, this meta-analysis suggests opportunities to optimize psychosocial interventions for CRF, whereas the design of yoga and mindfulness-based interventions seems to allow for variation.

**Abstract:**

Cancer-related fatigue (CRF) is a burdensome sequela of cancer treatments. Besides exercise, recommended therapies for CRF include yoga, psychosocial, and mindfulness-based interventions. However, interventions conducted vary widely, and not all show a significant effect. This meta-analysis aimed to explore intervention characteristics related to greater reductions in CRF. We included randomized controlled trials published before October 2021. Standardized mean differences were used to assess intervention efficacy for CRF and multimodel inference to explore intervention characteristics associated with higher efficacy. For the meta-analysis, we included 70 interventions (24 yoga interventions, 31 psychosocial interventions, and 15 mindfulness-based interventions) with 6387 participants. The results showed a significant effect of yoga, psychosocial, and mindfulness-based interventions on CRF but with high heterogeneity between studies. For yoga and mindfulness-based interventions, no particular intervention characteristic was identified to be advantageous for reducing CRF. Regarding psychosocial interventions, a group setting and work on cognition were related to higher intervention effects on CRF. The results of this meta-analysis suggest options to maximize the intervention effects of psychosocial interventions for CRF. The effects of yoga and mindfulness-based interventions for CRF appear to be independent of their design, although the limited number of studies points to the need for further research.

## 1. Introduction

Cancer-related fatigue (CRF) is a tremendous burden for individuals with cancer. It is characterized by a perceived physical, emotional, and/or cognitive tiredness or exhaustion [1] and affects the majority of cancer patients during cancer treatment [2,3,4,5,6,7]. Remarkably, CRF often persists after completion of treatment and still affects 17 to 38% of cancer survivors more than 5 years post-diagnosis [8,9,10]. Cancer patients perceive CRF as a substantial threat to their quality of life [11] and indicate it as their most distressing symptom [12].

For individuals suffering from CRF, several interventions are available that have been shown to help reduce CRF [13]. According to the 2021 National Comprehensive Cancer Network (NCCN) Clinical Practice Guidelines in Oncology, physical exercise, yoga, and psychosocial interventions including mindfulness-based interventions were ranked with the highest level of evidence [14]. These non-pharmacological interventions also had a greater effect on CRF than pharmacological treatments [1,15,16]. At the same time, the effects of yoga, psychosocial interventions, and mindfulness-based interventions were found to vary widely [15,17,18,19]. Thus, it does not seem to be warranted that such an intervention will indeed always exert a significant mitigating effect on fatigue. When designing a yoga, psychosocial, or mindfulness-based intervention for CRF, there is a broad variety of possible intervention characteristics in terms of mode and modalities, all of which can potentially influence the intervention’s effectiveness. However, the NCCN guidelines do not recommend a specific intervention design to maximize efficacy for CRF.

Regarding exercise interventions, the current state of research was recently reviewed by a roundtable of international experts who made specific recommendations for exercise training in CRF [20]. For psychosocial and yoga interventions, first insights were gained into which intervention characteristics were associated with efficacy for CRF [15,16,17,21]. These insights were mainly based on subgroup comparisons that only analyzed one intervention characteristic at a time, although non-pharmacological interventions are defined through an interplay of different characteristics that can hardly be considered separately. This reality can be better captured by the information-theoretic approach of multimodel inference which is an established approach in ecology [22], usable for meta-analysis [23], and increasingly gaining attention in health sciences [24,25,26]. Multimodel inference offers the possibility to examine the most predictive meta-regression models for an intervention effect and to estimate the importance of single variables aggregated across these models. The effect of intervention characteristics can thus be determined in terms of their impact on CRF while simultaneously considering all other relevant characteristics in one statistical procedure.

For both psychosocial and yoga interventions, a large variety of approaches exists, which differ in several characteristics. Psychosocial interventions that have been applied to reducing CRF mainly include two different approaches. On the one hand, they are based on cognitive-behavioral theory assuming that coping with a symptom, such as CRF, is determined at least partially by its accompanying cognition and beliefs [27,28]. Accordingly, cognitive-behavioral interventions employ techniques primarily aimed at changing thoughts and beliefs (e.g., cognitive restructuring) and/or fostering functional behavior (e.g., activity scheduling). Psychoeducational elements in the form of verbal or written advice are often applied to support behavioral or cognitive methods by increasing patient knowledge and awareness but may also be effective on their own [29]. On the other hand, approaches based on mindfulness are usually subsumed under psychosocial interventions as well [13,14], but they are conceptually distinct. Mindfulness is a concept, derived from the Buddhist tradition, that seeks to strengthen the awareness of the present moment [30]. This is achieved in particular through conscious awareness of inner feelings, using various forms of meditation [31]. In contrast to cognitive-behavioral approaches, the focus of mindfulness-based interventions lies in identifying, accepting, and releasing cognition and emotions rather than changing them. This process is often supported by the inclusion of mindful movement practice. Mindfulness-based interventions are usually grounded in mindfulness-based stress reduction (MBSR) [32] or mindfulness-based cognitive therapy (MBCT) [32], although adaptations are implemented for both approaches [33,34,35]. In the following, we use the terms *psychosocial intervention* for systematic approaches including behavioral, cognitive, and/or psycho-educative elements and *mindfulness-based intervention* for approaches that focus on mindful experiencing, including but not limited to MBSR and MBCT.

Yoga interventions for cancer survivors offer a wide variability in styles and physical demands, including different forms of Hatha yoga with a focus on postures (asanas) [36,37,38], and restorative yoga with a focus on relaxation [39], or a mixture of both. Although yoga interventions are usually performed with mindful observation of internal physical processes and sensations [40], there is a varying focus on mental demands [41]. This also applies to the physical effort required to perform yoga exercises. There are yoga traditions without physical components focusing on meditation and breathing techniques [42,43], while most yoga interventions for cancer survivors applied today involve mild to moderate physical effort [21,44,45].

Overall, although there is large heterogeneity in the implementation of yoga, psychosocial, and mindfulness-based interventions, there is no gold standard on intervention characteristics for each intervention type that promises the highest benefit. A comprehensive and simultaneous analysis of intervention characteristics of previous studies on their effect on CRF can contribute to the further development of optimized yoga, psychosocial, and mindfulness-based interventions for CRF.

Thus, this review and meta-analysis aim to (1) analyze the general efficacy of yoga, psychosocial, and mindfulness-based interventions for CRF, (2) consider the heterogeneity of intervention effects, and (3) examine various characteristics of each intervention type for their relevance to intervention efficacy.

## 2. Materials and Methods

This review and meta-analysis were conducted and reported in accordance with the PRISMA guidelines (http://www.prisma-statement.org; accessed on 4 October 2021) and registered a priori in PROSPERO under the number CRD42021286121.

### 2.1. Eligibility Criteria

Original papers written in English were considered eligible if they met all of the following inclusion criteria: (a) use of a randomized controlled trial design; (b) adult cancer patients with current or previous curative systemic or radiation therapy; (c) interventions applying a psychosocial approach (i.e., systematic approach including behavioral, cognitive, and/or psycho-educative elements), a mindfulness-based approach (i.e., including but not limited to MBSR and MBCT), or yoga (i.e., all yoga styles), with no mixing of intervention types (e.g., acceptance and commitment therapies; exception: yoga exercises as part of mindfulness-based interventions), not exclusively conducted via telephone or online-based; (d) all kinds of control groups apart from treatment controls; (e) fatigue measured as a metric variable. Studies that only enrolled patients with advanced cancer, or that focused on survivorship care, lifestyle changes, alleviating sleep problems, the use of alternative or complementary medicine, or the treatment of a psychiatric disorder were excluded. The assessment of whether a study was considered as meeting all inclusion and exclusion criteria was made independently by two reviewers (A.H. and M.L.I.). If only one of the reviewers selected a study as appropriate, it was discussed with a third reviewer (M.E.S.), and a consensus was reached.

Data from duplicate publications reporting from the same study were considered only once. In case of unclear or incomplete information, the authors were contacted by email, and one reminder was sent if no response was received. If studies investigated more than one intervention, each intervention group meeting the inclusion criteria was analyzed separately.

### 2.2. Search Strategy

Our literature search was conducted in two consecutive steps. First, we considered all original publications of studies included in already published systematic reviews. We systematically searched the electronic databases PubMed, CINAHL, and PsycInfo for reviews of randomized controlled trials that investigated psychosocial interventions or mindfulness-based interventions with CRF as an outcome. Regarding yoga interventions, our pool of eligible studies was based on the search of the German Clinical Guidelines (‘S3-Leitlinie’) on complementary medicine that covered the databases CINAHL, Cochrane, MEDLINE, Embase, and PsychInfo [46].

Second, we added more recent studies by conducting a systematic search for studies published after the search period of the identified reviews for each intervention type up to 19 October 2021, again using PubMed, CINAHL, and PsycInfo. Our search strategies were adapted to each intervention type (see Tables S1 for details). All reviews and databases were screened independently by two reviewers (A.H. and M.L.I.).

### 2.3. Review Strategy

Pre- and post-intervention means of the CRF scores, standard deviations or standard errors, and numbers of participants in the intervention and control groups were independently extracted from the publications by two reviewers (A.H. and M.L.I.) and cross-checked. Further, included studies were categorized based on characteristics with regard to study population (i.e., age, cancer site, cancer stage, treatment), intervention procedure (i.e., intervention duration, contact frequency, number of sessions, session length, intervention setting (group or individual), fatigue instrument), and intervention content. Intervention characteristics were selected in agreement with the co-authors who are known experts in the field of psychosocial interventions (I.M.) or yoga and mindfulness-based interventions (H.C.), respectively. The selection aimed to extract the most potentially relevant characteristics for intervention efficacy that are adequately documented in the literature while avoiding overparameterization in the statistical analyses. Nevertheless, the content categories were selected to be sufficiently precise in depicting intervention content. Categories depended on the intervention type and included (a) yoga interventions: yoga style, breathing techniques, mental practice (i.e., meditation or imagery), physical effort, the variety between sessions, and home practice; (b) psychosocial interventions: *CRF* education, work on cognition, work on behavior, work on emotions, social resources, relaxation, hypnosis, and homework; (c) mindfulness-based interventions: CRF education, meditation, work on cognition, yoga exercises, and home practice. The characteristic focus on *CRF* was used for analyses of all intervention types. Precise definitions of intervention characteristics can be found below the intervention description tables (see Tables S2).

Categories were coded dichotomously (mostly yes/no) independently by two reviewers (A.H. and M.L.I., with consultation for yoga interventions by M.S. and H.C. and by I.M. for psychosocial interventions). As far as possible, the evaluation was based on the information in the manuscript; however, if the information was lacking or ambiguous, the authors of the study were contacted (and reminded once if necessary). If there were still different assessments by the two reviewers, these were discussed and solved.

### 2.4. Risk of Bias Assessment

The risk of bias in each trial was examined using the criteria recommended by the Cochrane Collaboration [47]. Accordingly, two reviewers (A.H. and M.L.I.) independently carried out the scoring on random sequence generation, allocation concealment, blinding of participants and personnel, blinding of outcome assessment, incomplete outcome data, selective outcome reporting, and other bias. Each domain was judged as low risk (requirements adequately fulfilled), high risk (requirements not adequately fulfilled), or unclear (information insufficient for judgment). If differences in ratings emerged, they were evaluated by a third reviewer (M.E.S.).

### 2.5. Statistical Analysis

All data on intervention characteristics were prepared in Excel lists to be read and analyzed in the statistical programs mentioned below. Standardized mean differences (SMDs) with 95% confidence intervals (CIs) were calculated for each study as the difference of the mean changes from pre- to post-intervention for total CRF scores between the intervention and the control group divided by a pooled pretest standard deviation [48] using SAS version 9.4. We chose the post-measurement time point closest to the end of the intervention for the study participants or, if intervention periods varied individually, the time point at which the majority had completed the intervention. Multidimensional questionnaires were preferred when multiple fatigue instruments were reported in a study, and total/general scores were preferred when multiple scales of one questionnaire were reported. If necessary, original fatigue scores were inverted. Negative SMDs indicate a mitigating effect of the intervention group on CRF compared to the control group. In detail, values of −0.2 to −0.5 indicate a small reducing effect of the intervention group on CRF compared to the control group, with values of −0.5 to −0.8 a medium effect, and values less than −0.8 a large effect [49]. Positive values represent a deteriorating effect of the intervention group on CRF compared to the control group. Random-effects models were calculated using the Cochrane software RevMan 5.3.

For quantifying heterogeneity of intervention effects, prediction intervals were reported to provide a range of the true effect [50] using the software Comprehensive Meta-Analysis Prediction Intervals (www.Meta-Analysis.com/Prediction; accessed on 7 December 2021).

In order to explore which characteristics of interventions might be relevant for their effectiveness, model selection based on multimodel inference was applied. The procedure considers all possible combinations of variables (i.e., intervention characteristics) to examine which combination leads to the best data fit based on the Akaike information criterion corrected for small samples (AICc). Model averaging was used to calculate a regression coefficient for each intervention characteristic as a weighted average over all models in which they appear, using AICc-based model weights [51]. In order to prevent including redundant models and models giving likely spurious results, we limited model averaging to the models that were within 6 units of the AICc compared to the best model [52].

The selection of intervention characteristics for multimodel analyses was restrictive to avoid overparameterization. In this regard, different approaches were conducted according to categories of intervention characteristics: (a) characteristics related to the study population were analyzed in advance using subgroup comparisons with χ^2^-analysis and in the case of significant differences (α = 0.1), multimodel analyses were adjusted for these characteristics. Corresponding analyses revealed a significant difference only for *CRF as an inclusion criterion* in mindfulness-based interventions (see all subgroup comparisons in Supplementary Section S1); (b) characteristics related to intervention procedure that described their duration and timing (*intervention duration*, *frequency of sessions*, *number of sessions*, *session length*) were aggregated to a variable representing the *total intervention time* which was used as a fixed covariate in all models, i.e., not subject to variable selection. *Intervention setting* was further included; (c) regarding characteristics of intervention content, only variables that exhibited sufficient variance between included interventions were selected (i.e., at least 10% of the studies had to differ in category). In addition, correlations between the remaining variables were calculated, and in the case of Phi or point-biserial correlation coefficients exceeding 0.8, consideration was given to excluding a variable or combining variables. Furthermore, the variable *homework/home-based practice* was not included in analyses as the related data in the studies varied greatly in scope and adequacy.

Multimodel procedures were performed using the metafor and glmulti packages in R (version 4.1.2; http://CRAN.R-project.org; accessed on 15 December 2021) [53,54]. Unless otherwise indicated, statistical significance was set at α = 0.05.

### 2.6. Publication Bias

A potential risk of publication bias was assessed based on the symmetry of funnel plots using both visual analysis and Egger’s regression test [55] with adapted standard errors to reduce the risk of false-positive results [56]. Asymmetry in funnel plots can be considered as an indicator for small-study effects, which is due to the fact that small studies are more likely to show large effects than studies based on large sample sizes [57]. Small-study effects may indicate a publication bias.

### 2.7. Sensitivity Analyses

Sensitivity analyses were conducted to compare the effects based on all studies with the effects when (a) excluding studies with a high risk of selection or attrition bias, (b) excluding studies with *n* < 25 patients per intervention arm, and (c) excluding studies that were aimed exclusively at a study population with a specific physical or mental impairment. Additionally, the effects of interventions with different kinds of controls (i.e., standard care, wait-list control, attention control) were compared.

## 3. Results

### 3.1. Included Studies

Our systematic search yielded 18 reviews including psychosocial interventions [18,58,59,60,61,62,63,64,65], mindfulness-based interventions [17,33,35,66,67,68,69], or different intervention types [15,16]. The German guidelines regarding yoga interventions were based on three reviews [15,19,70] and 12 additional studies. Overall, these reviews included 242 studies. Our additional search of databases for recent studies generated 1055 records that were initially screened. Of these, 978 were excluded based on information in the title or abstract. Overall, 318 studies were screened for eligibility by reading the full article. Articles were excluded if they did not meet our inclusion criteria because of study design (*n* = 3), study population (*n* = 30), intervention content (*n* = 81), type of controls (*n* = 19), or fatigue values (*n* = 117).

**Figure 1:**
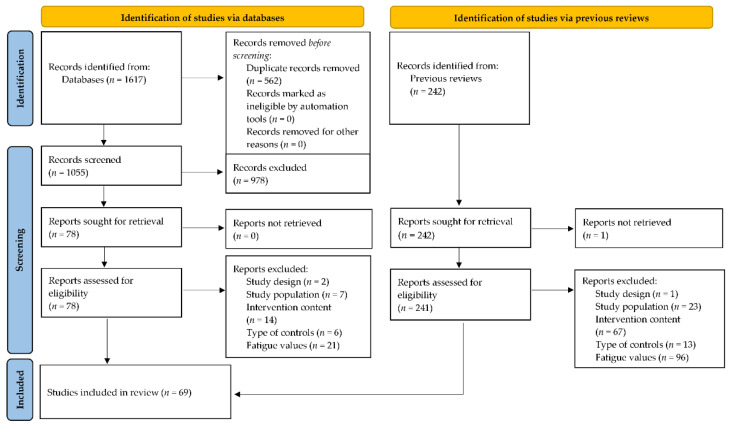
Flow chart of the study selection process [140].

The final study pool consisted of 69 studies, including 25 yoga studies [71,72,73,74,75,76,77,78,79,80,81,82,83,84,85,86,87,88,89,90,91,92,93,94,95], 29 psychosocial studies [96,97,98,99,100,101,102,103,104,105,106,107,108,109,110,111,112,113,114,115,116,117,118,119,120,121,122,123,124,125], and 15 mindfulness-based studies [118,126,127,128,129,130,131,132,133,134,135,136,137,138,139]. The PRISMA selection flow chart is presented in Figure 1.

### 3.2. Study Characteristics

The 69 studies included in the review comprised 71 interventions. One psychosocial study had two eligible intervention arms [98], and one study included an eligible psychosocial intervention as well as an eligible mindfulness-based intervention [118]. One yoga study (with *n* = 30 participants) was excluded for meta-analysis as only adjusted means were available [75]. Overall, we analyzed 6387 participants with post-intervention fatigue assessments (*n* = 1726 in yoga interventions; *n* = 3214 in psychosocial interventions; *n* = 1467 in mindfulness-based interventions; *n* = 20 participants of the control group of the study by Sheikhzadeh et al. are counted twice [118]).

The majority of studies included solely or mainly breast cancer patients (76.0% for yoga interventions; 58.1% for psychosocial interventions, 86.6% for mindfulness-based interventions). Yoga and psychosocial interventions were predominantly conducted (for the majority of participants) during cancer treatment (56% of yoga interventions; 67.7% of psychosocial interventions), while this was only the case in 26.6% of mindfulness-based interventions. Duration of interventions ranged from 3–12 weeks for yoga interventions with *M* = 11.56 sessions, from 2–52 weeks for psychosocial interventions with *M* = 6.1 sessions, and from one day to 12 weeks for mindfulness-based interventions with *M* = 6.4 sessions. Control groups mostly comprised wait-list control groups for yoga interventions (72.0%) and mindfulness-based interventions (66.7%), and standard care for psychosocial interventions (67.7%). Tables S2.1, S2.2, and S2.3 in the Supplementary Materials provide further details of all included studies.

### 3.3. Risk of Bias

Regarding the risk of bias criteria, random sequence generation and allocation concealment were assessed as inadequate or unclear in 40.6% and 59.4% of psychosocial interventions, in 33.3% and 50% of yoga interventions, and 33.3% and 53.3% of mindfulness-based interventions, respectively. Due to the nature of included studies involving interventions that were all obvious to both participants and personnel, we argue that neither of them could be blinded (i.e., high risk in all studies), nor was it possible to blind participants due to self-reporting of CRF (i.e., high risk in all studies). Incomplete outcome data were assessed with unclear or high risk in 12.5% of psychosocial, 20% of yoga, and 6.7% of mindfulness-based intervention studies, with selective reporting with regard to fatigue in none of the studies. Supplementary Section S2 displays bias assessments for all studies, separated by intervention type.

### 3.4. Effects of Interventions on CRF

**Figure 2:**
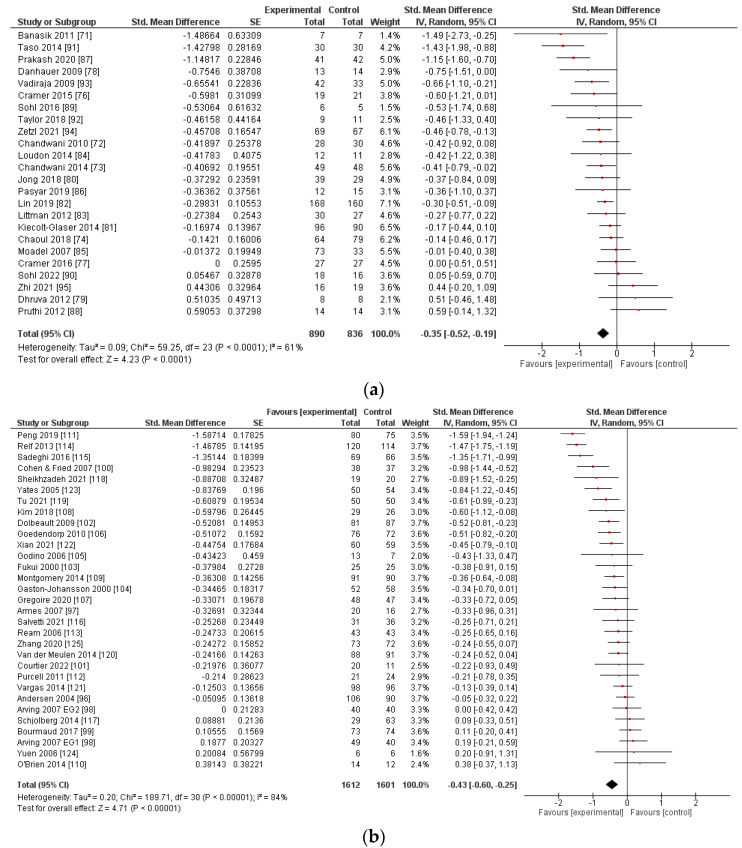

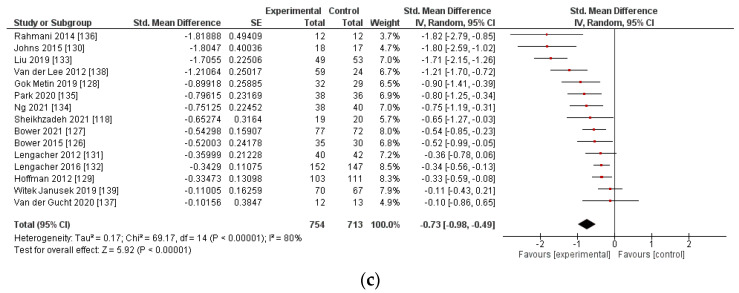
Forest plot of standardized mean differences between baseline and post-intervention fatigue for (**a**) yoga interventions, (**b**) psychosocial interventions, and (**c**) mindfulness-based interventions.

All three intervention types showed an overall significant reduction in CRF (Figure 2). The largest effect was observed for mindfulness-based interventions with an SMD of −0.73 (95% CI −0.98, −0.49, *p* < 0.001, *n* = 15 studies). Psychosocial interventions yielded a significant SMD of −0.43 (95% CI −0.60, −0.25, *p* < 0.001, *n* = 31). A significant effect was also observed for yoga interventions with an SMD of −0.35 (95% CI −0.52, −0.19, *p* < 0.001, *n* = 24).

The calculated prediction intervals for true effects are −0.99 to 0.29 for yoga interventions, −1.36 to 0.50 for psychosocial interventions, and −1.66 to 0.20 for mindfulness-based interventions (see Supplementary Section S1). This means that the effect of any future intervention (in comparable populations) has a 95% probability of falling within the respective range. Thus, there is large heterogeneity with most interventions yielding small to moderate effect sizes; some may result in high effects, and some might even have no beneficial effects on CRF. Therefore, we explore in the following which characteristics might determine successful interventions.

### 3.5. Intervention Characteristics Associated with Intervention Efficacy

#### 3.5.1. Yoga Interventions

Variable selection: The following characteristics were included in the subsequent models: breathing techniques, mental practice, physical effort, variety between sessions, group setting, and as a fixed covariate, total intervention time. Yoga styles were not included because a wide variety of styles were used in the yoga interventions, i.e., this variable had many categories with very small numbers; focus on CRF was omitted as no yoga program was specifically designed to reduce CRF. No bivariate correlation was higher than 0.8 (all intercorrelations between variables are shown in Supplementary Section S4 Figure S4.1).

Model Selection: Testing all possible variable combinations to predict yoga intervention effects on CRF yielded 19 meta-regression models that were within six units of the AICc of the best model (range AICc: 39.04–44.91; weights: 0.18–0.01). There was no intervention characteristic that showed a significant relationship with intervention efficacy in any model. The best five models according to AICc are documented in Table S3.1.

**Figure 3:**
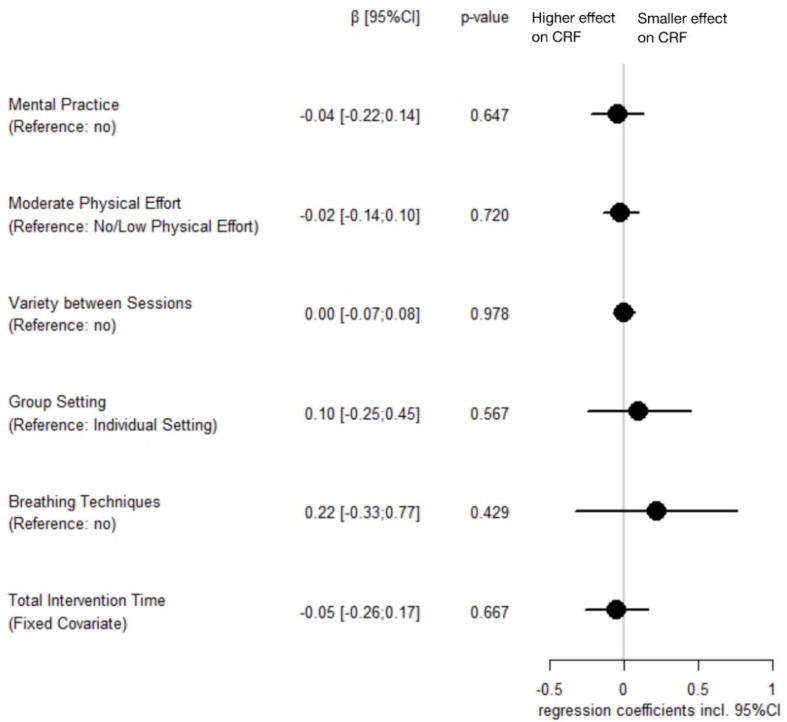
Model-averaged parameter estimates of characteristics of yoga interventions regarding their effect on cancer-related fatigue (including 95% confidence interval). Note: Estimates were computed and weighted based on 19 models that were within six units of the corrected Akaike information criterion of the best model; *total intervention time* was z-standardized; CI = confidence interval; CRF = cancer-related fatigue.

Model Averaging: No analyzed characteristic of yoga interventions showed a significant association with intervention efficacy when considering regression models within six units of the AICc of the best model. Weighted model-averaged regression coefficients are shown in Figure 3.

#### 3.5.2. Psychosocial Interventions

Variable selection: The following variables were included in the subsequent models: CRF education, work on cognition, work on behavior, work on emotions, social resources, relaxation, focus on CRF, group setting, and as a fixed covariate, total intervention time. Hypnosis was only used in two interventions, so it was not included in subsequent analysis. No bivariate correlation was higher than 0.8 (intercorrelations are shown in Supplementary Section S4 Figure S4.2).

*Model Selection:* Testing all possible variable combinations to predict psychosocial intervention effects on CRF yielded 11 meta-regression models that were within six units of the AICc of the best model (range AICc: 33.15–39.14; weights: 0.30–0.01). Within all the top five models, *group setting* and *work on cognition* were significantly associated with a higher intervention efficacy, whereas *relaxation* was associated with a smaller intervention efficacy (see Table S3.2).

**Figure 4:**
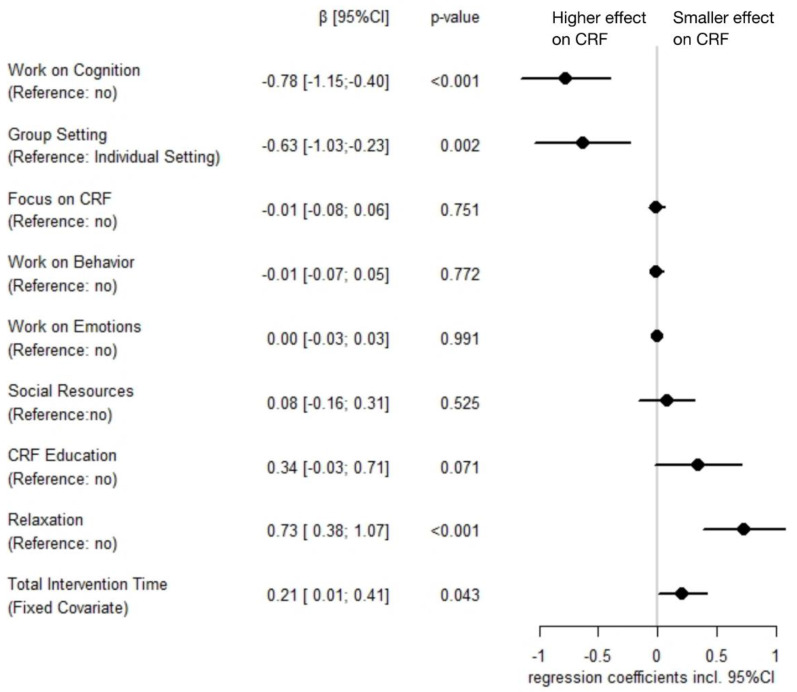
Model-averaged parameter estimates of characteristics of psychosocial interventions regarding their effect on cancer-related fatigue (including 95% confidence interval). *Note:* Estimates were computed and weighted based on the 11 models that were within six units of the corrected Akaike information criterion of the best model; *total intervention time* was z-standardized; CI = confidence interval; CRF = cancer-related fatigue.

*Model Averaging:* The results of the model averaging show that *group setting* and *work on cognition* were significantly associated with higher intervention effects in reducing CRF. In contrast, *relaxation* was significantly related to smaller intervention effects. There was a trend for *CRF education* being likewise linked to smaller intervention effects. *Total intervention time* as a fixed covariate for all models was also significantly associated with smaller intervention effects. Weighted model-averaged regression coefficients are shown in Figure 4.

#### 3.5.3. Mindfulness-Based Interventions

*Variable selection:* The following variables were included in the subsequent models: *CRF education, work on cognition, yoga exercises, group setting,* and as a fixed covariate, *total intervention time*. *Mediation* was not included in the models as all mindfulness-based interventions used some form of meditation. The additional fixed covariate *CRF as an inclusion criterion* had a correlation of *r* = 1.0 with a *focus on CRF*, so we omitted the latter variable. No correlation between any other two variables exceeded a correlation coefficient of 0.8 (see Supplementary Section S4 Figure S4.3).

*Model selection:* Testing all possible variable combinations to predict mindfulness-based intervention effects on CRF yielded five meta-regression models that were within six units of the AICc of the best model (range AICc: 32.66–37.30; weights: 0.65–0.06). There was no intervention characteristic that showed a significant relationship with intervention efficacy in any model. The four models (apart from the intercept-only model) within six units of the AICc of the best model are documented in Table S3.3.

**Figure 5:**
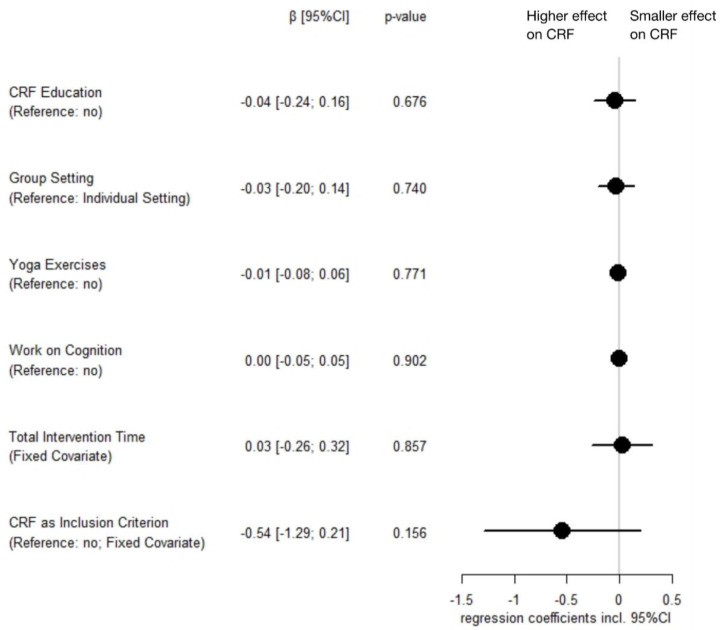
Model-averaged parameter estimates of characteristics of mindfulness-based interventions regarding their effect on cancer-related fatigue (including 95% confidence interval). Note: Estimates were computed and weighted based on the five models that were within six units of the corrected Akaike information criterion of the best model; *total intervention time* was z-standardized; CI = confidence interval; CRF = cancer-related fatigue.

*Model Averaging:* No analyzed characteristic of mindfulness-based interventions showed a significant association with intervention efficacy when considering the regression models within six units of the AICc of the best model. Weighted model-averaged regression coefficients are shown in Figure 5.

### 3.6. Publication Bias

Visual inspections of the funnel plots were conducted for all intervention types, indicating an overall symmetrical pattern despite few outliers with high effects (but rather low standard errors) for yoga interventions (Supplementary Section S5, Figure S5.1) and psychosocial interventions (Supplementary Section S5, Figure S5.2). The plot for mindfulness-based interventions appears asymmetric (Supplementary Section S5, Figure S5.3), as studies with the lowest standard errors showed no or low effects while the two studies with the highest effects had high standard errors.

Egger’s test that examines funnel plot asymmetry yielded non-significant results for psychosocial interventions (95% CI −1.13, 4.85; *p* = 0.232) and yoga interventions (95% CI −1.89, 1.20; *p* = 0.667). Regarding mindfulness-based interventions, Egger’s test indicated asymmetry (95% CI −6.67, −0.37; *p* = 0.047) which may point to a publication bias.

### 3.7. Sensitivity Analyses

Sensitivity analyses revealed no significant difference in standardized mean differences when comparing overall effects of yoga, psychosocial, and mindfulness-based interventions with effects when excluding studies with a high risk of bias, with study populations less than 25 patients per intervention arm, or when excluding studies with specific study populations (see Supplementary Section S6). For yoga interventions, there was a trend for different effects with regard to types of controls, i.e., for interventions with standard care as control group (SMD = −0.77; 95% CI 1.23, −0.31), with a wait-list control group (SMD = −0.23; 95% CI 0.38, −0.07), or attention control (SMD = −0.08; 95% CI −0.64, 0.49; X^2^(2, *n* = 23) = 5.28, *p* = 0.007).

## 4. Discussion

This review and meta-analysis showed that yoga interventions, psychosocial interventions, and mindfulness-based interventions are effective in reducing CRF in cancer patients. To our knowledge, this meta-analysis is the first to assemble characteristics of interventions for CRF in such detail and to analyze them simultaneously using multimodel inference. Our results suggest that psychosocial interventions may have larger beneficial effects on CRF if they include work on cognition and are conducted in a group setting. In contrast, in mindfulness-based interventions, neither providing the intervention in a group setting nor any other intervention characteristic emerged as being more essential or advantageous than others. The same holds for yoga interventions.

The overall efficacy of yoga, psychosocial, and mindfulness-based interventions on CRF found in this meta-analysis is consistent with previous meta-analyses [15,16,19,35,58]. At the same time, our analyses suggest that the overall effects should be interpreted cautiously. Regarding mindfulness interventions, the funnel plot and the result of the Egger test point to a small-study effect, potentially due to a publication bias. Thus, despite the strongest mean effect of mindfulness-based interventions on CRF, further study results should be awaited to obtain a more comprehensive picture. In addition, our results showed high heterogeneity of effects across all three included intervention types. In previous meta-analyses, heterogeneity between studies has only been represented in terms of the I^2^-index. However, I^2^ only indicates the extent of the inconsistency of findings across included studies [50], which is why the use of prediction intervals is advocated [141]. Prediction intervals allow the identification of precise ranges for the effect that can be expected for a future intervention (in a comparable study population). Remarkably, we found that these prediction intervals considerably exceeded 0 for all intervention types. Thus, because of the large heterogeneity between interventions, these cannot guarantee a reducing effect on CRF for future study populations. Therefore, it is crucial to determine the characteristics of interventions that increase the likelihood of a beneficial intervention effect on CRF.

Previous meta-analyses that attempted to identify beneficial intervention characteristics mainly performed separate subgroup comparisons for each characteristic, thus ignoring potential confounding by other associated characteristics. Our multimodel inference approach allowed us to test variables across different model combinations to determine their importance. The psychosocial interventions included in the meta-analysis involved a large variety of contents and modalities. Accounting for the different characteristics, including work on cognition in psychosocial intervention, appeared to result in increased effects on CRF. Work on cognition comprised coping techniques, questioning beliefs and thinking patterns as well as cognitive restructuring. The found importance of work on cognition may be due to the role of cognition in the genesis and manifestation of CRF. Empirical findings underlined that CRF is associated with feelings of helplessness [142], rumination [143], and unfavorable coping strategies, especially catastrophizing [144] that showed a reciprocal and mutually reinforcing relationship with CRF [145]. A recent study that analyzed the mechanisms of three cognitive-behavioral interventions on CRF showed that their effects were explained in part by a reduction in catastrophizing thoughts [146]. Interestingly, only 7 of the 21 interventions in our meta-analysis that addressed cognition had a focus on CRF. In contrast to previous reviews that argued for interventions specifically for CRF [58,147], our analyses did not reveal a focus on CRF as a favorable characteristic for intervention effectiveness on CRF. Our result might be explained by the fact that there was also enough room to address CRF-relevant cognition in non-fatigue-specific interventions or that cognition does not need to be addressed specifically in relation to CRF as it is also relevant for other psychosocial problems of cancer patients, or that by reducing these other psychosocial problems CRF might also be reduced. This seems an intriguing question for future research pursuing mechanisms of interventions against CRF.

In contrast to a recent review that highlighted an advantage of the general cognitive-behavioral approach for reducing CRF [147], we analyzed the intervention elements’ work on cognition and work on behavior separately. In doing so, we allowed for a wide range of possible behavioral modification techniques, which included goal setting and problem-solving but also the promotion and scheduling of (physical) activity, which is closely related to CRF [148,149]. This approach resulted in only 3 of the 31 interventions not applying work on behavior which may explain the non-significant result of this intervention element in our analysis. However, splitting the behavioral techniques would have led to overparameterization. If more psychosocial interventions sufficiently expand the database in the future, a separate analysis of behavioral techniques, preferably based on the established taxonomy of behavior change techniques [150], would certainly be useful.

Further, our results indicate that it is favorable for psychosocial interventions to be delivered in groups rather than individually. Group-based approaches enable exchange between group members, which can create a sense of community [151,152]. CRF is still an unfamiliar symptom among many individuals suffering from it [153], so sharing the symptom burden with others affected could be relieving. This form of group cohesion was observed and highlighted by the authors of two highly effective interventions included in this meta-analysis [111,115], which may offer an important reason for their effects. Since group-based interventions are generally a cost-efficient alternative to individual interventions [154], there is a strong case for targeting their benefits for future interventions.

Relaxation techniques emerged in our analyses as a factor associated with lower efficacy for CRF. A previous meta-analysis found a mitigating effect of relaxation interventions on CRF [155], while another meta-analysis suggested that this may be limited to patient populations during therapy [15]. Our analyses did not include relaxation-only procedures but examined relaxation techniques as part of psychosocial interventions. In our analysis, only 3 of the 13 interventions that used relaxation techniques were reported using the well-established progressive muscle relaxation technique by Jacobsen [156]. The other interventions either did not specify the relaxation procedure or used guided relaxing imagery or specific relaxation tasks. Since our analyses can only be evaluated in conjunction with the other included variables, we would also like to refrain from interpreting our results meaning that relaxation techniques cannot be a beneficial component of intervention against CRF. However, the session time may be used more efficiently with techniques other than relaxation, such as work on cognition. We interpret the result of a smaller effect associated with CRF education (although not significant) similarly. There are still large gaps in knowledge of CRF on the patient side [153], which can be reduced, for example, with information provision [157]. For an effective reduction in CRF, additional intervention techniques that support patients in the form of knowledge translation strategies are probably helpful [158].

The yoga interventions included in this review and meta-analysis could not be sharply distinguished from each other as they largely share similar components, i.e., require at least some physical effort and are performed with breathing awareness. On the other hand, yoga interventions comprised a wide range of yoga programs with Hatha yoga as the most commonly used style, but Iyengar yoga, Tibetan yoga, restorative yoga, Dru yoga, and self-composed yoga programs were also applied. Due to the small number of studies per style (apart from Hatha), the database was insufficient to compare individual yoga styles. Regarding the other intervention characteristics, none of them emerged in our analyses as being more advantageous for reducing CRF than others. In line with our results, recent reviews did not identify specific yoga characteristics that determine the intervention effect on CRF [159,160]. In contrast, Armer and Lutgendorf found that interventions applying yoga styles with physical poses produced higher effects than nonphysical yoga types, such as pranayama interventions [21]. In addition to a slightly different study selection, we categorized physical effort according to the yoga postures indicated rather than the yoga style, which may explain this discrepant result. In a recent (not cancer-specific) review, Cramer et al. compared 53 different yoga styles in 306 randomized controlled trials and did not find a difference in their effect on different health outcomes [161]. Consistent with this finding, our result that the effect of yoga interventions on CRF is independent of specific intervention characteristics suggests that different forms of yoga practices promise to be beneficial and could be selected based on personal preferences. This conclusion is subject to the limitation of a comparatively small number of yoga interventions that investigated the effect of CRF in cancer patients and may be adapted to changing evidence.

Similarly, in mindfulness-based interventions, no significant differences in effects on CRF by intervention characteristics emerged. We are only aware of one review that conducted subgroup comparisons in mindfulness-based interventions: Duong and colleagues found a trend for greater effectiveness of mindfulness-based interventions that included only study participants with pre-existing CRF [155]. We used CRF as an inclusion criterion as a fixed covariate, and although it was not significant in multimodel analysis, it had the strongest regression coefficient of all included variables. Of course, this may be due to a statistical regression-to-the-mean effect, since there is more room for improvement in case of higher initial values. Nonetheless, for future mindfulness-based interventions, it seems promising to analyze whether mindfulness may be more beneficial for treating existing CRF than for preventing its onset. Our analyses did not identify higher effectiveness on CRF of mindfulness-based interventions applying working with cognition in accordance with the mindfulness-based cognitive therapy [32]. However, the database is still small. Thus, given our finding of advantages of using cognitive techniques in psychosocial interventions and given that the effects of mindfulness-based interventions work primarily through emotional and cognitive processes [162,163], increased consideration of how to integrate cognitive techniques into mindfulness-based approaches may nevertheless be promising.

As a general finding, it is noteworthy that the total intervention time was not significantly related to higher intervention effects on CRF for any intervention type but was associated with a deteriorating effect for psychosocial interventions. This surprising result is subject to the limitation that not all studies reported participants’ adherence. For the sake of consistency, we based the analyses on the planned intervention time rather than the intervention time performed by participants. Future research could address the question of whether longer intervention time is related to lower adherence, potentially negating the effects of intervention content. What can be concluded on the basis of this meta-analysis is that intervention content and modalities seem more important than the extent or duration of intervention.

### Strengths and Limitations

This systematic review and meta-analysis included a high number of psychosocial, mindfulness-based, and yoga interventions that assessed CRF as the outcome. However, considering the wide spectrum of different intervention characteristics, especially with regard to mindfulness-based and yoga interventions, the multivariate analyses might have been limited by an insufficient database in detecting the effects of intervention characteristics. Therefore, we also could not model interactions between different intervention characteristics as this would have resulted in excessive model complexity given the available data. Due to this risk of overparameterization, but also due to insufficient documentation of intervention characteristics in the literature, we could not include all potentially relevant intervention characteristics in the analyses. Therefore, other intervention characteristics that were not part of this meta-analysis (such as role of the intervention provider or homework/home practice) may further explain intervention effects.

Another limitation of this review was the difficulty in classifying selected intervention characteristics according to descriptions of interventions in original papers. Due to inaccurate information, some classifications had to be estimated. Nevertheless, when necessary, information was supplemented by direct inquiries to the authors, and classifications in this review were carefully evaluated by at least two reviewers and discussed with other experts when considered helpful.

Despite its size, the database of included studies for the review and meta-analysis could be selective in terms of included study populations as well as search strategies. Although we did not restrict our systematic research to any cancer types, the majority of the included study populations were breast cancer patients, potentially limiting the generalizability for other cancer types. However, neither our results nor those of other reviews [15,16] suggest differential intervention effects on CRF for different cancer types. Our search strategy was based in part on existing reviews and solely on articles published in English in peer-reviewed journals. Therefore, there may be additional studies written in other languages or not published because of null findings that might have contributed to a more complete picture of interventions for CRF.

This meta-analysis identified a large dispersion in intervention effects implying that future interventions cannot guarantee an average reduction in CRF for their study populations. Nevertheless, our analyses gave valuable insights into some characteristics of psychosocial interventions associated with a favorable effect on CRF. An increase in the database through future published intervention studies will make multivariate statistical approaches such as multimodel inference more valuable, especially regarding yoga and mindfulness-based interventions. In addition to analyzing intervention characteristics, future research efforts should be devoted to explaining the effect mechanisms of interventions to base future interventions on verifiable assumptions. Although CRF is increasingly recognized as a multidimensional construct consisting of a cognitive, affective, and physical subdimension [164], only a small number of interventions have assessed these subdimensions. In addition, an insufficient number of studies have calculated the long-term effects of interventions on CRF. For future meta-analyses that will be able to draw on a larger number of studies, it will also be relevant to examine the significance of intervention characteristics for subdimensions of CRF as well as for long-term effects.

## 5. Conclusions

This review and meta-analysis underline that yoga, psychosocial, and mindfulness-based interventions are effective therapeutic measures to reduce CRF. Nevertheless, not every intervention necessarily delivers a significant effect on CRF. With regard to psychosocial interventions, effective components appear to be a group setting as well as applying cognitive techniques, whereas relaxation techniques do not seem to provide any additional benefit. In contrast, for both yoga and mindfulness-based interventions, no specific intervention characteristic emerged as being more essential or advantageous than others. Overall, longer intervention duration did not appear to be more efficient than shorter interventions. The findings of our review and meta-analysis highlight opportunities for psychosocial interventions to become more effective but at the same time point to the need for further research particularly on how to design optimal yoga and mindfulness-based interventions.

## Data Availability

No new data were created or analyzed in this study. Data sharing is not applicable to this article.

## Supplementary Materials

The following are available online at https://www.mdpi.com/article/10.3390/cancers14082016/s1, Section S1: Subgroup comparisons, Section S2: Risk of bias assessments, Section S3: Prediction intervals, Section S4: Intercorrelations between intervention characteristics, Section S5: Funnel plots, Section S6: Sensitivity analyses, Tables S1: Search strategies, Tables S2: Description of interventions, Tables S3: Meta-regression. Refs. [71,72,73,74,75,76,77,78,79,80,81,82,83,84,85,86,87,88,89,90,91,92,93,94,95,96,97,98,99,100,101,102,103,104,105,106,107,108,109,110,111,112,113,114,115,116,117,118,119,120,121,122,123,124,125,126,127,128,129,130,131,132,133,134,135,136,137,138,139] are cited in the Supplementary material.
