# Supplementary material for: Meta-Analysis of Randomized Controlled Trials on Yoga, Psychosocial, and Mindfulness-Based Interventions for Cancer-Related Fatigue: What Intervention Characteristics Are Related to Higher Efficacy?
Source: Cancers (Basel). 2022 Apr 15;14(8):2016. doi: 10.3390/cancers14082016 (PMC9032769; doi:10.3390/cancers14082016)
Supplement: Supplementary file 1 [file cancers-14-02016-s001.zip › Supplementary Section S1_subgroup comparisons_Proof_V2.pdf]

Section S.1.1. Yoga Interventions

Study or Subgroup	Std. Mean Difference	SE	Experimental Total	Control Total	Weight	Std. Mean Difference IV, Random, 95% CI
**1.16.1 Breast Cancer (≥50% of study population)**						
Banasik 2011 [71]	-1.48664	0.63309	7	7	1.4%	-1.49 [-2.73, -0.25]
Taso 2014 [91]	-1.42798	0.28169	30	30	4.2%	-1.43 [-1.98, -0.88]
Prakash 2020 [87]	-1.14817	0.22846	41	42	5.0%	-1.15 [-1.60, -0.70]
Danhauer 2009 [78]	-0.7546	0.38708	13	14	2.9%	-0.75 [-1.51, 0.00]
Vadiraja 2009 [93]	-0.65541	0.22836	42	33	5.0%	-0.66 [-1.10, -0.21]
Cramer 2015 [76]	-0.5981	0.31099	19	21	3.8%	-0.60 [-1.21, 0.01]
Taylor 2018 [92]	-0.46158	0.44164	9	11	2.5%	-0.46 [-1.33, 0.40]
Chandwani 2010 [72]	-0.41897	0.25378	28	30	4.6%	-0.42 [-0.92, 0.08]
Loudon 2014 [84]	-0.41783	0.4075	12	11	2.8%	-0.42 [-1.22, 0.38]
Chandwani 2014 [73]	-0.40692	0.19551	49	48	5.6%	-0.41 [-0.79, -0.02]
Jong 2018 [80]	-0.37292	0.23591	39	29	4.9%	-0.37 [-0.84, 0.09]
Pasyar 2019 [86]	-0.36362	0.37561	12	15	3.1%	-0.36 [-1.10, 0.37]
Lin 2019 [82]	-0.29831	0.10553	168	160	7.1%	-0.30 [-0.51, -0.09]
Littman 2012 [83]	-0.27384	0.2543	30	27	4.6%	-0.27 [-0.77, 0.22]
Kiecolt-Glaser 2014 [81]	-0.16974	0.13967	96	90	6.5%	-0.17 [-0.44, 0.10]
Chaoul 2018 [74]	-0.1421	0.16006	64	79	6.2%	-0.14 [-0.46, 0.17]
Moadel 2007 [85]	-0.01372	0.19949	73	33	5.5%	-0.01 [-0.40, 0.38]
Zhi 2021 [95]	0.44306	0.32964	16	19	3.6%	0.44 [-0.20, 1.09]
Dhruva 2012 [79]	0.51035	0.49713	8	8	2.1%	0.51 [-0.46, 1.48]
Pruthi 2012 [88]	0.59053	0.37298	14	14	3.1%	0.59 [-0.14, 1.32]
**Subtotal (95% CI)**			**770**	**721**	**84.3%**	**-0.38 [-0.57, -0.19]**
Heterogeneity: Tau² = 0.10; Chi² = 55.51, df = 19 (P < 0.0001); I² = 66%						
Test for overall effect: Z = 3.96 (P < 0.0001)						
**1.16.2 Other (>50% of study population other than breast cancer)**						
Sohl 2016 [89]	-0.53064	0.61632	6	5	1.5%	-0.53 [-1.74, 0.68]
Zetzl 2021 [94]	-0.45708	0.16547	69	67	6.1%	-0.46 [-0.78, -0.13]
Cramer 2016 [77]	0	0.2595	27	27	4.5%	0.00 [-0.51, 0.51]
Sohl 2022 [90]	0.05467	0.32878	18	16	3.6%	0.05 [-0.59, 0.70]
**Subtotal (95% CI)**			**120**	**115**	**15.7%**	**-0.25 [-0.54, 0.03]**
Heterogeneity: Tau² = 0.01; Chi² = 3.51, df = 3 (P = 0.32); I² = 15%						
Test for overall effect: Z = 1.75 (P = 0.08)						
**Total (95% CI)**			**890**	**836**	**100.0%**	**-0.35 [-0.52, -0.19]**
Heterogeneity: Tau² = 0.09; Chi² = 59.25, df = 23 (P < 0.0001); I² = 61%						
Test for overall effect: Z = 4.23 (P < 0.0001)						
Test for subgroup differences: Chi² = 0.53, df = 1 (P = 0.47), I² = 0%						

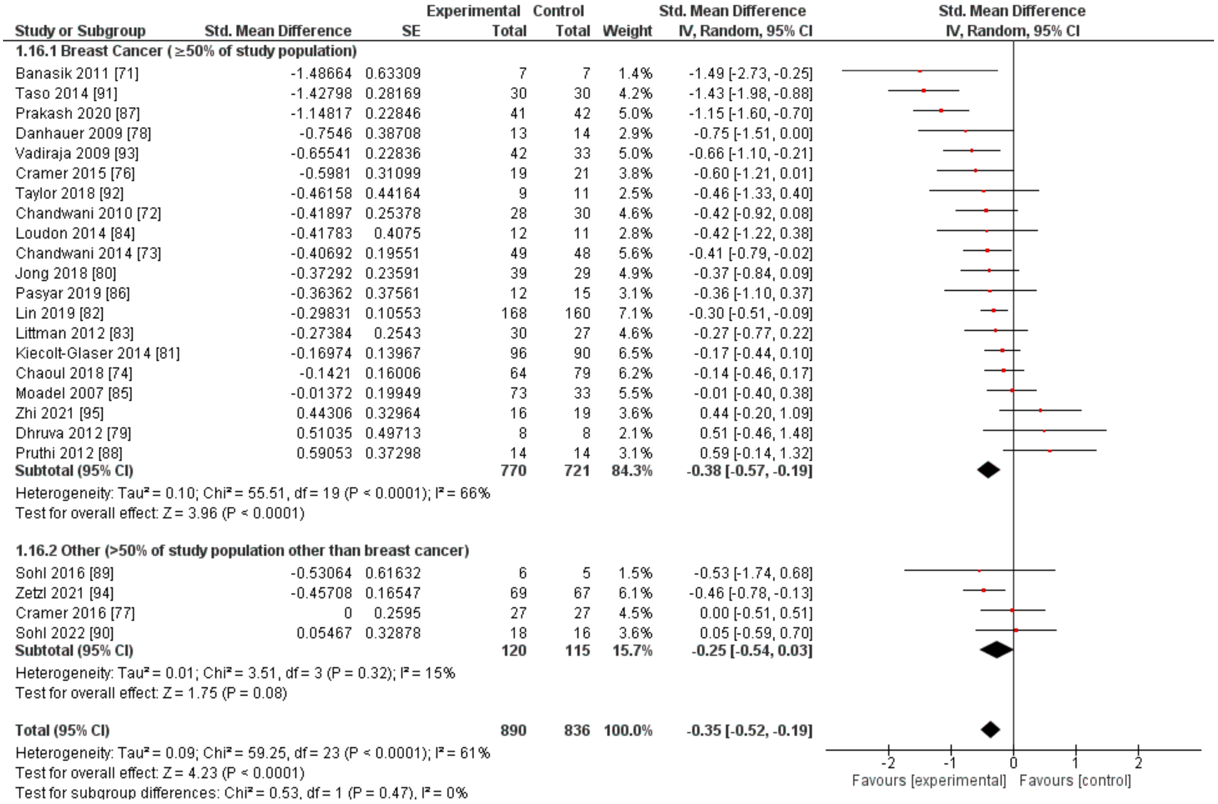

Figure S1.1.1. Comparison of yoga interventions regarding entity of study population (at least 50% of study population having breast cancer vs. other).

Study or Subgroup	Std. Mean Difference	SE	Experimental Total	Control Total	Weight	Std. Mean Difference IV, Random, 95% CI
**1.15.1 During Treatment (≥50% of study population)**						
Taso 2014 [91]	-1.42798	0.28169	30	30	4.2%	-1.43 [-1.98, -0.88]
Prakash 2020 [87]	-1.14817	0.22846	41	42	5.0%	-1.15 [-1.60, -0.70]
Danhauer 2009 [78]	-0.7546	0.38708	13	14	2.9%	-0.75 [-1.51, 0.00]
Vadiraja 2009 [93]	-0.65541	0.22836	42	33	5.0%	-0.66 [-1.10, -0.21]
Sohl 2016 [89]	-0.53064	0.61632	6	5	1.5%	-0.53 [-1.74, 0.68]
Zetzl 2021 [94]	-0.45708	0.16547	69	67	6.1%	-0.46 [-0.78, -0.13]
Chandwani 2010 [72]	-0.41897	0.25378	28	30	4.6%	-0.42 [-0.92, 0.08]
Chandwani 2014 [73]	-0.40692	0.19551	49	48	5.6%	-0.41 [-0.79, -0.02]
Jong 2018 [80]	-0.37292	0.23591	39	29	4.9%	-0.37 [-0.84, 0.09]
Chaoul 2018 [74]	-0.1421	0.16006	64	79	6.2%	-0.14 [-0.46, 0.17]
Sohl 2022 [90]	0.05467	0.32878	18	16	3.6%	0.05 [-0.59, 0.70]
Dhruva 2012 [79]	0.51035	0.49713	8	8	2.1%	0.51 [-0.46, 1.48]
Pruthi 2012 [88]	0.59053	0.37298	14	14	3.1%	0.59 [-0.14, 1.32]
**Subtotal (95% CI)**			**421**	**415**	**54.7%**	**-0.44 [-0.70, -0.18]**
Heterogeneity: Tau² = 0.15; Chi² = 40.58, df = 12 (P < 0.0001); I² = 70%						
Test for overall effect: Z = 3.29 (P = 0.001)						
**1.15.2 After Treatment (>50% of study population)**						
Banasik 2011 [71]	-1.48664	0.63309	7	7	1.4%	-1.49 [-2.73, -0.25]
Cramer 2015 [76]	-0.5981	0.31099	19	21	3.8%	-0.60 [-1.21, 0.01]
Taylor 2018 [92]	-0.46158	0.44164	9	11	2.5%	-0.46 [-1.33, 0.40]
Loudon 2014 [84]	-0.41783	0.4075	12	11	2.8%	-0.42 [-1.22, 0.38]
Pasyar 2019 [86]	-0.36362	0.37561	12	15	3.1%	-0.36 [-1.10, 0.37]
Lin 2019 [82]	-0.29831	0.10553	168	160	7.1%	-0.30 [-0.51, -0.09]
Littman 2012 [83]	-0.27384	0.2543	30	27	4.6%	-0.27 [-0.77, 0.22]
Kiecolt-Glaser 2014 [81]	-0.16974	0.13967	96	90	6.5%	-0.17 [-0.44, 0.10]
Moadel 2007 [85]	-0.01372	0.19949	73	33	5.5%	-0.01 [-0.40, 0.38]
Cramer 2016 [77]	0	0.2595	27	27	4.5%	0.00 [-0.51, 0.51]
Zhi 2021 [95]	0.44306	0.32964	16	19	3.6%	0.44 [-0.20, 1.09]
**Subtotal (95% CI)**			**469**	**421**	**45.3%**	**-0.22 [-0.38, -0.06]**
Heterogeneity: Tau² = 0.01; Chi² = 12.71, df = 10 (P = 0.24); I² = 21%						
Test for overall effect: Z = 2.67 (P = 0.008)						
**Total (95% CI)**			**890**	**836**	**100.0%**	**-0.35 [-0.52, -0.19]**
Heterogeneity: Tau² = 0.09; Chi² = 59.25, df = 23 (P < 0.0001); I² = 61%						
Test for overall effect: Z = 4.23 (P < 0.0001)						
Test for subgroup differences: Chi² = 1.97, df = 1 (P = 0.16), I² = 49.2%						

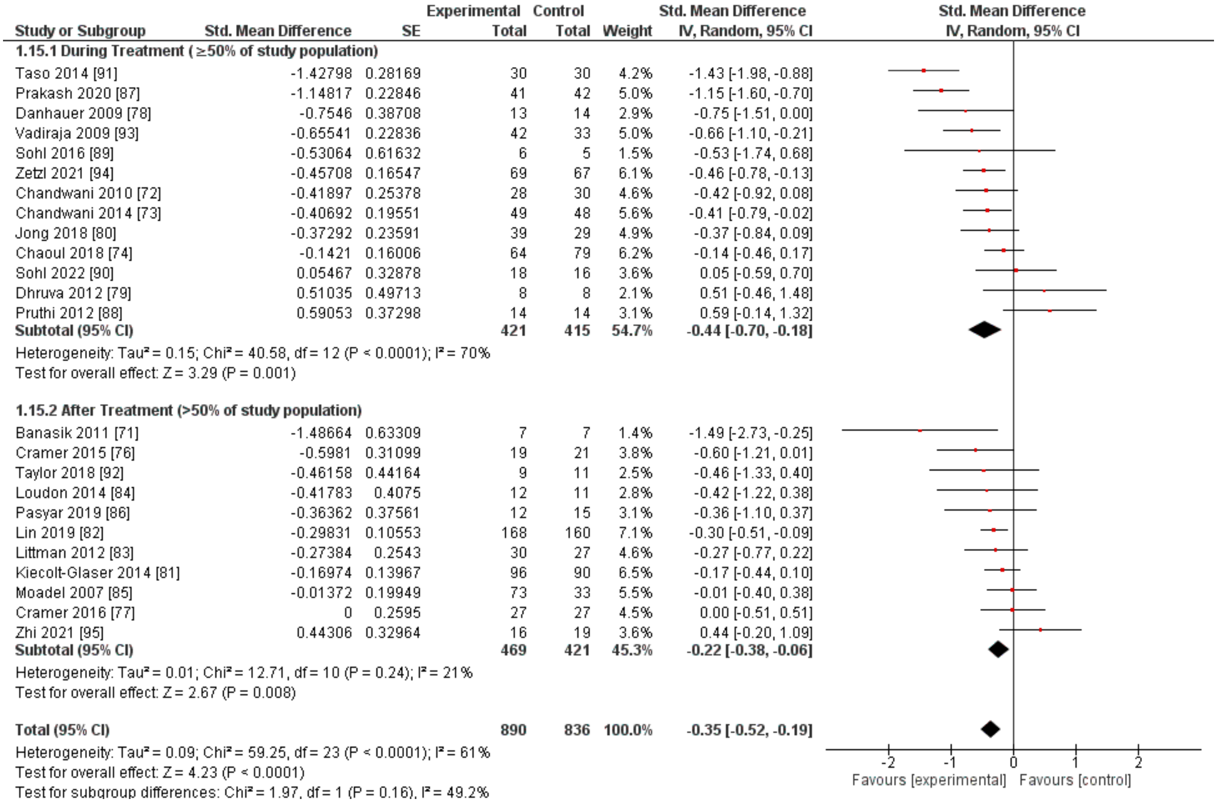

Figure S1.1.2. Comparison of yoga interventions regarding treatment status of study population (at least 50% of study population during treatment vs. other).

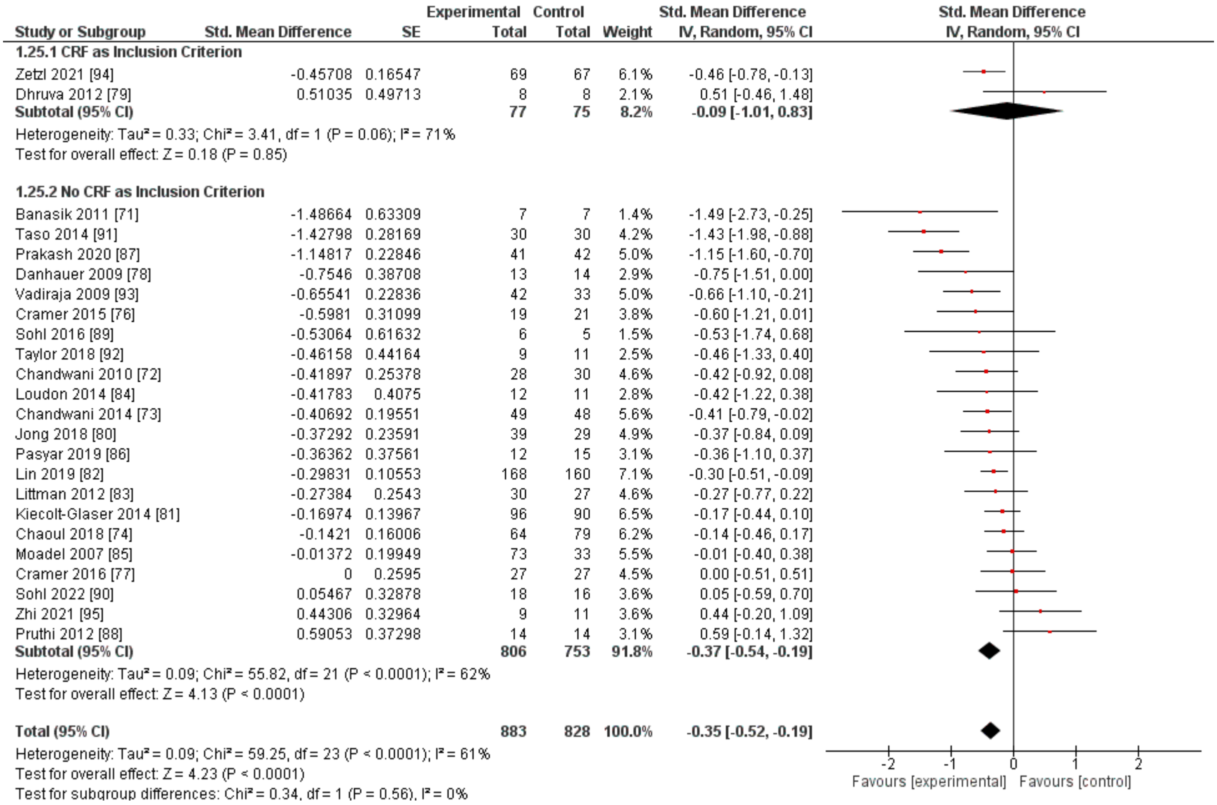

Figure S1.1.3. Comparison of yoga interventions regarding whether a specific level of cancer-related fatigue was required as an inclusion criterion.

Note: CRF = cancer-related fatigue.

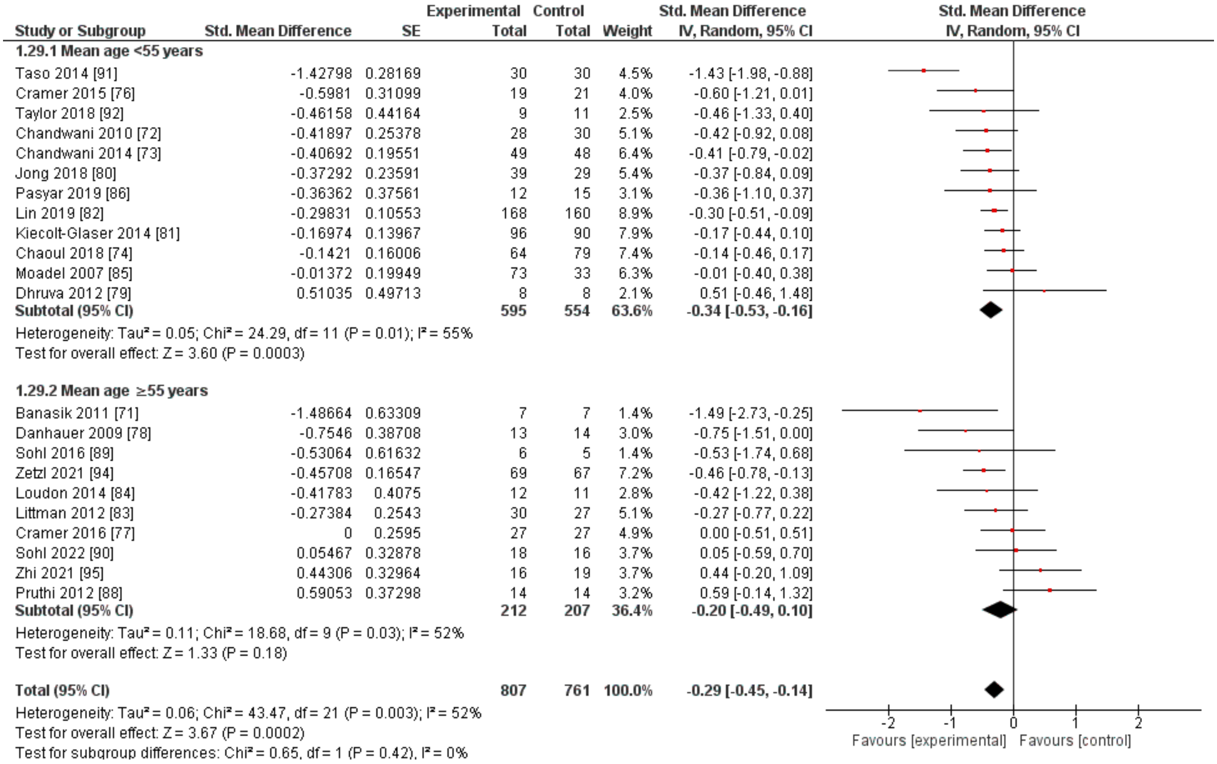

Figure S1.1.4. Comparison of yoga interventions regarding age of study population (cut-off was oriented towards median (=54.3 years)).

Note: Two studies ((Prakash (2020) [87] and Vadiraya (2009) [93]) did not report a mean age.

Section S1.2. Psychosocial Interventions

Study or Subgroup	Std. Mean Difference	SE	Experimental Total	Control Total	Weight	Std. Mean Difference IV, Random, 95% CI
**1.5.1 Breast Cancer (≥50% of Study Population)**						
Reif 2013 [114]	-1.46785	0.14195	120	114	3.7%	-1.47 [-1.75, -1.19]
Sadeghi 2016 [115]	-1.35144	0.18399	69	66	3.5%	-1.35 [-1.71, -0.99]
Cohen & Fried 2007 [100]	-0.98294	0.23523	38	37	3.2%	-0.98 [-1.44, -0.52]
Sheikhzadeh 2021 [118]	-0.88708	0.32487	19	20	2.7%	-0.89 [-1.52, -0.25]
Yates 2005 [123]	-0.83769	0.196	50	54	3.4%	-0.84 [-1.22, -0.45]
Kim 2018 [108]	-0.59796	0.26445	29	26	3.0%	-0.60 [-1.12, -0.08]
Dolbeault 2009 [102]	-0.52081	0.14953	81	87	3.7%	-0.52 [-0.81, -0.23]
Fukui 2000 [103]	-0.37984	0.2728	25	25	3.0%	-0.38 [-0.91, 0.15]
Montgomery 2014 [109]	-0.36308	0.14256	91	90	3.7%	-0.36 [-0.64, -0.08]
Gaston-Johansson 2000 [104]	-0.34465	0.18317	52	58	3.5%	-0.34 [-0.70, 0.01]
Gregoire 2020 [107]	-0.33071	0.19678	48	47	3.4%	-0.33 [-0.72, 0.05]
Courtier 2022 [101]	-0.21976	0.36077	20	11	2.5%	-0.22 [-0.93, 0.49]
Vargas 2014 [121]	-0.12503	0.13656	98	96	3.7%	-0.13 [-0.39, 0.14]
Andersen 2004 [96]	-0.05095	0.13618	106	90	3.7%	-0.05 [-0.32, 0.22]
Arving 2007 EG2 [98]	0	0.21283	40	40	3.3%	0.00 [-0.42, 0.42]
Schjolberg 2014 [117]	0.08881	0.2136	29	63	3.3%	0.09 [-0.33, 0.51]
Bourmaud 2017 [99]	0.10555	0.1569	73	74	3.6%	0.11 [-0.20, 0.41]
Arving 2007 EG1 [98]	0.1877	0.20327	49	40	3.4%	0.19 [-0.21, 0.59]
O'Brien 2014 [110]	0.38143	0.38221	14	12	2.4%	0.38 [-0.37, 1.13]
**Subtotal (95% CI)**			**1051**	**1050**	**62.8%**	**-0.41 [-0.66, -0.17]**
Heterogeneity: Tau² = 0.24; Chi² = 140.31, df = 18 (P < 0.00001); I² = 87%						
Test for overall effect: Z = 3.35 (P = 0.0008)						
**1.5.3 Other (>50% of Study Population other than Breast Cancer)**						
Peng 2019 [111]	-1.58714	0.17825	80	75	3.5%	-1.59 [-1.94, -1.24]
Tu 2021 [119]	-0.60879	0.19534	50	50	3.4%	-0.61 [-0.99, -0.23]
Goedendorp 2010 [106]	-0.51072	0.1592	76	72	3.6%	-0.51 [-0.82, -0.20]
Xian 2021 [122]	-0.44754	0.17684	60	59	3.5%	-0.45 [-0.79, -0.10]
Godino 2006 [105]	-0.43423	0.459	13	7	2.0%	-0.43 [-1.33, 0.47]
Armes 2007 [97]	-0.32691	0.32344	20	16	2.7%	-0.33 [-0.96, 0.31]
Salvetti 2021 [116]	-0.25268	0.23449	31	36	3.2%	-0.25 [-0.71, 0.21]
Ream 2006 [113]	-0.24733	0.20615	43	43	3.4%	-0.25 [-0.65, 0.16]
Zhang 2020 [125]	-0.24272	0.15852	73	72	3.6%	-0.24 [-0.55, 0.07]
Van der Meulen 2014 [120]	-0.24166	0.14263	88	91	3.7%	-0.24 [-0.52, 0.04]
Purcell 2011 [112]	-0.214	0.28623	21	24	2.9%	-0.21 [-0.78, 0.35]
Yuen 2006 [124]	0.20084	0.56799	6	6	1.6%	0.20 [-0.91, 1.31]
**Subtotal (95% CI)**			**561**	**551**	**37.2%**	**-0.45 [-0.71, -0.19]**
Heterogeneity: Tau² = 0.15; Chi² = 48.86, df = 11 (P < 0.00001); I² = 77%						
Test for overall effect: Z = 3.43 (P = 0.0006)						
**Total (95% CI)**			**1612**	**1601**	**100.0%**	**-0.43 [-0.60, -0.25]**
Heterogeneity: Tau² = 0.20; Chi² = 189.71, df = 30 (P < 0.00001); I² = 84%						
Test for overall effect: Z = 4.71 (P < 0.00001)						
Test for subgroup differences: Chi² = 0.04, df = 1 (P = 0.83), I² = 0%						

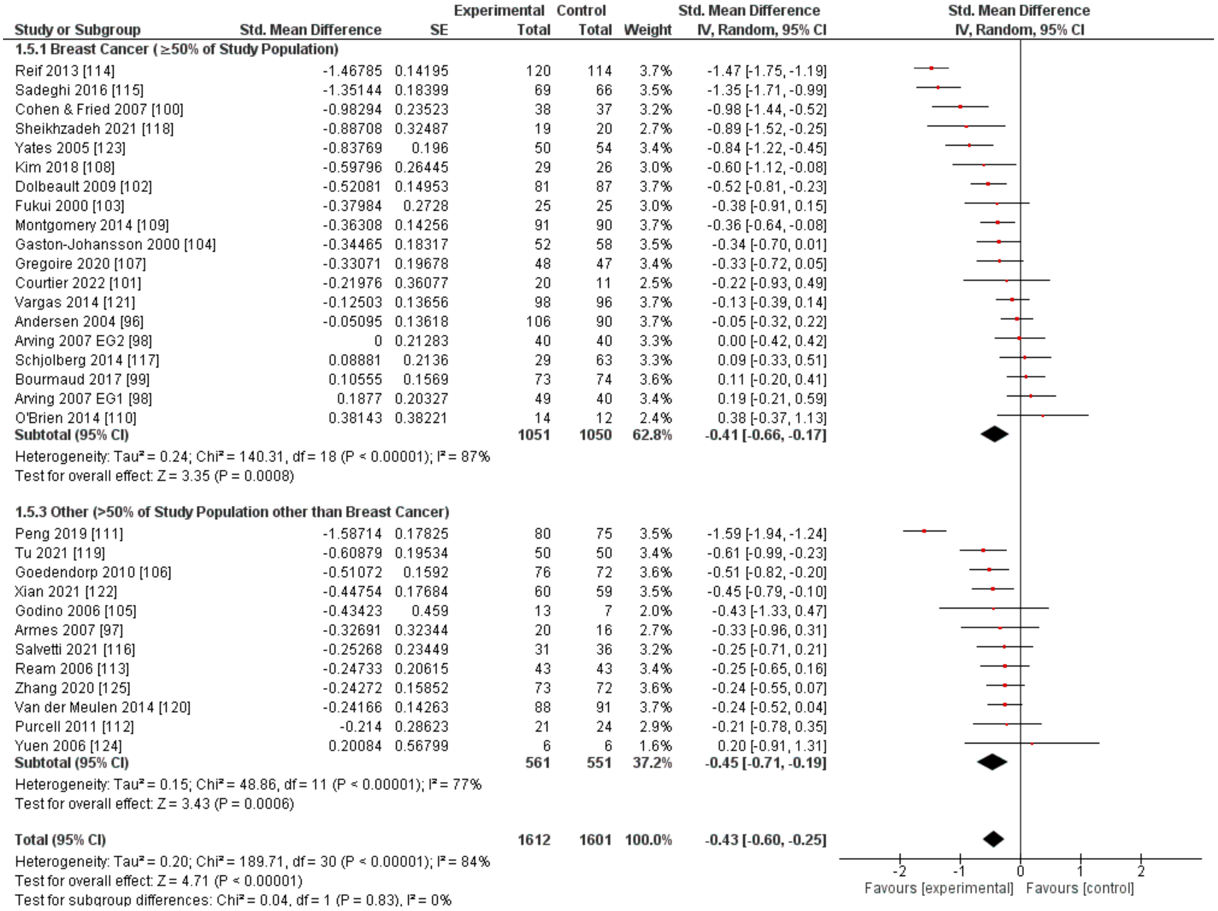

Figure S1.2.1. Comparison of psychosocial interventions regarding entity of study population (at least 50% of study population having breast cancer vs. other).

Study or Subgroup	Std. Mean Difference	SE	Experimental Total	Control Total	Weight	Std. Mean Difference IV, Random, 95% CI
**1.6.1 During Treatment (≥50% of Study Population)**						
Peng 2019 [111]	-1.58714	0.17825	80	75	3.5%	-1.59 [-1.94, -1.24]
Sadeghi 2016 [115]	-1.35144	0.18399	69	66	3.5%	-1.35 [-1.71, -0.99]
Cohen & Fried 2007 [100]	-0.98294	0.23523	38	37	3.2%	-0.98 [-1.44, -0.52]
Yates 2005 [123]	-0.83769	0.196	50	54	3.4%	-0.84 [-1.22, -0.45]
Tu 2021 [119]	-0.60879	0.19534	50	50	3.4%	-0.61 [-0.99, -0.23]
Kim 2018 [108]	-0.59796	0.26445	29	26	3.0%	-0.60 [-1.12, -0.08]
Goedendorp 2010 [106]	-0.51072	0.1592	76	72	3.6%	-0.51 [-0.82, -0.20]
Xian 2021 [122]	-0.44754	0.17684	60	59	3.5%	-0.45 [-0.79, -0.10]
Godino 2006 [105]	-0.43423	0.459	13	7	2.0%	-0.43 [-1.33, 0.47]
Montgomery 2014 [109]	-0.36308	0.14256	91	90	3.7%	-0.36 [-0.64, -0.08]
Gaston-Johansson 2000 [104]	-0.34465	0.18317	52	58	3.5%	-0.34 [-0.70, 0.01]
Armes 2007 [97]	-0.32691	0.32344	20	16	2.7%	-0.33 [-0.96, 0.31]
Salvetti 2021 [116]	-0.25268	0.23449	31	36	3.2%	-0.25 [-0.71, 0.21]
Ream 2006 [113]	-0.24733	0.20615	43	43	3.4%	-0.25 [-0.65, 0.16]
Courtier 2022 [101]	-0.21976	0.36077	20	11	2.5%	-0.22 [-0.93, 0.49]
Purcell 2011 [112]	-0.214	0.28623	21	24	2.9%	-0.21 [-0.78, 0.35]
Vargas 2014 [121]	-0.12503	0.13656	98	96	3.7%	-0.13 [-0.39, 0.14]
Andersen 2004 [96]	-0.05095	0.13618	106	90	3.7%	-0.05 [-0.32, 0.22]
Arving 2007 EG2 [98]	0	0.21283	40	40	3.3%	0.00 [-0.42, 0.42]
Bourmaud 2017 [99]	0.10555	0.1569	73	74	3.6%	0.11 [-0.20, 0.41]
Arving 2007 EG1 [98]	0.1877	0.20327	49	40	3.4%	0.19 [-0.21, 0.59]
O'Brien 2014 [110]	0.38143	0.38221	14	12	2.4%	0.38 [-0.37, 1.13]
**Subtotal (95% CI)**			**1123**	**1076**	**71.3%**	**-0.41 [-0.62, -0.21]**
Heterogeneity: Tau² = 0.19; Chi² = 123.34, df = 21 (P < 0.00001); I² = 83%						
Test for overall effect: Z = 3.95 (P < 0.0001)						
**1.6.2 After Treatment (>50% of Study Population)**						
Reif 2013 [114]	-1.46785	0.14195	120	114	3.7%	-1.47 [-1.75, -1.19]
Sheikhzadeh 2021 [118]	-0.88708	0.32487	19	20	2.7%	-0.89 [-1.52, -0.25]
Dolbeault 2009 [102]	-0.52081	0.14953	81	87	3.7%	-0.52 [-0.81, -0.23]
Fukui 2000 [103]	-0.37984	0.2728	25	25	3.0%	-0.38 [-0.91, 0.15]
Gregoire 2020 [107]	-0.33071	0.19678	48	47	3.4%	-0.33 [-0.72, 0.05]
Zhang 2020 [125]	-0.24272	0.15852	73	72	3.6%	-0.24 [-0.55, 0.07]
Van der Meulen 2014 [120]	-0.24166	0.14263	88	91	3.7%	-0.24 [-0.52, 0.04]
Schjolberg 2014 [117]	0.08881	0.2136	29	63	3.3%	0.09 [-0.33, 0.51]
Yuen 2006 [124]	0.20084	0.56799	6	6	1.6%	0.20 [-0.91, 1.31]
**Subtotal (95% CI)**			**489**	**525**	**28.7%**	**-0.45 [-0.82, -0.09]**
Heterogeneity: Tau² = 0.25; Chi² = 63.61, df = 8 (P < 0.00001); I² = 87%						
Test for overall effect: Z = 2.44 (P = 0.01)						
**Total (95% CI)**			**1612**	**1601**	**100.0%**	**-0.43 [-0.60, -0.25]**
Heterogeneity: Tau² = 0.20; Chi² = 189.71, df = 30 (P < 0.00001); I² = 84%						
Test for overall effect: Z = 4.71 (P < 0.00001)						
Test for subgroup differences: Chi² = 0.04, df = 1 (P = 0.85), I² = 0%						

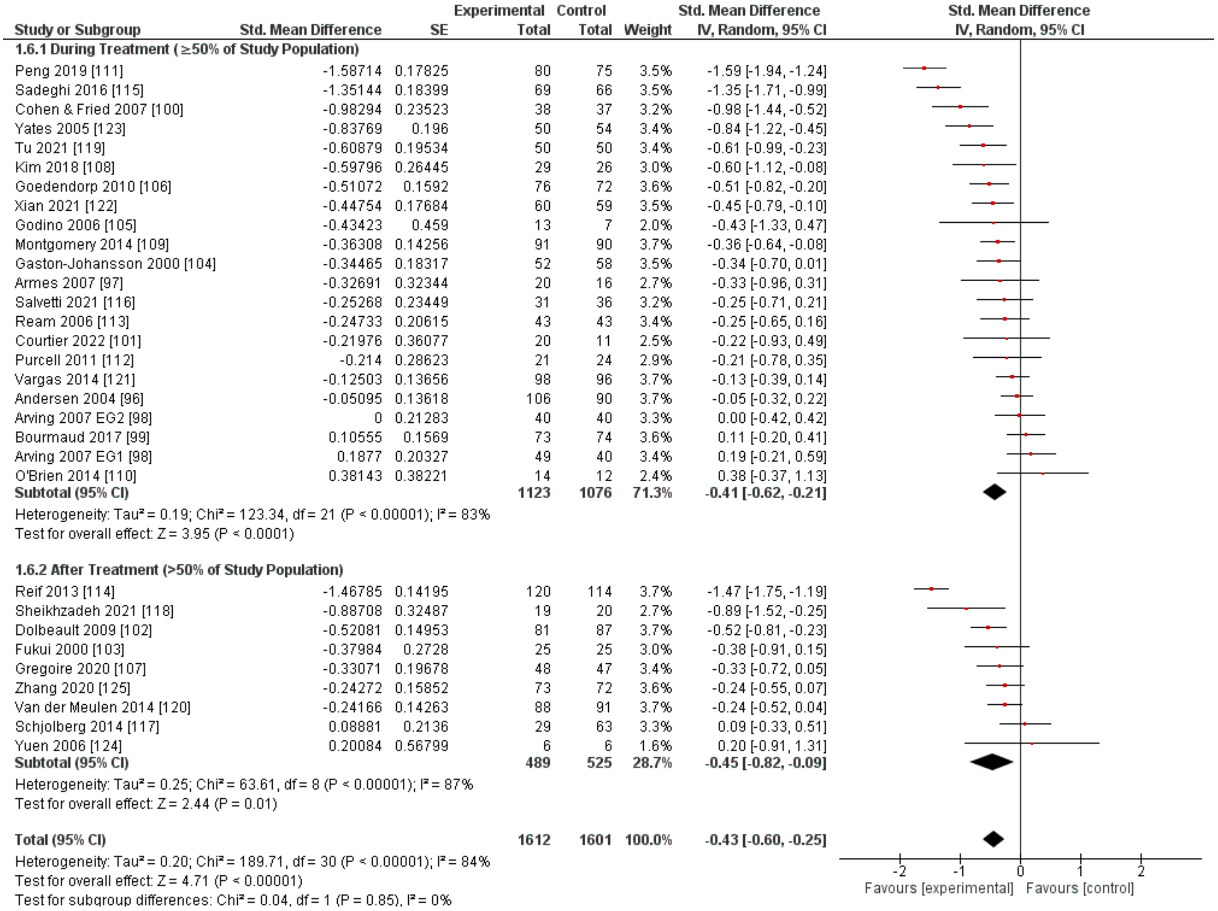

Figure S1.2.2. Comparison of psychosocial interventions regarding treatment status of study population (at least 50% of study population during treatment vs. other).

Study or Subgroup	Std. Mean Difference	SE	Experimental Total	Control Total	Weight	Std. Mean Difference IV, Random, 95% CI
**1.31.1 With CRF as Inclusion Criterion**						
Reif 2013 [114]	-1.46785	0.14195	120	114	3.7%	-1.47 [-1.75, -1.19]
Gregoire 2020 [107]	-0.33071	0.19678	48	47	3.4%	-0.33 [-0.72, 0.05]
Armes 2007 [97]	-0.32691	0.32344	20	16	2.7%	-0.33 [-0.96, 0.31]
Courtier 2022 [101]	-0.21976	0.36077	20	11	2.5%	-0.22 [-0.93, 0.49]
Schjolberg 2014 [117]	0.08881	0.2136	29	63	3.3%	0.09 [-0.33, 0.51]
Bourmaud 2017 [99]	0.10555	0.1569	73	74	3.6%	0.11 [-0.20, 0.41]
Yuen 2006 [124]	0.20084	0.56799	6	6	1.6%	0.20 [-0.91, 1.31]
**Subtotal (95% CI)**			**316**	**331**	**20.8%**	**-0.31 [-0.89, 0.27]**
Heterogeneity: Tau² = 0.53; Chi² = 72.13, df = 6 (P < 0.00001); I² = 92%						
Test for overall effect: Z = 1.04 (P = 0.30)						
**1.31.2 Without CRF as Inclusion Criterion**						
Peng 2019 [111]	-1.58714	0.17825	80	75	3.5%	-1.59 [-1.94, -1.24]
Sadeghi 2016 [115]	-1.35144	0.18399	69	66	3.5%	-1.35 [-1.71, -0.99]
Cohen & Fried 2007 [100]	-0.98294	0.23523	38	37	3.2%	-0.98 [-1.44, -0.52]
Sheikhzadeh 2021 [118]	-0.88708	0.32487	19	20	2.7%	-0.89 [-1.52, -0.25]
Yates 2005 [123]	-0.83769	0.196	50	54	3.4%	-0.84 [-1.22, -0.45]
Tu 2021 [119]	-0.60879	0.19534	50	50	3.4%	-0.61 [-0.99, -0.23]
Kim 2018 [108]	-0.59796	0.26445	29	26	3.0%	-0.60 [-1.12, -0.08]
Dolbeault 2009 [102]	-0.52081	0.14953	81	87	3.7%	-0.52 [-0.81, -0.23]
Goedendorp 2010 [106]	-0.51072	0.1592	76	72	3.6%	-0.51 [-0.82, -0.20]
Xian 2021 [122]	-0.44754	0.17684	60	59	3.5%	-0.45 [-0.79, -0.10]
Godino 2006 [105]	-0.43423	0.459	13	7	2.0%	-0.43 [-1.33, 0.47]
Fukui 2000 [103]	-0.37984	0.2728	25	25	3.0%	-0.38 [-0.91, 0.15]
Montgomery 2014 [109]	-0.36308	0.14256	91	90	3.7%	-0.36 [-0.64, -0.08]
Gaston-Johansson 2000 [104]	-0.34465	0.18317	52	58	3.5%	-0.34 [-0.70, 0.01]
Salvetti 2021 [116]	-0.25268	0.23449	31	36	3.2%	-0.25 [-0.71, 0.21]
Ream 2006 [113]	-0.24733	0.20615	43	43	3.4%	-0.25 [-0.65, 0.16]
Zhang 2020 [125]	-0.24272	0.15852	73	72	3.6%	-0.24 [-0.55, 0.07]
Van der Meulen 2014 [120]	-0.24166	0.14263	88	91	3.7%	-0.24 [-0.52, 0.04]
Purcell 2011 [112]	-0.214	0.28623	21	24	2.9%	-0.21 [-0.78, 0.35]
Vargas 2014 [121]	-0.12503	0.13656	98	96	3.7%	-0.13 [-0.39, 0.14]
Andersen 2004 [96]	-0.05095	0.13618	106	90	3.7%	-0.05 [-0.32, 0.22]
Arving 2007 EG2 [98]	0	0.21283	40	40	3.3%	0.00 [-0.42, 0.42]
Arving 2007 EG1 [98]	0.1877	0.20327	49	40	3.4%	0.19 [-0.21, 0.59]
O'Brien 2014 [110]	0.38143	0.38221	14	12	2.4%	0.38 [-0.37, 1.13]
**Subtotal (95% CI)**			**1296**	**1270**	**79.2%**	**-0.45 [-0.63, -0.27]**
Heterogeneity: Tau² = 0.15; Chi² = 117.01, df = 23 (P < 0.00001); I² = 80%						
Test for overall effect: Z = 5.02 (P < 0.00001)						
**Total (95% CI)**			**1612**	**1601**	**100.0%**	**-0.43 [-0.60, -0.25]**
Heterogeneity: Tau² = 0.20; Chi² = 189.71, df = 30 (P < 0.00001); I² = 84%						
Test for overall effect: Z = 4.71 (P < 0.00001)						
Test for subgroup differences: Chi² = 0.21, df = 1 (P = 0.65), I² = 0%						

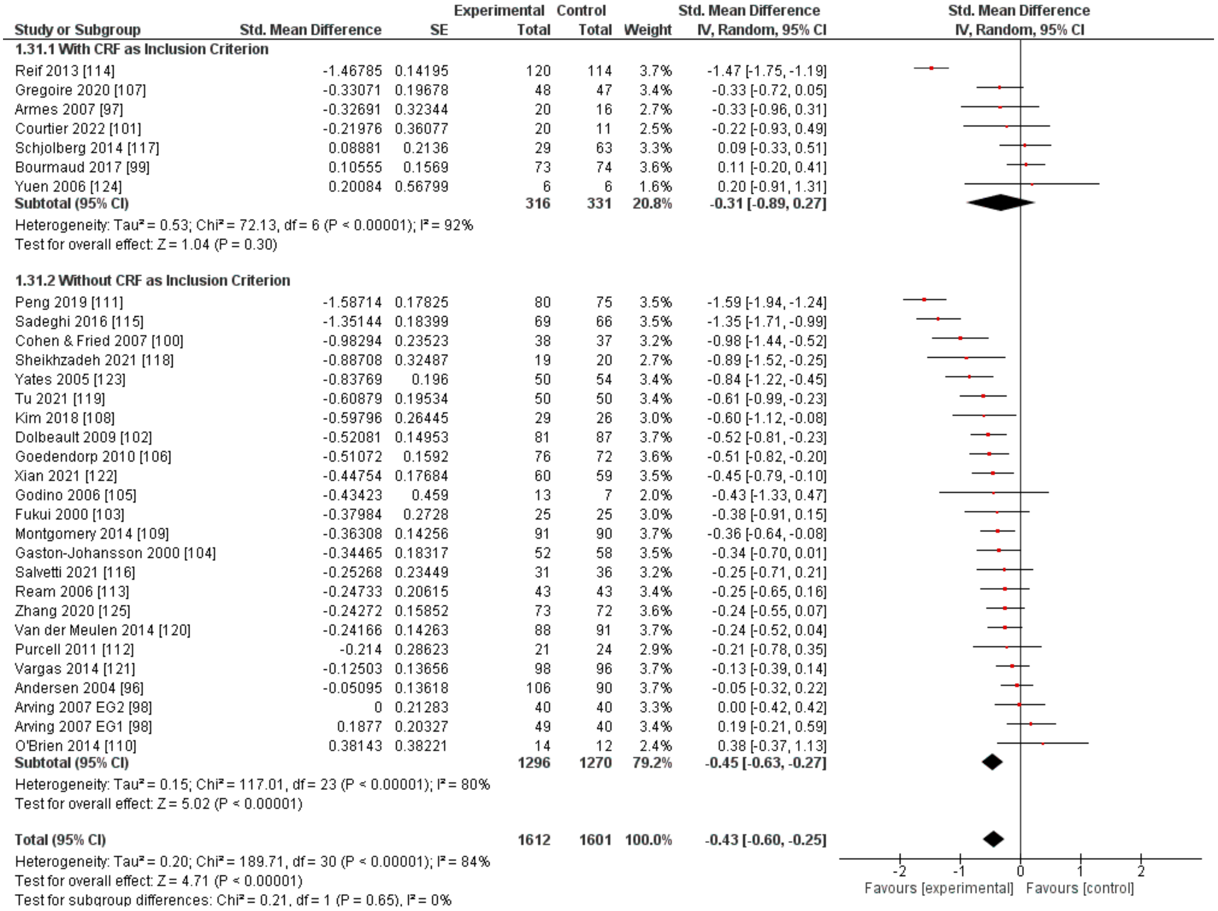

Figure S1.2.3. Comparison of psychosocial interventions regarding whether a specific level of CRF was required as an inclusion criterion.

Note: CRF = cancer-related fatigue.

Study or Subgroup	Std. Mean Difference	SE	Experimental Total	Control Total	Weight	Std. Mean Difference IV, Random, 95% CI
**1.39.1 Mean Age <55 years**						
Cohen & Fried 2007 [100]	-0.98294	0.23523	38	37	3.3%	-0.98 [-1.44, -0.52]
Sheikhzadeh 2021 [118]	-0.88708	0.32487	19	20	2.8%	-0.89 [-1.52, -0.25]
Yates 2005 [123]	-0.83769	0.196	50	54	3.5%	-0.84 [-1.22, -0.45]
Kim 2018 [108]	-0.59796	0.26445	29	26	3.1%	-0.60 [-1.12, -0.08]
Dolbeault 2009 [102]	-0.52081	0.14953	81	87	3.8%	-0.52 [-0.81, -0.23]
Fukui 2000 [103]	-0.37984	0.2728	25	25	3.1%	-0.38 [-0.91, 0.15]
Gregoire 2020 [107]	-0.33071	0.19678	48	47	3.5%	-0.33 [-0.72, 0.05]
Vargas 2014 [121]	-0.12503	0.13656	98	96	3.9%	-0.13 [-0.39, 0.14]
Andersen 2004 [96]	-0.05095	0.13618	106	90	3.9%	-0.05 [-0.32, 0.22]
**Subtotal (95% CI)**			**494**	**482**	**31.0%**	**-0.48 [-0.71, -0.25]**
Heterogeneity: Tau² = 0.08; Chi² = 25.34, df = 8 (P = 0.001); I² = 68%						
Test for overall effect: Z = 4.15 (P < 0.0001)						
**1.39.2 Mean Age ≥55 years**						
Peng 2019 [111]	-1.58714	0.17825	80	75	3.6%	-1.59 [-1.94, -1.24]
Reif 2013 [114]	-1.46785	0.14195	120	114	3.8%	-1.47 [-1.75, -1.19]
Sadeghi 2016 [115]	-1.35144	0.18399	69	66	3.6%	-1.35 [-1.71, -0.99]
Tu 2021 [119]	-0.60879	0.19534	50	50	3.6%	-0.61 [-0.99, -0.23]
Goedendorp 2010 [106]	-0.51072	0.1592	76	72	3.7%	-0.51 [-0.82, -0.20]
Xian 2021 [122]	-0.44754	0.17684	60	59	3.7%	-0.45 [-0.79, -0.10]
Godino 2006 [105]	-0.43423	0.459	13	7	2.1%	-0.43 [-1.33, 0.47]
Montgomery 2014 [109]	-0.36308	0.14256	91	90	3.8%	-0.36 [-0.64, -0.08]
Armes 2007 [97]	-0.32691	0.32344	20	16	2.8%	-0.33 [-0.96, 0.31]
Salvetti 2021 [116]	-0.25268	0.23449	31	36	3.3%	-0.25 [-0.71, 0.21]
Ream 2006 [113]	-0.24733	0.20615	43	43	3.5%	-0.25 [-0.65, 0.16]
Zhang 2020 [125]	-0.24272	0.15852	73	72	3.7%	-0.24 [-0.55, 0.07]
Van der Meulen 2014 [120]	-0.24166	0.14263	88	91	3.8%	-0.24 [-0.52, 0.04]
Courtier 2022 [101]	-0.21976	0.36077	20	11	2.6%	-0.22 [-0.93, 0.49]
Purcell 2011 [112]	-0.214	0.28623	21	24	3.0%	-0.21 [-0.78, 0.35]
Arving 2007 EG2 [98]	0	0.21283	40	40	3.5%	0.00 [-0.42, 0.42]
Schjolberg 2014 [117]	0.08881	0.2136	29	63	3.4%	0.09 [-0.33, 0.51]
Bourmaud 2017 [99]	0.10555	0.1569	73	74	3.8%	0.11 [-0.20, 0.41]
Arving 2007 EG1 [98]	0.1877	0.20327	49	40	3.5%	0.19 [-0.21, 0.59]
Yuen 2006 [124]	0.20084	0.56799	6	6	1.7%	0.20 [-0.91, 1.31]
O'Brien 2014 [110]	0.38143	0.38221	14	12	2.5%	0.38 [-0.37, 1.13]
**Subtotal (95% CI)**			**1066**	**1061**	**69.0%**	**-0.39 [-0.64, -0.14]**
Heterogeneity: Tau² = 0.28; Chi² = 162.89, df = 20 (P < 0.00001); I² = 88%						
Test for overall effect: Z = 3.05 (P = 0.002)						
**Total (95% CI)**			**1560**	**1543**	**100.0%**	**-0.43 [-0.61, -0.25]**
Heterogeneity: Tau² = 0.21; Chi² = 189.38, df = 29 (P < 0.00001); I² = 85%						
Test for overall effect: Z = 4.58 (P < 0.00001)						
Test for subgroup differences: Chi² = 0.29, df = 1 (P = 0.59), I² = 0%						

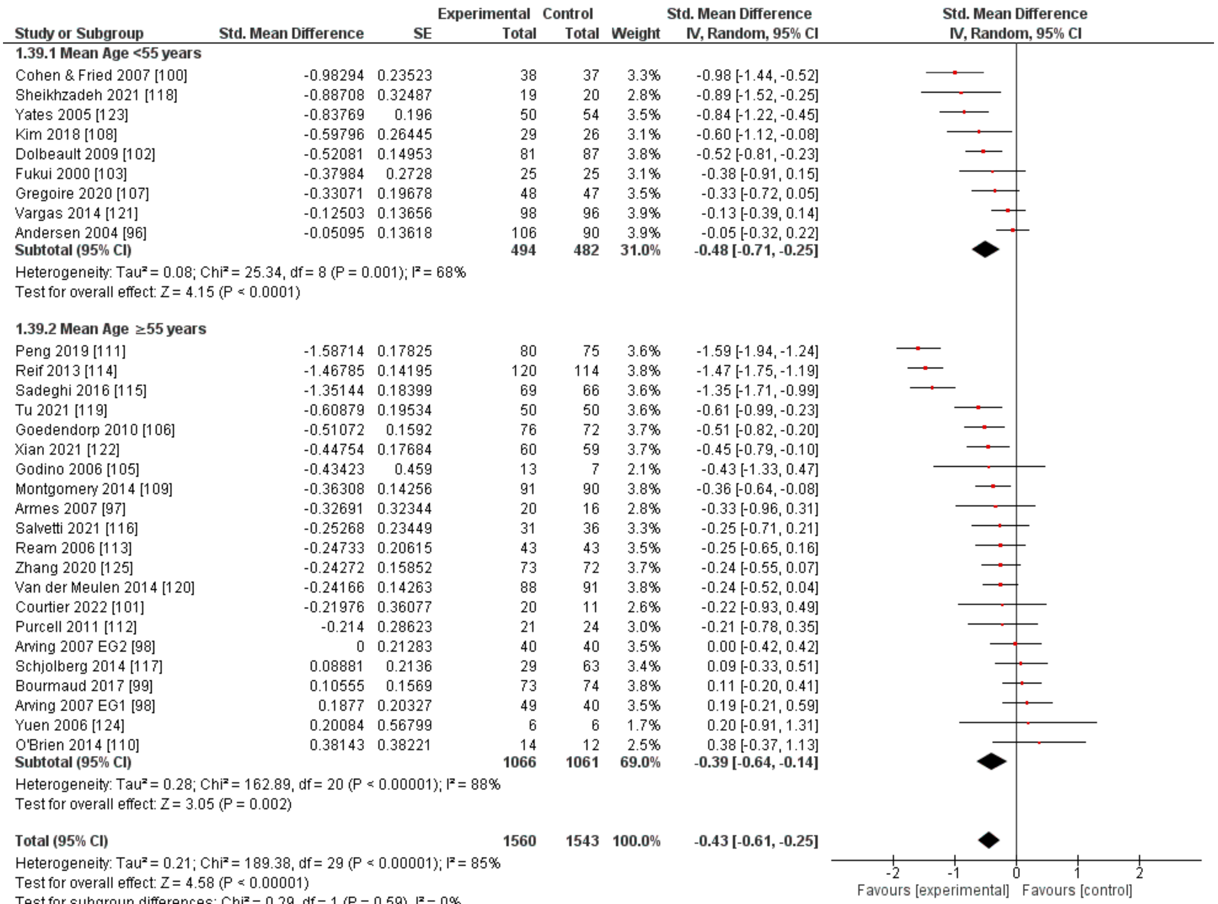

Figure S1.2.4. Comparison of psychosocial interventions regarding age of study population (cut-off was oriented towards median (=55.8 years)).
Note: One study (Gaston-Johansson (2000) [104]) did not report a mean age.

<u>Section S1.3.Mindfulness-based Interventions</u>

Study or Subgroup	Std. Mean Difference	SE	Experimental Total	Control Total	Weight	Std. Mean Difference IV, Random, 95% CI
**1.14.1 Breast Cancer (≥50% of study population)**						
Rahmani 2014 [136]	-1.81888	0.49409	12	12	3.7%	-1.82 [-2.79, -0.85]
Johns 2015 [130]	-1.8047	0.40036	18	17	4.7%	-1.80 [-2.59, -1.02]
Van der Lee 2012 [138]	-1.21064	0.25017	59	24	6.7%	-1.21 [-1.70, -0.72]
Gok Metin 2019 [128]	-0.89918	0.25885	32	29	6.5%	-0.90 [-1.41, -0.39]
Park 2020 [135]	-0.79615	0.23169	38	36	6.9%	-0.80 [-1.25, -0.34]
Sheikhzadeh 2021 [118]	-0.65274	0.3164	19	20	5.7%	-0.65 [-1.27, -0.03]
Bower 2021 [127]	-0.54298	0.15907	35	30	8.0%	-0.54 [-0.85, -0.23]
Bower 2015 [126]	-0.52003	0.24178	77	72	6.8%	-0.52 [-0.99, -0.05]
Lengacher 2012 [131]	-0.35999	0.21228	40	42	7.2%	-0.36 [-0.78, 0.06]
Lengacher 2016 [132]	-0.3429	0.11075	152	147	8.5%	-0.34 [-0.56, -0.13]
Hoffman 2012 [129]	-0.33473	0.13098	103	111	8.3%	-0.33 [-0.59, -0.08]
Witek Janusek 2019 [139]	-0.11005	0.16259	70	67	7.9%	-0.11 [-0.43, 0.21]
Van der Gucht 2020 [137]	-0.10156	0.3847	12	13	4.9%	-0.10 [-0.86, 0.65]
**Subtotal (95% CI)**			**667**	**620**	**85.9%**	**-0.63 [-0.85, -0.41]**
Heterogeneity: Tau² = 0.10; Chi² = 40.98, df = 12 (P < 0.0001); I² = 71%						
Test for overall effect: Z = 5.64 (P < 0.00001)						
**1.14.2 Other (>50% of study population other than breast cancer)**						
Liu 2019 [133]	-1.7055	0.22506	49	53	7.0%	-1.71 [-2.15, -1.26]
Ng 2021 [134]	-0.75125	0.22452	38	40	7.0%	-0.75 [-1.19, -0.31]
**Subtotal (95% CI)**			**87**	**93**	**14.1%**	**-1.23 [-2.16, -0.29]**
Heterogeneity: Tau² = 0.40; Chi² = 9.01, df = 1 (P = 0.003); I² = 89%						
Test for overall effect: Z = 2.57 (P = 0.01)						
**Total (95% CI)**			**754**	**713**	**100.0%**	**-0.73 [-0.98, -0.49]**
Heterogeneity: Tau² = 0.17; Chi² = 69.17, df = 14 (P < 0.00001); I² = 80%						
Test for overall effect: Z = 5.92 (P < 0.00001)						
Test for subgroup differences: Chi² = 1.50, df = 1 (P = 0.22), I² = 33.5%						

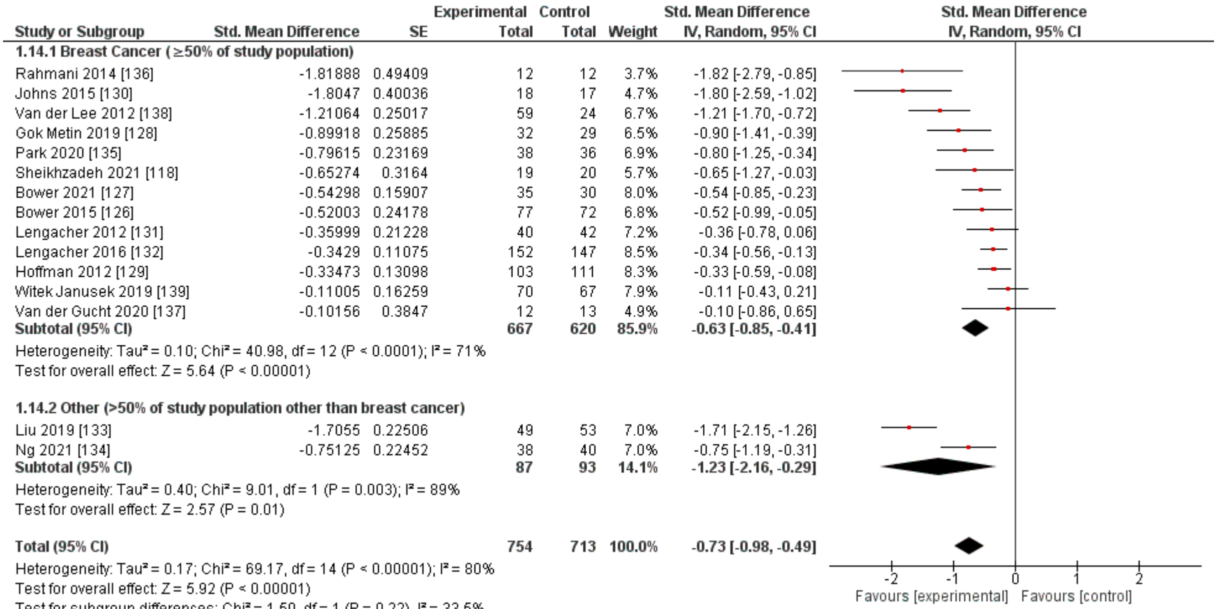

Figure S1.3.1. Comparison of mindfulness-based interventions regarding entity of study population (at least 50% of study population having breast cancer vs. other).

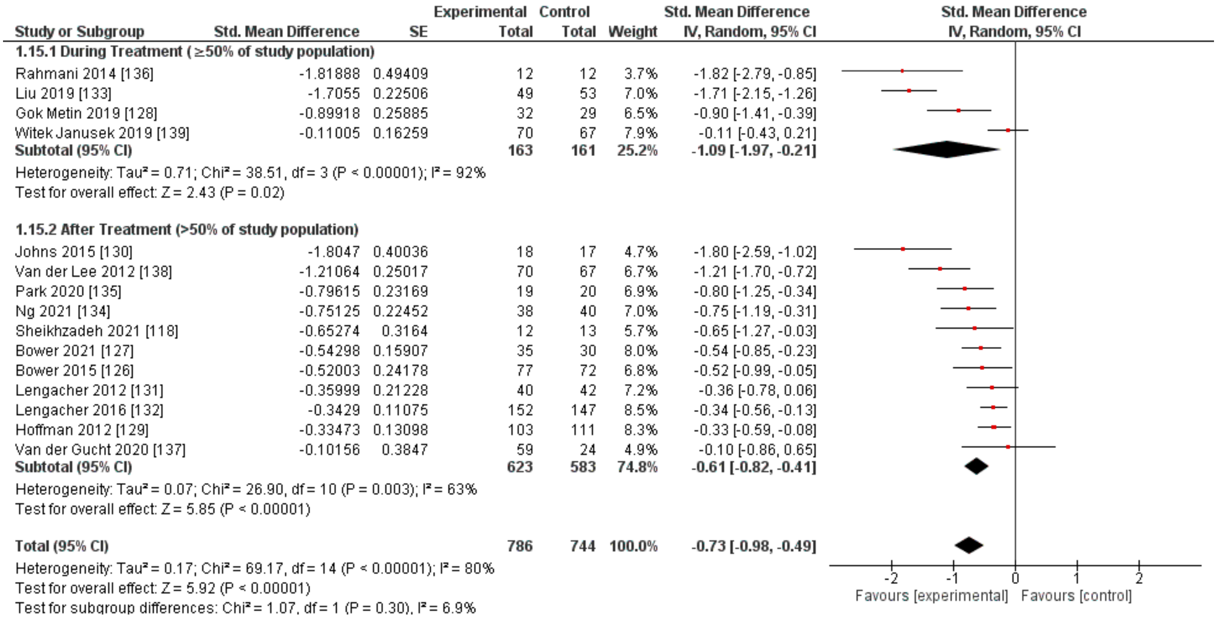

Figure S1.3.2. Comparison of mindfulness-based interventions regarding treatment status of study population (at least 50% of study population during treatment vs. other).

Note: Studies that did not report treatment status (Ng (2021) [134]; Park (2020) [135]; Sheikhzadeh (2021) [118]) were assigned to after treatment.

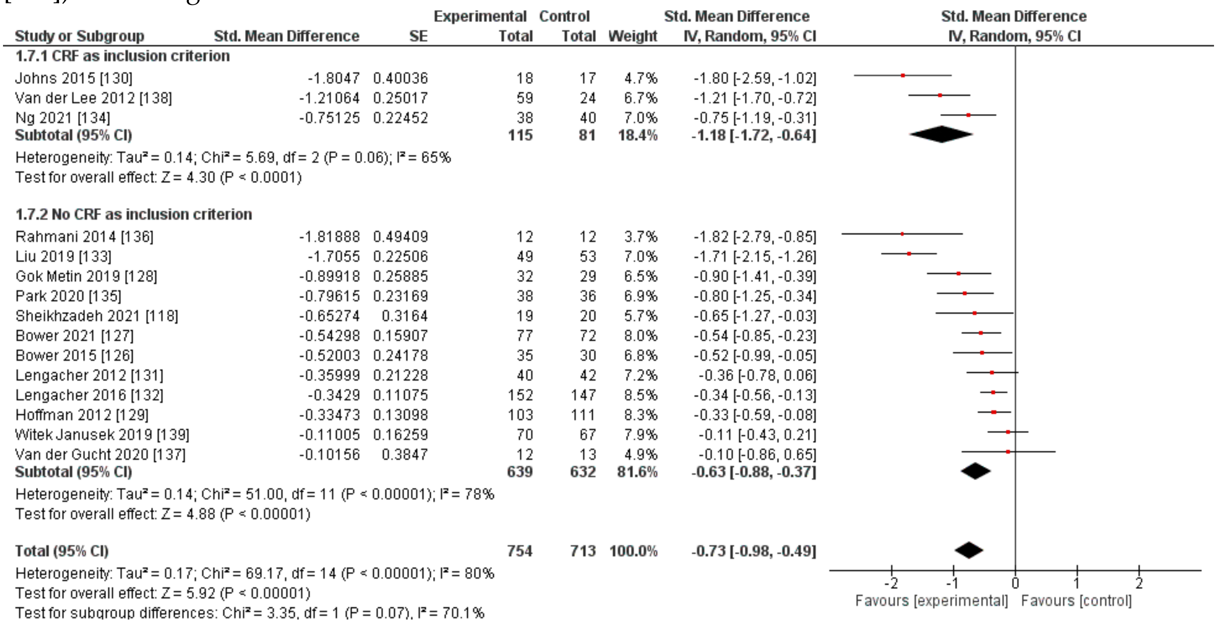

Figure S1.3.3. Comparison of mindfulness-based interventions regarding whether a specific level of CRF was required as an inclusion criterion.

Note: CRF = cancer-related fatigue.

Study or Subgroup	Std. Mean Difference	SE	Experimental Total	Control Total	Weight	Std. Mean Difference IV, Random, 95% CI
**1.16.1 Mean Age<50 years**						
Rahmani 2014 [136]	-1.81888	0.49409	12	12	4.1%	-1.82 [-2.79, -0.85]
Liu 2019 [133]	-1.7055	0.22506	49	53	7.6%	-1.71 [-2.15, -1.26]
Gok Metin 2019 [128]	-0.89918	0.25885	32	29	7.1%	-0.90 [-1.41, -0.39]
Sheikhzadeh 2021 [118]	-0.65274	0.3164	19	20	6.2%	-0.65 [-1.27, -0.03]
Bower 2021 [127]	-0.54298	0.15907	35	30	8.5%	-0.54 [-0.85, -0.23]
Bower 2015 [126]	-0.52003	0.24178	77	72	7.3%	-0.52 [-0.99, -0.05]
Hoffman 2012 [129]	-0.33473	0.13098	103	111	8.9%	-0.33 [-0.59, -0.08]
Van der Gucht 2020 [137]	-0.10156	0.3847	12	13	5.3%	-0.10 [-0.86, 0.65]
**Subtotal (95% CI)**			**339**	**340**	**55.0%**	**-0.78 [-1.16, -0.40]**
Heterogeneity: Tau² = 0.22; Chi² = 37.08, df = 7 (P < 0.00001); I² = 81%						
Test for overall effect: Z = 4.05 (P < 0.0001)						
**1.16.2 Mean Age ≥50 years**						
Johns 2015 [130]	-1.8047	0.40036	18	17	5.1%	-1.80 [-2.59, -1.02]
Van der Lee 2012 [138]	-1.21064	0.25017	59	24	7.2%	-1.21 [-1.70, -0.72]
Park 2020 [135]	-0.79615	0.23169	38	36	7.5%	-0.80 [-1.25, -0.34]
Ng 2021 [134]	-0.75125	0.22452	38	40	7.6%	-0.75 [-1.19, -0.31]
Lengacher 2016 [132]	-0.3429	0.11075	152	147	9.1%	-0.34 [-0.56, -0.13]
Witek Janusek 2019 [139]	-0.11005	0.16259	70	67	8.5%	-0.11 [-0.43, 0.21]
**Subtotal (95% CI)**			**375**	**331**	**45.0%**	**-0.75 [-1.14, -0.36]**
Heterogeneity: Tau² = 0.19; Chi² = 29.31, df = 5 (P < 0.0001); I² = 83%						
Test for overall effect: Z = 3.75 (P = 0.0002)						
**Total (95% CI)**			**714**	**671**	**100.0%**	**-0.76 [-1.02, -0.51]**
Heterogeneity: Tau² = 0.18; Chi² = 68.14, df = 13 (P < 0.00001); I² = 81%						
Test for overall effect: Z = 5.79 (P < 0.00001)						
Test for subgroup differences: Chi² = 0.01, df = 1 (P = 0.91), I² = 0%						

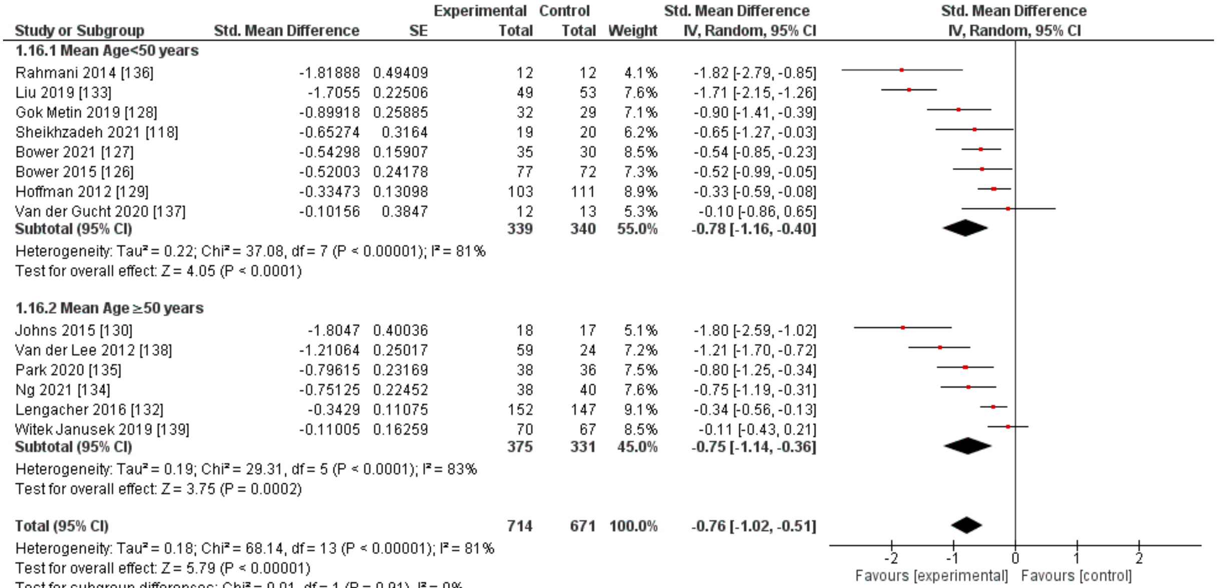

Figure S1.3.4. Comparison of mindfulness-based interventions regarding age of study population (cut-off was oriented towards median (=48.7 years)).

Note: One study (Lengacher (2012) [131]) did not report a mean age.

References

87. Prakash, K.; Saini, S.K.; Pugazhendi, S. Effectiveness of Yoga on Quality of Life of Breast Cancer Patients Undergoing Chemotherapy: A Randomized Clinical Controlled Study. *Indian J. Palliat. Care* **2020**, *26*, 323–331. https://doi.org/10.4103/ijpc.Ijpc_192_19.
93. Vadiraja, S.H.; Rao, M.R.; Nagendra, R.H.; Nagarathna, R.; Rekha, M.; Vanitha, N.; Gopinath, S.K.; Srinath, B.; Vishweshwara, M.; Madhavi, Y.; et al. Effects of yoga on symptom management in breast cancer patients: A randomized controlled trial. *Int. J. Yoga Ther.* **2009**, *2*, 73–79. https://doi.org/10.4103/0973-6131.60048.
104. Gaston-Johansson, F.; Fall-Dickson, J.M.; Nanda, J.; Ohly, K.V.; Stillman, S.; Krumm, S.; Kennedy, M.J. The effectiveness of the comprehensive coping strategy program on clinical outcomes in breast cancer autologous bone marrow transplantation. *Cancer Nurs.* **2000**, *23*, 277–285. https://doi.org/10.1097/00002820-200008000-00004.
118. Sheikhzadeh, M.; Zanjani, Z.; Baari, A. Efficacy of Mindfulness-Based Cognitive Therapy and Cognitive Behavioral Therapy for Anxiety, Depression, and Fatigue in Cancer Patients: A Randomized Clinical Trial. *Iran. J. Psychiatry* **2021**, *16*, 271–280. https://doi.org/10.18502/ijps.v16i3.6252.
131. Lengacher, C.A.; Reich, R.R.; Post-White, J.; Moscoso, M.; Shelton, M.M.; Barta, M.; Le, N.; Budhrani, P. Mindfulness based stress reduction in post-treatment breast cancer patients: An examination of symptoms and symptom clusters. *J. Behav. Med.* **2012**, *35*, 86–94. https://doi.org/10.1007/s10865-011-9346-4.
134. Ng, D.L.; Gan, G.G.; Anuar, N.A.; Tung, Y.Z.; Lai, N.Z.; Tan, Y.W.; Said, S.N.M.; Madihie, A.; Chai, C.S.; Tan, S.B. The effect of a single session of 30-min mindful breathing in reducing fatigue among patients with haematological cancer—A randomised controlled trial. *BMC Palliat. Care* **2021**, *20*, 160. https://doi.org/10.1186/s12904-021-00855-7.
135. Park, S.; Sato, Y.; Takita, Y.; Tamura, N.; Ninomiya, A.; Kosugi, T.; Sado, M.; Nakagawa, A.; Takahashi, M.; Hayashida, T.; et al. Mindfulness-Based Cognitive Therapy for Psychological Distress, Fear of Cancer Recurrence, Fatigue, Spiritual Well-Being, and Quality of Life in Patients with Breast Cancer-A Randomized Controlled Trial. *J. Pain Symptom Manag.* **2020**, *60*, 381–389. https://doi.org/10.1016/j.jpainsymman.2020.02.017.
