# Supplementary material for: Meta-Analysis of Randomized Controlled Trials on Yoga, Psychosocial, and Mindfulness-Based Interventions for Cancer-Related Fatigue: What Intervention Characteristics Are Related to Higher Efficacy?
Source: Cancers (Basel). 2022 Apr 15;14(8):2016. doi: 10.3390/cancers14082016 (PMC9032769; doi:10.3390/cancers14082016)
Supplement: Supplementary file 1 [file cancers-14-02016-s001.zip › Supplementary Section S2_Risk of bias assessment_Proof.pdf]

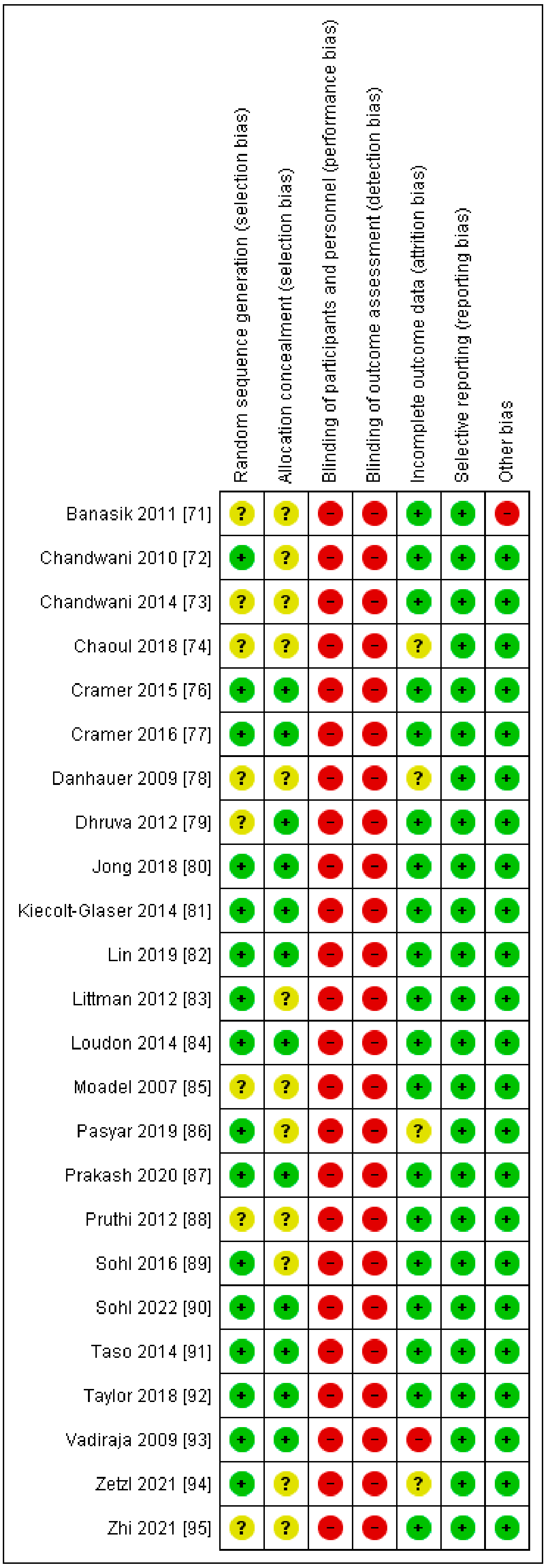


**Figure S2.1.** Risk of bias summary: review authors' judgements about each risk of bias item for each included study with a yoga intervention.

*Note:* green=low risk (requirements adequately fulfilled); yellow=unclear (information insufficient for judgement); red=high risk (requirements not adequately fulfilled).

| | Random sequence generation (selection bias) | Allocation concealment (selection bias) | Blinding of participants and personnel (performance bias) | Blinding of outcome assessment (detection bias) | Incomplete outcome data (attrition bias) | Selective reporting (reporting bias) | Other bias |
|---|---|---|---|---|---|---|---|
| Andersen 2004 [96] | + | ? | − | − | + | + | + |
| Armes 2007 [97] | + | + | − | − | + | + | + |
| Arving 2007 EG1 [98] | ? | ? | − | − | + | + | + |
| Arving 2007 EG2 [98] | ? | ? | − | − | + | + | + |
| Bourmaud 2017 [99] | + | + | − | − | + | + | + |
| Cohen & Fried 2007 [100] | ? | ? | − | − | + | + | + |
| Courtier 2022 [101] | + | ? | − | − | + | + | + |
| Dolbeault 2009 [102] | ? | + | − | − | + | + | + |
| Fukui 2000 [103] | + | ? | − | − | + | + | + |
| Gaston-Johansson 2000 [104] | ? | ? | − | − | ? | + | + |
| Godino 2006 [105] | ? | ? | − | − | − | + | + |
| Goedendorp 2010 [106] | + | + | − | − | + | + | + |
| Gregoire 2020 [107] | + | ? | − | − | + | + | + |
| Kim 2018 [108] | + | ? | − | − | + | + | + |
| Montgomery 2014 [109] | + | ? | − | − | + | + | + |
| O'Brien 2014 [110] | + | + | − | − | + | + | + |
| Peng 2019 [111] | ? | ? | − | − | + | + | + |
| Purcell 2011 [112] | + | + | − | − | + | + | + |
| Ream 2006 [113] | + | ? | − | − | + | + | + |
| Reif 2013 [114] | + | + | − | − | + | + | + |
| Sadeghi 2016 [115] | ? | ? | − | − | + | + | + |
| Salvetti 2021 [116] | − | + | − | − | + | + | + |
| Schjolberg 2014 [117] | ? | ? | − | − | − | + | + |
| Sheikhzadeh 2021 [118] | + | + | − | − | + | + | + |
| Tu 2021 [119] | + | ? | − | − | ? | + | + |
| Van der Meulen 2014 [120] | + | ? | − | − | + | + | + |
| Vargas 2014 [121] | ? | + | − | − | + | + | + |
| Xian 2021 [122] | + | + | − | − | + | + | + |
| Yates 2005 [123] | + | + | − | − | + | + | + |
| Yuen 2006 [124] | ? | ? | − | − | + | + | + |
| Zhang 2020 [125] | ? | ? | − | − | + | + | + |

**Figure S2.2.** Risk of bias summary: review authors' judgements about each risk of bias item for each included study with a psychosocial intervention.

*Note:* green=low risk (requirements adequately fulfilled); yellow=unclear (information insufficient for judgement); red=high risk (requirements not adequately fulfilled).

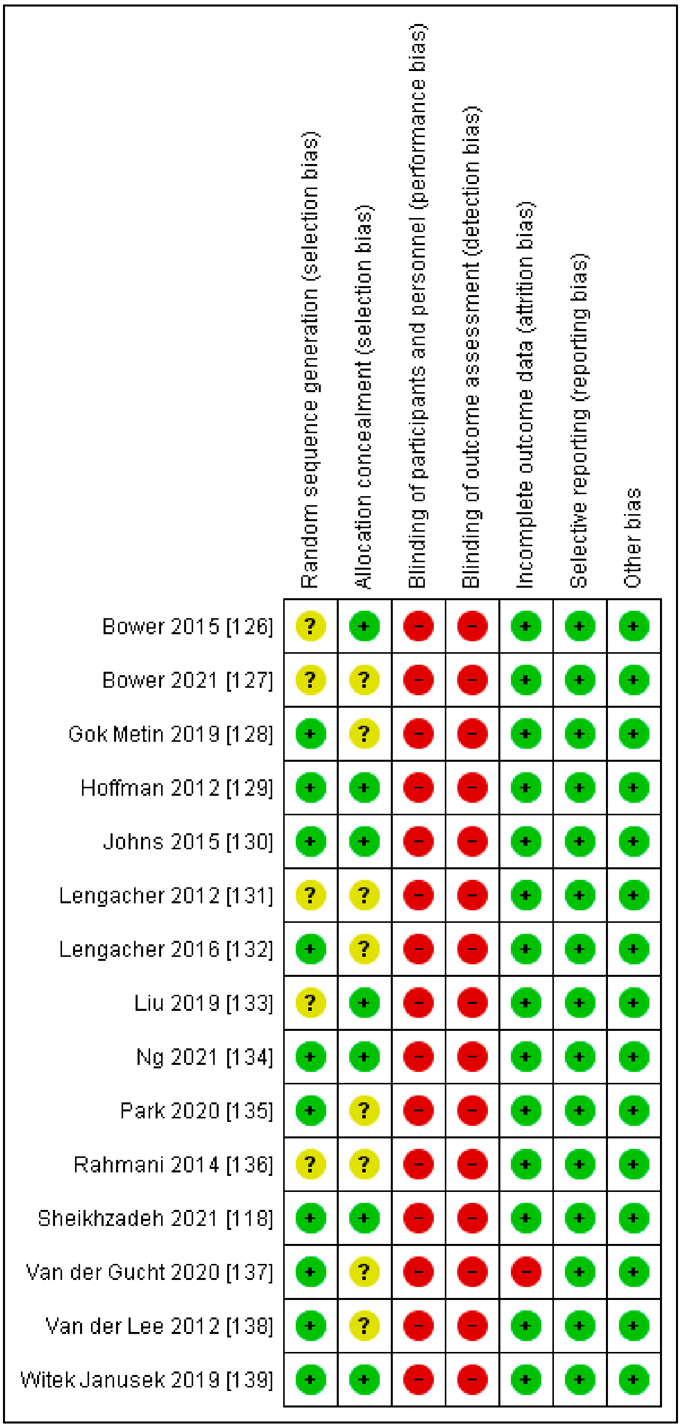


**Figure S2.3.** Risk of bias summary: review authors' judgements about each risk of bias item for each included study with a mindfulness-based intervention.

*Note:* green=low risk (requirements adequately fulfilled); yellow=unclear (information insufficient for judgement); red=high risk (requirements not adequately fulfilled).
