# Supplementary material for: Meta-Analysis of Randomized Controlled Trials on Yoga, Psychosocial, and Mindfulness-Based Interventions for Cancer-Related Fatigue: What Intervention Characteristics Are Related to Higher Efficacy?
Source: Cancers (Basel). 2022 Apr 15;14(8):2016. doi: 10.3390/cancers14082016 (PMC9032769; doi:10.3390/cancers14082016)
Supplement: Supplementary file 1 [file cancers-14-02016-s001.zip › Supplementary Section S3_prediction intervals_Proof.pdf]

Figure S3. Prediction intervals for true effects with a 95% confidence interval of (a) yoga interventions, (b) psychosocial interventions, and (c) mindfulness-based interventions.
Note: The straight line represents the mean effect size with a 95% confidence interval.

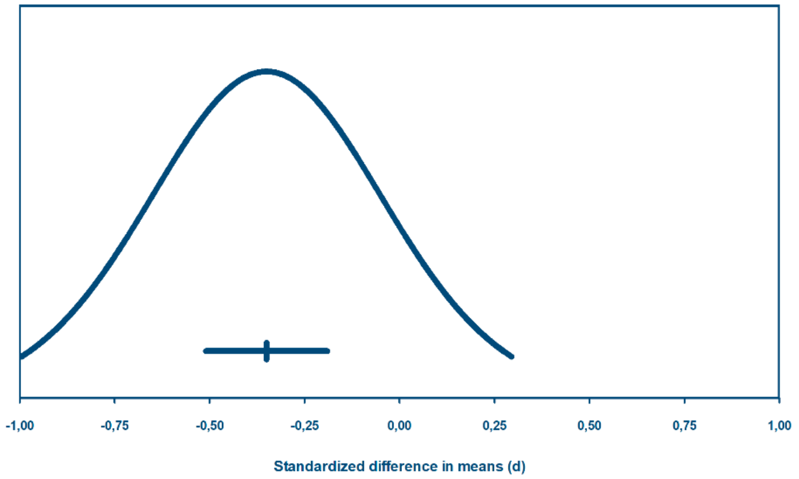

(a)

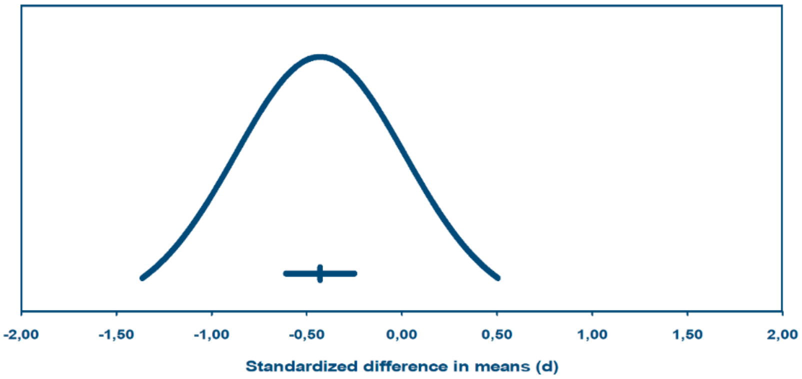

(b)

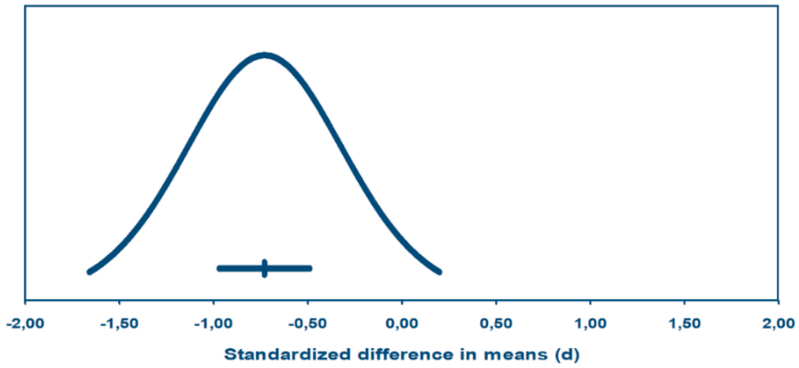

(c)
