# Supplementary material for: Meta-Analysis of Randomized Controlled Trials on Yoga, Psychosocial, and Mindfulness-Based Interventions for Cancer-Related Fatigue: What Intervention Characteristics Are Related to Higher Efficacy?
Source: Cancers (Basel). 2022 Apr 15;14(8):2016. doi: 10.3390/cancers14082016 (PMC9032769; doi:10.3390/cancers14082016)
Supplement: Supplementary file 1 [file cancers-14-02016-s001.zip › Supplementary Section S4_Intercorrelations_Proof.pdf]

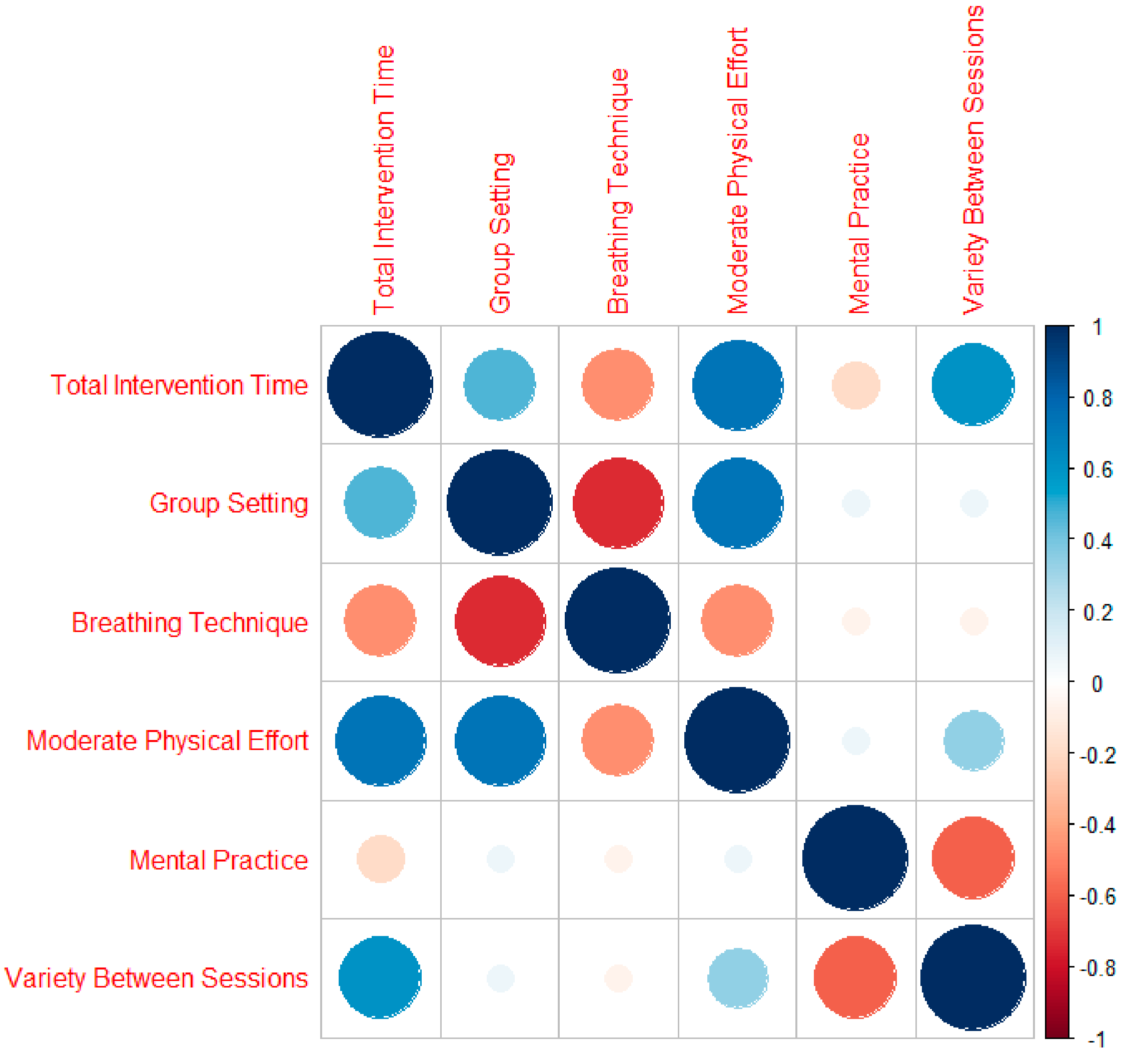

Figure S4. 1. Correlations between characteristics of yoga interventions. *Note:* Correlations base on Phi coefficients or, when *total intervention time* included, point-biserial correlation coefficients; all variables apart from *total intervention time* were coded with 0/1.

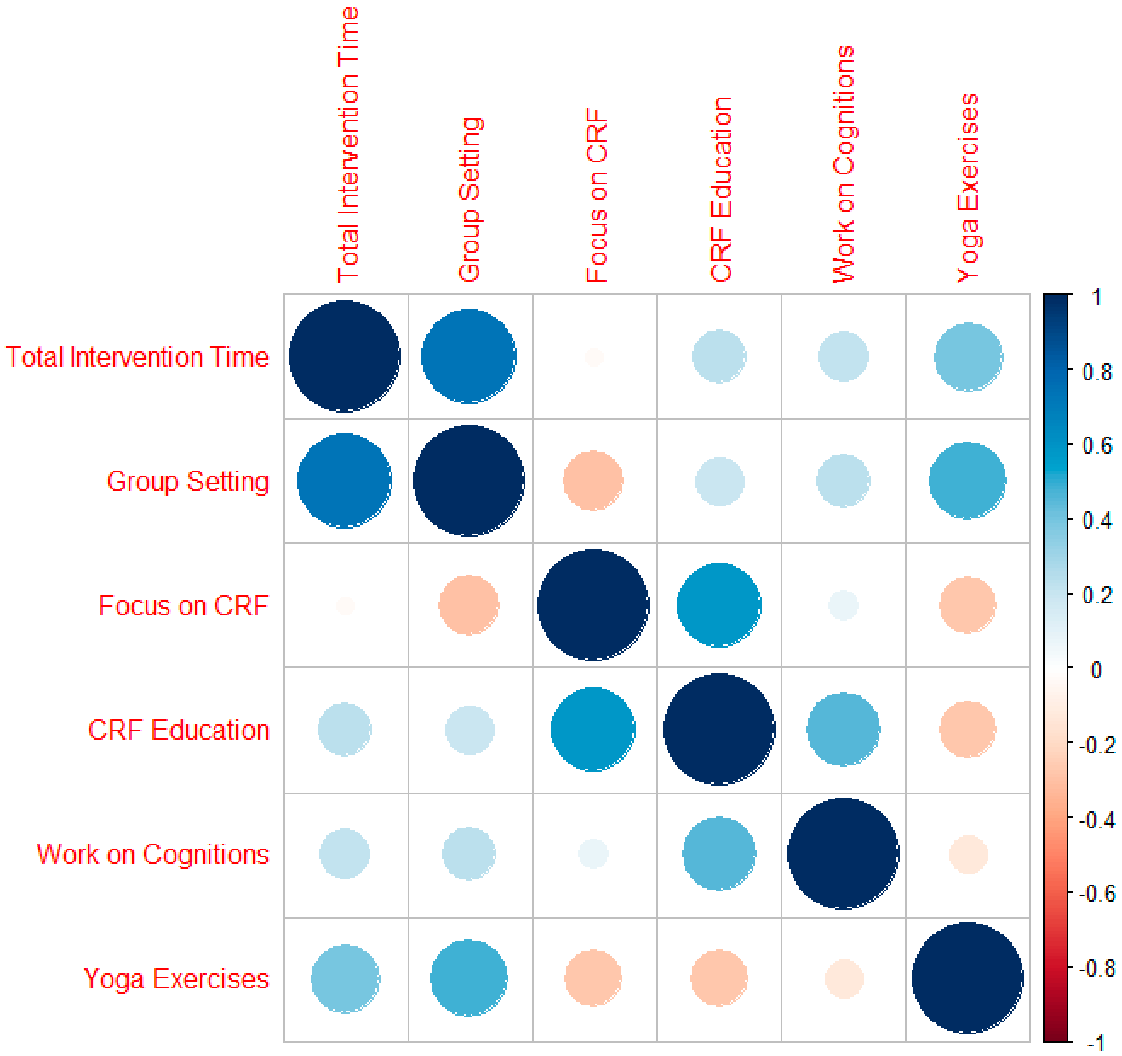

Figure S4. 2. Correlations between characteristics of psychosocial interventions.*Note*: CRF = cancer-related fatigue; correlations base on Phi coefficients or, when *total intervention time* included, point-biserial correlation coefficients; all variables apart from *total intervention time* were coded with 0/1.

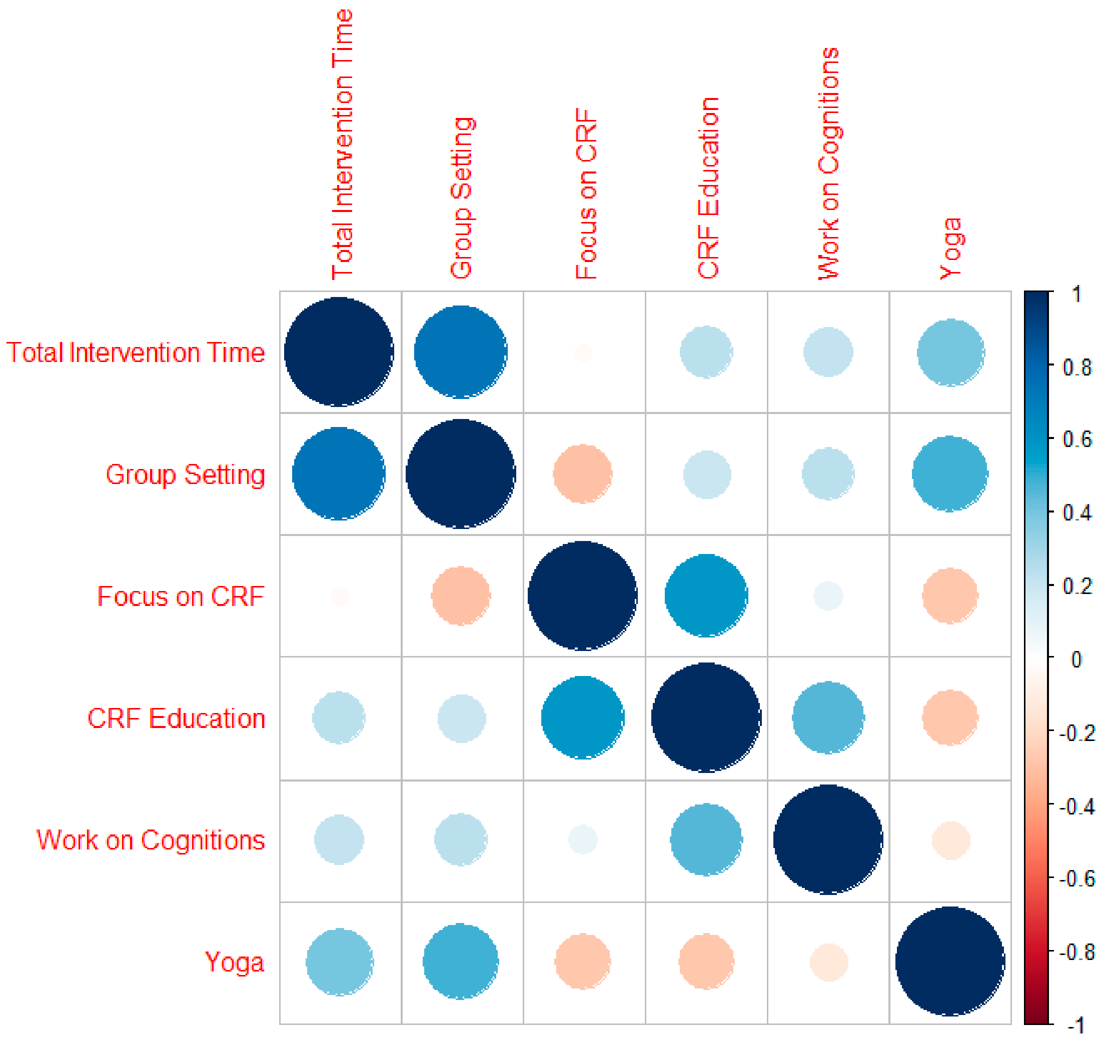

Figure S4. 3. Correlations between characteristics of mindfulness-based interventions. *Note*: CRF = cancer-related fatigue; correlations base on Phi coefficients or, when *total intervention time* included, point-biserial correlation coefficients; all variables apart from *total intervention time* were coded with 0/1.
