# Supplementary material for: Meta-Analysis of Randomized Controlled Trials on Yoga, Psychosocial, and Mindfulness-Based Interventions for Cancer-Related Fatigue: What Intervention Characteristics Are Related to Higher Efficacy?
Source: Cancers (Basel). 2022 Apr 15;14(8):2016. doi: 10.3390/cancers14082016 (PMC9032769; doi:10.3390/cancers14082016)
Supplement: Supplementary file 1 [file cancers-14-02016-s001.zip › Supplementary Section S5_funnel plots_proof.pdf]

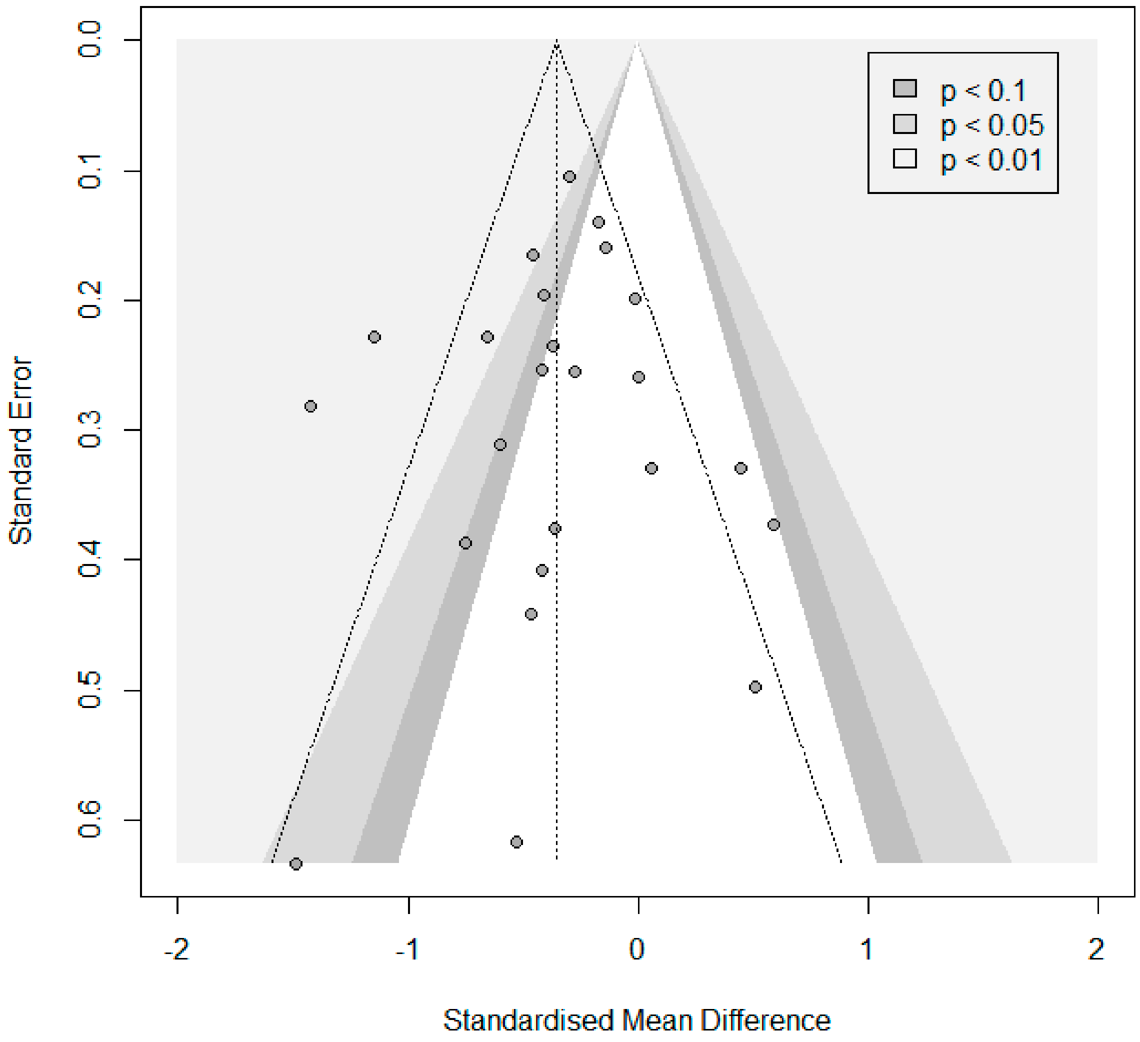

Figure S5. 1. Funnel plot of standard error by standard mean differences of yoga interventions. *Note:* The dashed lines indicate the symmetric distribution of effects expected in the absence of publication bias. The gray shaded areas show how significance levels are related to changing effects and standard errors.

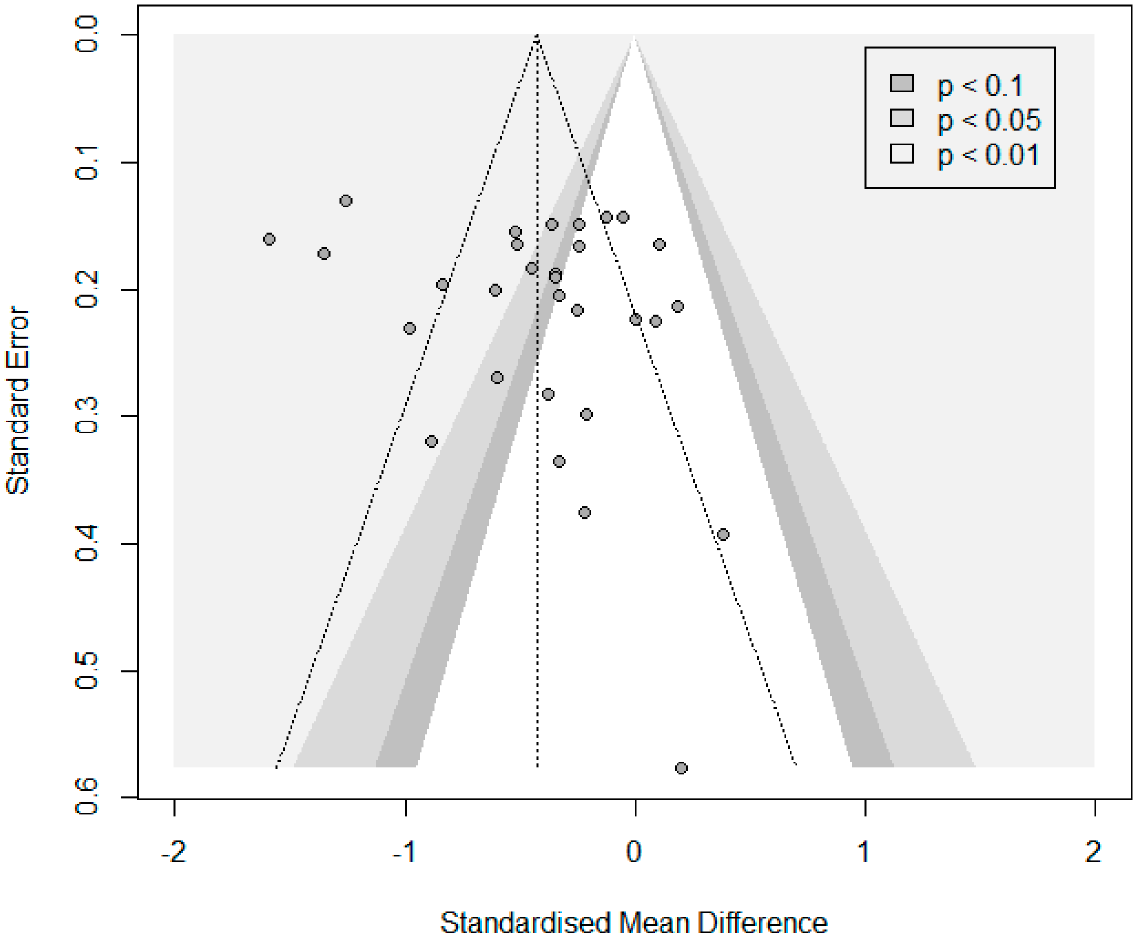

Figure S5. 2. Funnel plot of standard error by standard mean differences of psychosocial interventions. *Note:* The dashed lines indicate the symmetric distribution of effects expected in the absence of publication bias. The gray shaded areas show how significance levels are related to changing effects and standard errors.

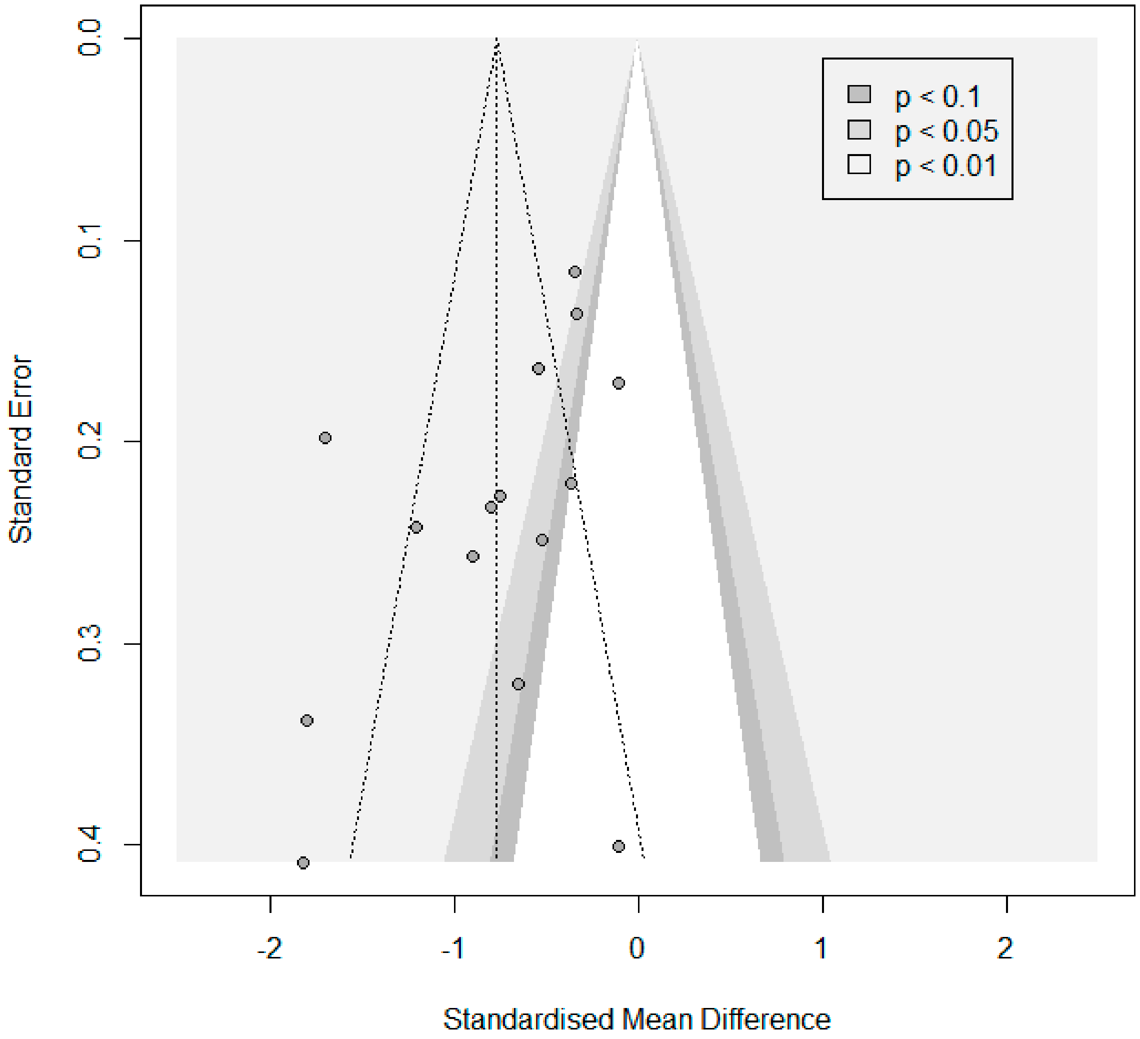

Figure S5. 3. Funnel plot of standard error by standard mean differences of mindfulness-based interventions. *Note:* The dashed lines indicate the symmetric distribution of effects expected in the absence of publication bias. The gray shaded areas show how significance levels are related to changing effects and standard errors.
