# Supplementary material for: Meta-Analysis of Randomized Controlled Trials on Yoga, Psychosocial, and Mindfulness-Based Interventions for Cancer-Related Fatigue: What Intervention Characteristics Are Related to Higher Efficacy?
Source: Cancers (Basel). 2022 Apr 15;14(8):2016. doi: 10.3390/cancers14082016 (PMC9032769; doi:10.3390/cancers14082016)
Supplement: Supplementary file 1 [file cancers-14-02016-s001.zip › Supplementary Section S6_sensitivity analyses_Proof.pdf]

Section S6.1.Yoga Interventions

Study or Subgroup	Std. Mean Difference	SE	Experimental Total	Control Total	Weight	Std. Mean Difference IV, Random, 95% CI
**1.28.1 Studies without high risk of bias**						
Taso 2014 [91]	-1.42798	0.28169	30	30	2.2%	-1.43 [-1.98, -0.88]
Prakash 2020 [87]	-1.14817	0.22846	41	42	2.6%	-1.15 [-1.60, -0.70]
Danhauer 2009 [78]	-0.7546	0.38708	13	14	1.5%	-0.75 [-1.51, 0.00]
Cramer 2015 [76]	-0.5981	0.31099	19	21	1.9%	-0.60 [-1.21, 0.01]
Sohl 2016 [89]	-0.53064	0.61632	6	5	0.7%	-0.53 [-1.74, 0.68]
Taylor 2018 [92]	-0.46158	0.44164	9	11	1.2%	-0.46 [-1.33, 0.40]
Zetzl 2021 [94]	-0.45708	0.16547	69	67	3.2%	-0.46 [-0.78, -0.13]
Chandwani 2010 [72]	-0.41897	0.25378	28	30	2.4%	-0.42 [-0.92, 0.08]
Loudon 2014 [84]	-0.41783	0.4075	12	11	1.4%	-0.42 [-1.22, 0.38]
Chandwani 2014 [73]	-0.40692	0.19551	49	48	2.9%	-0.41 [-0.79, -0.02]
Jong 2018 [80]	-0.37292	0.23591	39	29	2.5%	-0.37 [-0.84, 0.09]
Pasyar 2019 [86]	-0.36362	0.37561	12	15	1.6%	-0.36 [-1.10, 0.37]
Lin 2019 [82]	-0.29831	0.10553	168	160	3.8%	-0.30 [-0.51, -0.09]
Littman 2012 [83]	-0.27384	0.2543	30	27	2.4%	-0.27 [-0.77, 0.22]
Kiecolt-Glaser 2014 [81]	-0.16974	0.13967	96	90	3.4%	-0.17 [-0.44, 0.10]
Chaoul 2018 [74]	-0.1421	0.16006	64	79	3.2%	-0.14 [-0.46, 0.17]
Moadel 2007 [85]	-0.01372	0.19949	73	33	2.9%	-0.01 [-0.40, 0.38]
Cramer 2016 [77]	0	0.2595	27	27	2.3%	0.00 [-0.51, 0.51]
Sohl 2022 [90]	0.05467	0.32878	18	16	1.8%	0.05 [-0.59, 0.70]
Zhi 2021 [95]	0.44306	0.32964	16	19	1.8%	0.44 [-0.20, 1.09]
Dhruva 2012 [79]	0.51035	0.49713	8	8	1.1%	0.51 [-0.46, 1.48]
Pruthi 2012 [88]	0.59053	0.37298	14	14	1.6%	0.59 [-0.14, 1.32]
**Subtotal (95% CI)**			**841**	**796**	**48.3%**	**-0.32 [-0.49, -0.15]**
Heterogeneity: Tau² = 0.08; Chi² = 53.79, df = 21 (P = 0.0001); I² = 61%						
Test for overall effect: Z = 3.76 (P = 0.0002)						
**1.28.2 All studies**						
Banasik 2011 [71]	-1.48664	0.63309	7	7	0.7%	-1.49 [-2.73, -0.25]
Taso 2014 [91]	-1.42798	0.28169	30	30	2.2%	-1.43 [-1.98, -0.88]
Prakash 2020 [87]	-1.14817	0.22846	41	42	2.6%	-1.15 [-1.60, -0.70]
Danhauer 2009 [78]	-0.7546	0.38708	13	14	1.5%	-0.75 [-1.51, 0.00]
Vadiraja 2009 [93]	-0.65541	0.22836	42	33	2.6%	-0.66 [-1.10, -0.21]
Cramer 2015 [76]	-0.5981	0.31099	19	21	1.9%	-0.60 [-1.21, 0.01]
Sohl 2016 [89]	-0.53064	0.61632	6	5	0.7%	-0.53 [-1.74, 0.68]
Taylor 2018 [92]	-0.46158	0.44164	9	11	1.2%	-0.46 [-1.33, 0.40]
Zetzl 2021 [94]	-0.45708	0.16547	69	67	3.2%	-0.46 [-0.78, -0.13]
Chandwani 2010 [72]	-0.41897	0.25378	28	30	2.4%	-0.42 [-0.92, 0.08]
Loudon 2014 [84]	-0.41783	0.4075	12	11	1.4%	-0.42 [-1.22, 0.38]
Chandwani 2014 [73]	-0.40692	0.19551	49	48	2.9%	-0.41 [-0.79, -0.02]
Jong 2018 [80]	-0.37292	0.23591	39	29	2.5%	-0.37 [-0.84, 0.09]
Pasyar 2019 [86]	-0.36362	0.37561	12	15	1.6%	-0.36 [-1.10, 0.37]
Lin 2019 [82]	-0.29831	0.10553	168	160	3.8%	-0.30 [-0.51, -0.09]
Littman 2012 [83]	-0.27384	0.2543	30	27	2.4%	-0.27 [-0.77, 0.22]
Kiecolt-Glaser 2014 [81]	-0.16974	0.13967	96	90	3.4%	-0.17 [-0.44, 0.10]
Chaoul 2018 [74]	-0.1421	0.16006	64	79	3.2%	-0.14 [-0.46, 0.17]
Moadel 2007 [85]	-0.01372	0.19949	73	33	2.9%	-0.01 [-0.40, 0.38]
Cramer 2016 [77]	0	0.2595	27	27	2.3%	0.00 [-0.51, 0.51]
Sohl 2022 [90]	0.05467	0.32878	18	16	1.8%	0.05 [-0.59, 0.70]
Zhi 2021 [95]	0.44306	0.32964	16	19	1.8%	0.44 [-0.20, 1.09]
Dhruva 2012 [79]	0.51035	0.49713	8	8	1.1%	0.51 [-0.46, 1.48]
Pruthi 2012 [88]	0.59053	0.37298	14	14	1.6%	0.59 [-0.14, 1.32]
**Subtotal (95% CI)**			**890**	**836**	**51.7%**	**-0.35 [-0.52, -0.19]**
Heterogeneity: Tau² = 0.09; Chi² = 59.25, df = 23 (P < 0.0001); I² = 61%						
Test for overall effect: Z = 4.23 (P < 0.0001)						
**Total (95% CI)**			**1731**	**1632**	**100.0%**	**-0.34 [-0.45, -0.22]**
Heterogeneity: Tau² = 0.08; Chi² = 113.14, df = 45 (P < 0.00001); I² = 60%						
Test for overall effect: Z = 5.76 (P < 0.00001)						
Test for subgroup differences: Chi² = 0.08, df = 1 (P = 0.78), I² = 0%						

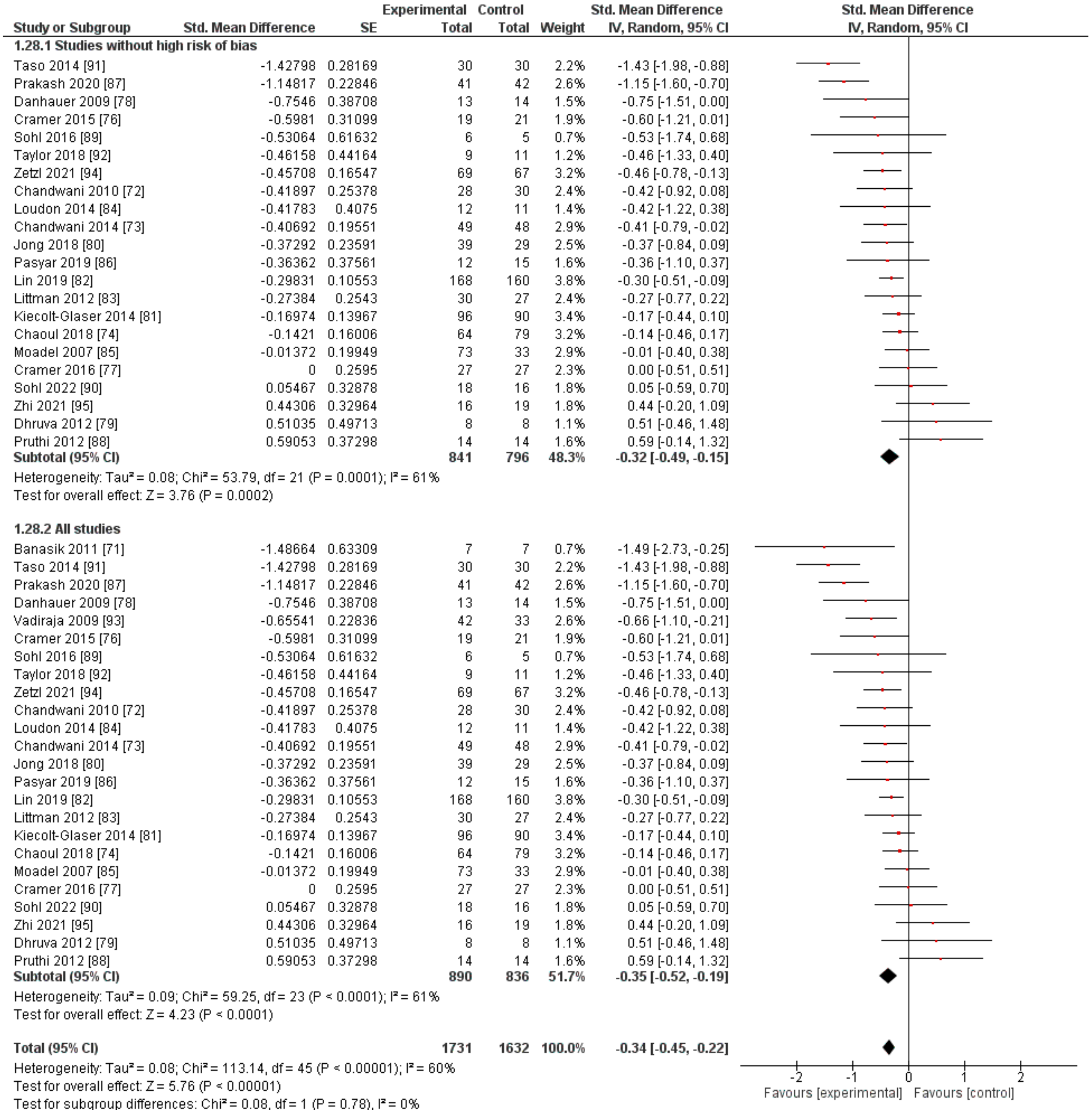

Figure S6.1.1. Sensitivity analysis for the comparison of the effect of yoga interventions without high risk of bias vs. all studies.
Note: High risk of bias was defined as at least one assessed high risk besides blinding of participants and personnel as well as blinding of outcome assessment.

Study or Subgroup	Std. Mean Difference	SE	Experimental Total	Control Total	Weight	Std. Mean Difference IV, Random, 95% CI
**1.26.1 Studies with n ≥ 25 per intervention arm**						
Taso 2014 [91]	-1.42798	0.28169	30	30	2.4%	-1.43 [-1.98, -0.88]
Prakash 2020 [87]	-1.14817	0.22846	41	42	2.9%	-1.15 [-1.60, -0.70]
Vadiraja 2009 [93]	-0.65541	0.22836	42	33	2.9%	-0.66 [-1.10, -0.21]
Zetzl 2021 [94]	-0.45708	0.16547	69	67	3.6%	-0.46 [-0.78, -0.13]
Chandwani 2010 [72]	-0.41897	0.25378	28	30	2.7%	-0.42 [-0.92, 0.08]
Chandwani 2014 [73]	-0.40692	0.19551	49	48	3.3%	-0.41 [-0.79, -0.02]
Jong 2018 [80]	-0.37292	0.23591	39	29	2.9%	-0.37 [-0.84, 0.09]
Lin 2019 [82]	-0.29831	0.10553	168	160	4.3%	-0.30 [-0.51, -0.09]
Littman 2012 [83]	-0.27384	0.2543	30	27	2.7%	-0.27 [-0.77, 0.22]
Kiecolt-Glaser 2014 [81]	-0.16974	0.13967	96	90	3.9%	-0.17 [-0.44, 0.10]
Chaoul 2018 [74]	-0.1421	0.16006	64	79	3.7%	-0.14 [-0.46, 0.17]
Moadel 2007 [85]	-0.01372	0.19949	73	33	3.3%	-0.01 [-0.40, 0.38]
Cramer 2016 [77]	0	0.2595	27	27	2.6%	0.00 [-0.51, 0.51]
**Subtotal (95% CI)**			**756**	**695**	**41.4%**	**-0.42 [-0.60, -0.23]**
Heterogeneity: Tau² = 0.08; Chi² = 37.51, df = 12 (P = 0.0002); I² = 68%						
Test for overall effect: Z = 4.38 (P < 0.0001)						
**1.26.2 All studies**						
Banasik 2011 [71]	-1.48664	0.63309	7	7	0.8%	-1.49 [-2.73, -0.25]
Taso 2014 [91]	-1.42798	0.28169	30	30	2.4%	-1.43 [-1.98, -0.88]
Prakash 2020 [87]	-1.14817	0.22846	41	42	2.9%	-1.15 [-1.60, -0.70]
Danhauer 2009 [78]	-0.7546	0.38708	13	14	1.7%	-0.75 [-1.51, 0.00]
Vadiraja 2009 [93]	-0.65541	0.22836	42	33	2.9%	-0.66 [-1.10, -0.21]
Cramer 2015 [76]	-0.5981	0.31099	19	21	2.2%	-0.60 [-1.21, 0.01]
Sohl 2016 [89]	-0.53064	0.61632	6	5	0.8%	-0.53 [-1.74, 0.68]
Taylor 2018 [92]	-0.46158	0.44164	9	11	1.4%	-0.46 [-1.33, 0.40]
Zetzl 2021 [94]	-0.45708	0.16547	69	67	3.6%	-0.46 [-0.78, -0.13]
Chandwani 2010 [72]	-0.41897	0.25378	28	30	2.7%	-0.42 [-0.92, 0.08]
Loudon 2014 [84]	-0.41783	0.4075	12	11	1.6%	-0.42 [-1.22, 0.38]
Chandwani 2014 [73]	-0.40692	0.19551	49	48	3.3%	-0.41 [-0.79, -0.02]
Jong 2018 [80]	-0.37292	0.23591	39	29	2.9%	-0.37 [-0.84, 0.09]
Pasyar 2019 [86]	-0.36362	0.37561	12	15	1.7%	-0.36 [-1.10, 0.37]
Lin 2019 [82]	-0.29831	0.10553	168	160	4.3%	-0.30 [-0.51, -0.09]
Littman 2012 [83]	-0.27384	0.2543	30	27	2.7%	-0.27 [-0.77, 0.22]
Kiecolt-Glaser 2014 [81]	-0.16974	0.13967	96	90	3.9%	-0.17 [-0.44, 0.10]
Chaoul 2018 [74]	-0.1421	0.16006	64	79	3.7%	-0.14 [-0.46, 0.17]
Moadel 2007 [85]	-0.01372	0.19949	73	33	3.3%	-0.01 [-0.40, 0.38]
Cramer 2016 [77]	0	0.2595	27	27	2.6%	0.00 [-0.51, 0.51]
Sohl 2022 [90]	0.05467	0.32878	18	16	2.1%	0.05 [-0.59, 0.70]
Zhi 2021 [95]	0.44306	0.32964	16	19	2.0%	0.44 [-0.20, 1.09]
Dhruva 2012 [79]	0.51035	0.49713	8	8	1.2%	0.51 [-0.46, 1.48]
Pruthi 2012 [88]	0.59053	0.37298	14	14	1.8%	0.59 [-0.14, 1.32]
**Subtotal (95% CI)**			**890**	**836**	**58.6%**	**-0.35 [-0.52, -0.19]**
Heterogeneity: Tau² = 0.09; Chi² = 59.25, df = 23 (P < 0.0001); I² = 61%						
Test for overall effect: Z = 4.23 (P < 0.0001)						
**Total (95% CI)**			**1646**	**1531**	**100.0%**	**-0.38 [-0.50, -0.26]**
Heterogeneity: Tau² = 0.08; Chi² = 96.92, df = 36 (P < 0.00001); I² = 63%						
Test for overall effect: Z = 6.17 (P < 0.00001)						
Test for subgroup differences: Chi² = 0.25, df = 1 (P = 0.62), I² = 0%						

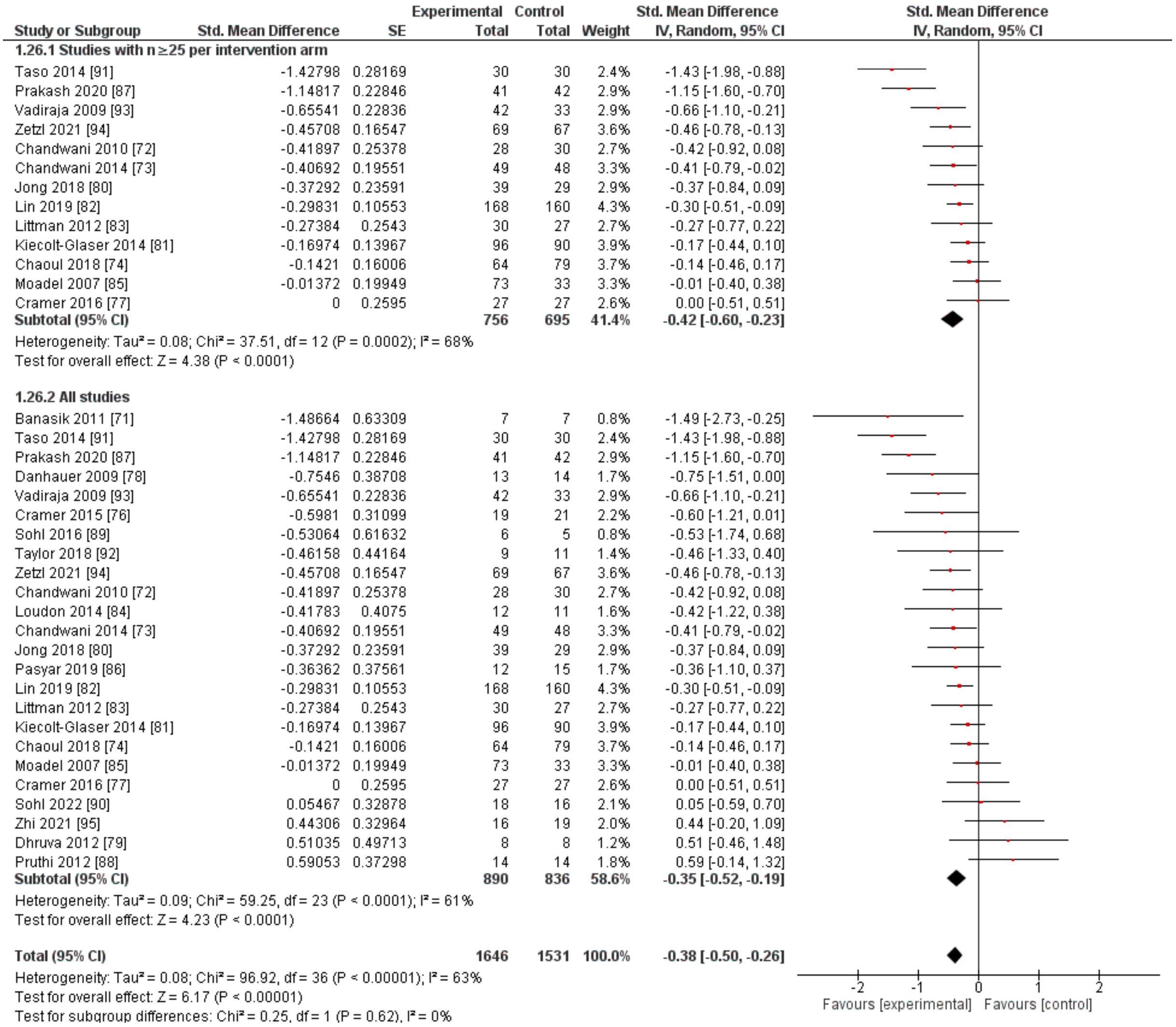

Figure S6.1.2. Sensitivity analysis for the comparison of the effect of yoga interventions with more than 25 patients per intervention arm vs. all studies.

Study or Subgroup	Std. Mean Difference	SE	Experimental Total	Control Total	Weight	Std. Mean Difference IV, Random, 95% CI
**1.27.1 Studies without specific study population**						
Banasik 2011 [71]	-1.48664	0.63309	7	7	0.8%	-1.49 [-2.73, -0.25]
Taso 2014 [91]	-1.42798	0.28169	30	30	2.2%	-1.43 [-1.98, -0.88]
Prakash 2020 [87]	-1.14817	0.22846	41	42	2.6%	-1.15 [-1.60, -0.70]
Danhauer 2009 [78]	-0.7546	0.38708	13	14	1.6%	-0.75 [-1.51, 0.00]
Vadiraja 2009 [93]	-0.65541	0.22836	42	33	2.6%	-0.66 [-1.10, -0.21]
Cramer 2015 [76]	-0.5981	0.31099	19	21	2.0%	-0.60 [-1.21, 0.01]
Sohl 2016 [89]	-0.53064	0.61632	6	5	0.8%	-0.53 [-1.74, 0.68]
Taylor 2018 [92]	-0.46158	0.44164	9	11	1.3%	-0.46 [-1.33, 0.40]
Zetzl 2021 [94]	-0.45708	0.16547	69	67	3.2%	-0.46 [-0.78, -0.13]
Chandwani 2010 [72]	-0.41897	0.25378	28	30	2.4%	-0.42 [-0.92, 0.08]
Loudon 2014 [84]	-0.41783	0.4075	12	11	1.5%	-0.42 [-1.22, 0.38]
Chandwani 2014 [73]	-0.40692	0.19551	49	48	2.9%	-0.41 [-0.79, -0.02]
Jong 2018 [80]	-0.37292	0.23591	39	29	2.6%	-0.37 [-0.84, 0.09]
Pasyar 2019 [86]	-0.36362	0.37561	12	15	1.6%	-0.36 [-1.10, 0.37]
Littman 2012 [83]	-0.27384	0.2543	30	27	2.4%	-0.27 [-0.77, 0.22]
Kiecolt-Glaser 2014 [81]	-0.16974	0.13967	96	90	3.5%	-0.17 [-0.44, 0.10]
Chaoul 2018 [74]	-0.1421	0.16006	64	79	3.3%	-0.14 [-0.46, 0.17]
Moadel 2007 [85]	-0.01372	0.19949	73	33	2.9%	-0.01 [-0.40, 0.38]
Cramer 2016 [77]	0	0.2595	27	27	2.4%	0.00 [-0.51, 0.51]
Sohl 2022 [90]	0.05467	0.32878	18	16	1.9%	0.05 [-0.59, 0.70]
Dhruva 2012 [79]	0.51035	0.49713	8	8	1.1%	0.51 [-0.46, 1.48]
Pruthi 2012 [88]	0.59053	0.37298	14	14	1.6%	0.59 [-0.14, 1.32]
**Subtotal (95% CI)**			**706**	**657**	**47.2%**	**-0.39 [-0.57, -0.21]**
Heterogeneity: Tau² = 0.10; Chi² = 53.26, df = 21 (P = 0.0001); I² = 61%						
Test for overall effect: Z = 4.27 (P < 0.0001)						
**1.27.2 All studies**						
Banasik 2011 [71]	-1.48664	0.63309	7	7	0.8%	-1.49 [-2.73, -0.25]
Taso 2014 [91]	-1.42798	0.28169	30	30	2.2%	-1.43 [-1.98, -0.88]
Prakash 2020 [87]	-1.14817	0.22846	41	42	2.6%	-1.15 [-1.60, -0.70]
Danhauer 2009 [78]	-0.7546	0.38708	13	14	1.6%	-0.75 [-1.51, 0.00]
Vadiraja 2009 [93]	-0.65541	0.22836	42	33	2.6%	-0.66 [-1.10, -0.21]
Cramer 2015 [76]	-0.5981	0.31099	19	21	2.0%	-0.60 [-1.21, 0.01]
Sohl 2016 [89]	-0.53064	0.61632	6	5	0.8%	-0.53 [-1.74, 0.68]
Taylor 2018 [92]	-0.46158	0.44164	9	11	1.3%	-0.46 [-1.33, 0.40]
Zetzl 2021 [94]	-0.45708	0.16547	69	67	3.2%	-0.46 [-0.78, -0.13]
Chandwani 2010 [72]	-0.41897	0.25378	28	30	2.4%	-0.42 [-0.92, 0.08]
Loudon 2014 [84]	-0.41783	0.4075	12	11	1.5%	-0.42 [-1.22, 0.38]
Chandwani 2014 [73]	-0.40692	0.19551	49	48	2.9%	-0.41 [-0.79, -0.02]
Jong 2018 [80]	-0.37292	0.23591	39	29	2.6%	-0.37 [-0.84, 0.09]
Pasyar 2019 [86]	-0.36362	0.37561	12	15	1.6%	-0.36 [-1.10, 0.37]
Lin 2019 [82]	-0.29831	0.10553	168	160	3.7%	-0.30 [-0.51, -0.09]
Littman 2012 [83]	-0.27384	0.2543	30	27	2.4%	-0.27 [-0.77, 0.22]
Kiecolt-Glaser 2014 [81]	-0.16974	0.13967	96	90	3.5%	-0.17 [-0.44, 0.10]
Chaoul 2018 [74]	-0.1421	0.16006	64	79	3.3%	-0.14 [-0.46, 0.17]
Moadel 2007 [85]	-0.01372	0.19949	73	33	2.9%	-0.01 [-0.40, 0.38]
Cramer 2016 [77]	0	0.2595	27	27	2.4%	0.00 [-0.51, 0.51]
Sohl 2022 [90]	0.05467	0.32878	18	16	1.9%	0.05 [-0.59, 0.70]
Zhi 2021 [95]	0.44306	0.32964	16	19	1.9%	0.44 [-0.20, 1.09]
Dhruva 2012 [79]	0.51035	0.49713	8	8	1.1%	0.51 [-0.46, 1.48]
Pruthi 2012 [88]	0.59053	0.37298	14	14	1.6%	0.59 [-0.14, 1.32]
**Subtotal (95% CI)**			**890**	**836**	**52.8%**	**-0.35 [-0.52, -0.19]**
Heterogeneity: Tau² = 0.09; Chi² = 59.25, df = 23 (P < 0.0001); I² = 61%						
Test for overall effect: Z = 4.23 (P < 0.0001)						
**Total (95% CI)**			**1596**	**1493**	**100.0%**	**-0.37 [-0.49, -0.25]**
Heterogeneity: Tau² = 0.09; Chi² = 112.68, df = 45 (P < 0.00001); I² = 60%						
Test for overall effect: Z = 6.11 (P < 0.00001)						
Test for subgroup differences: Chi² = 0.09, df = 1 (P = 0.77), I² = 0%						

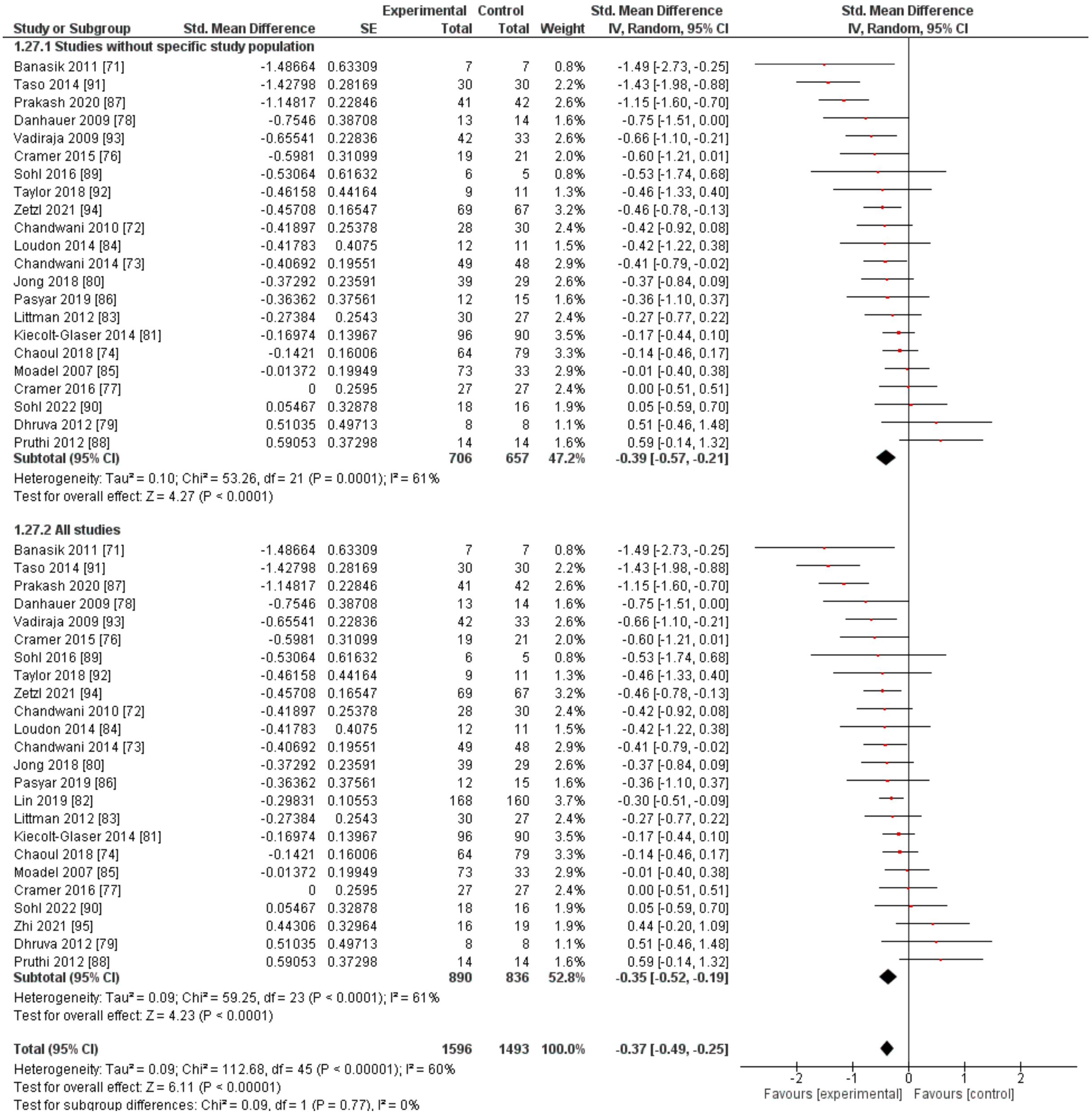

Figure S6.1.3. Sensitivity analysis for the comparison of yoga interventions including study populations without specific physical or mental impairment vs. all studies.
Note: Lin (2019) [82] specifically included study participants with sleep disturbances; Zhi (2021) [95] with chemotherapy-induced peripheral neuropathy.

Study or Subgroup	Std. Mean Difference	SE	Experimental Total	Control Total	Weight	Std. Mean Difference IV, Random, 95% CI
**1.21.1 Wait-List-Control**						
Banasik 2011 [71]	-1.48664	0.63309	7	7	1.4%	-1.49 [-2.73, -0.25]
Danhauer 2009 [78]	-0.7546	0.38708	13	14	2.9%	-0.75 [-1.51, 0.00]
Cramer 2015 [76]	-0.5981	0.31099	19	21	3.8%	-0.60 [-1.21, 0.01]
Taylor 2018 [92]	-0.46158	0.44164	9	11	2.5%	-0.46 [-1.33, 0.40]
Zetzl 2021 [94]	-0.45708	0.16547	69	67	6.1%	-0.46 [-0.78, -0.13]
Loudon 2014 [84]	-0.41783	0.4075	12	11	2.8%	-0.42 [-1.22, 0.38]
Chandwani 2014 [73]	-0.40692	0.19551	49	48	5.6%	-0.41 [-0.79, -0.02]
Jong 2018 [80]	-0.37292	0.23591	42	29	4.9%	-0.37 [-0.84, 0.09]
Chandwani 2010 [72]	-0.29932	0.2526	27	31	4.6%	-0.30 [-0.79, 0.20]
Littman 2012 [83]	-0.27088	0.26096	27	27	4.5%	-0.27 [-0.78, 0.24]
Kiecolt-Glaser 2014 [81]	-0.16974	0.13967	96	90	6.5%	-0.17 [-0.44, 0.10]
Chaoul 2018 [74]	-0.1421	0.16006	64	79	6.2%	-0.14 [-0.46, 0.17]
Moadel 2007 [85]	-0.01366	0.17691	84	44	5.9%	-0.01 [-0.36, 0.33]
Cramer 2016 [77]	0	0.29129	21	22	4.1%	0.00 [-0.57, 0.57]
Zhi 2021 [95]	0.44306	0.32964	16	19	3.6%	0.44 [-0.20, 1.09]
Dhruva 2012 [79]	0.51035	0.49713	8	8	2.1%	0.51 [-0.46, 1.48]
Pruthi 2012 [88]	0.59053	0.37298	14	14	3.1%	0.59 [-0.14, 1.32]
**Subtotal (95% CI)**			**577**	**542**	**70.5%**	**-0.23 [-0.38, -0.07]**
Heterogeneity: Tau² = 0.03; Chi² = 24.66, df = 16 (P = 0.08); I² = 35%						
Test for overall effect: Z = 2.91 (P = 0.004)						
**1.21.2 Standard Care**						
Taso 2014 [91]	-1.42798	0.28169	30	30	4.2%	-1.43 [-1.98, -0.88]
Prakash 2020 [87]	-1.14817	0.22846	41	42	5.0%	-1.15 [-1.60, -0.70]
Vadiraja 2009 [93]	-0.65541	0.22836	42	33	5.0%	-0.66 [-1.10, -0.21]
Pasyar 2019 [86]	-0.36362	0.37561	12	15	3.1%	-0.36 [-1.10, 0.37]
Lin 2019 [82]	-0.29831	0.10553	168	160	7.1%	-0.30 [-0.51, -0.09]
**Subtotal (95% CI)**			**293**	**280**	**24.4%**	**-0.77 [-1.23, -0.31]**
Heterogeneity: Tau² = 0.22; Chi² = 22.71, df = 4 (P = 0.0001); I² = 82%						
Test for overall effect: Z = 3.27 (P = 0.001)						
**1.21.3 Attention Control**						
Sohl 2016 [89]	-0.5308	0.61633	6	5	1.5%	-0.53 [-1.74, 0.68]
Sohl 2022 [90]	0.05467	0.32878	18	16	3.6%	0.05 [-0.59, 0.70]
**Subtotal (95% CI)**			**24**	**21**	**5.1%**	**-0.08 [-0.64, 0.49]**
Heterogeneity: Tau² = 0.00; Chi² = 0.70, df = 1 (P = 0.40); I² = 0%						
Test for overall effect: Z = 0.26 (P = 0.80)						
**Total (95% CI)**			**894**	**843**	**100.0%**	**-0.35 [-0.51, -0.18]**
Heterogeneity: Tau² = 0.09; Chi² = 59.51, df = 23 (P < 0.0001); I² = 61%						
Test for overall effect: Z = 4.17 (P < 0.0001)						
Test for subgroup differences: Chi² = 5.28, df = 2 (P = 0.07), I² = 62.1%						

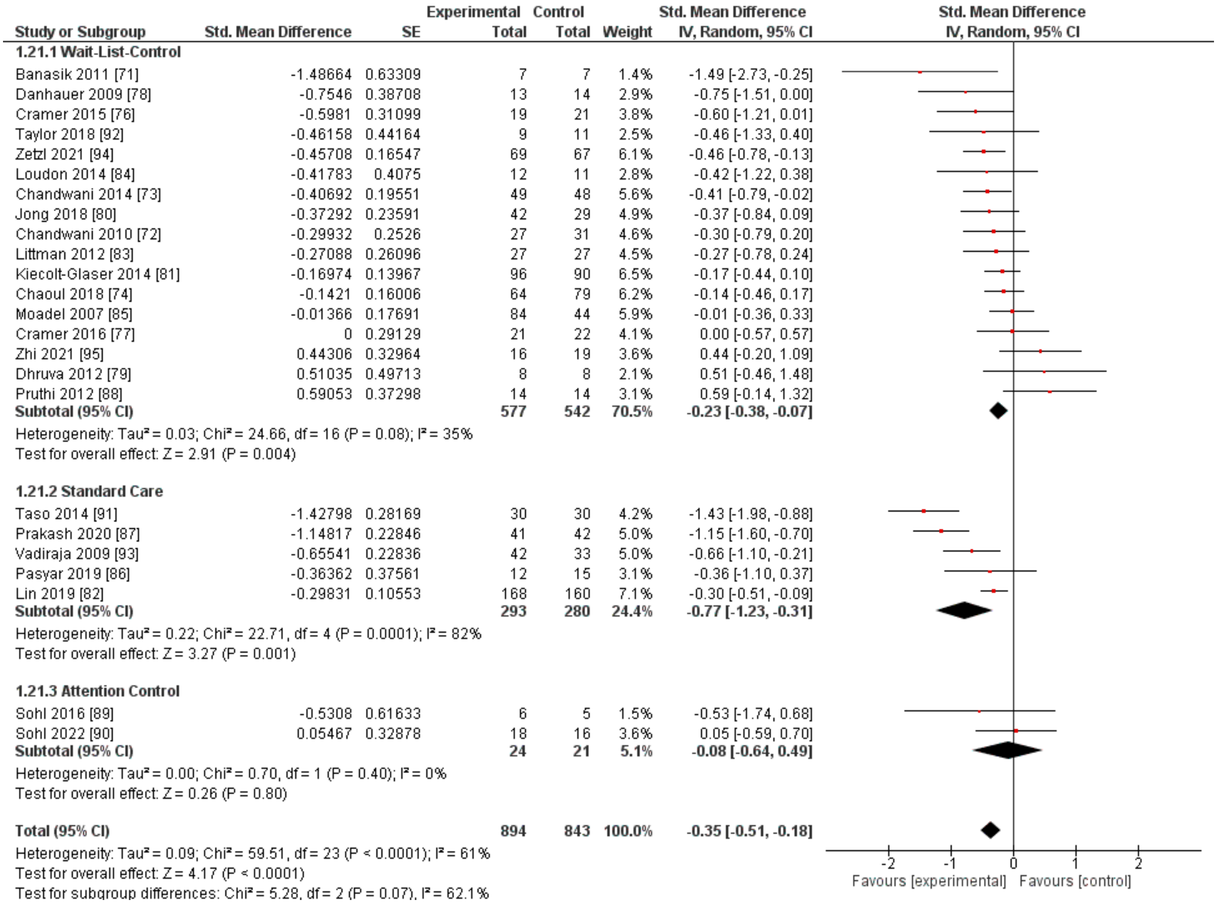

Figure S6. 1.4. Comparison of effects of yoga interventions with different control groups (waitlist-control vs. standard care vs. vs. attention control).

Section S6.2. Psychosocial Interventions

Study or Subgroup	Std. Mean Difference	SE	Experimental Total	Control Total	Weight	Std. Mean Difference IV, Random, 95% CI
**1.37.1 Studies without high risk of bias**						
Peng 2019 [111]	-1.58714	0.17825	80	75	1.8%	-1.59 [-1.94, -1.24]
Reif 2013 [114]	-1.46785	0.14195	120	114	1.9%	-1.47 [-1.75, -1.19]
Sadeghi 2016 [115]	-1.35144	0.18399	69	66	1.8%	-1.35 [-1.71, -0.99]
Cohen & Fried 2007 [100]	-0.98294	0.23523	38	37	1.7%	-0.98 [-1.44, -0.52]
Sheikhzadeh 2021 [118]	-0.88708	0.32487	19	20	1.4%	-0.89 [-1.52, -0.25]
Yates 2005 [123]	-0.83769	0.196	50	54	1.8%	-0.84 [-1.22, -0.45]
Tu 2021 [119]	-0.60879	0.19534	50	50	1.8%	-0.61 [-0.99, -0.23]
Kim 2018 [108]	-0.59796	0.26445	29	26	1.6%	-0.60 [-1.12, -0.08]
Dolbeault 2009 [102]	-0.52081	0.14953	81	87	1.9%	-0.52 [-0.81, -0.23]
Goedendorp 2010 [106]	-0.51072	0.1592	76	72	1.9%	-0.51 [-0.82, -0.20]
Xian 2021 [122]	-0.44754	0.17684	60	59	1.8%	-0.45 [-0.79, -0.10]
Fukui 2000 [103]	-0.37984	0.2728	25	25	1.6%	-0.38 [-0.91, 0.15]
Montgomery 2014 [109]	-0.36308	0.14256	91	90	1.9%	-0.36 [-0.64, -0.08]
Gaston-Johansson 2000 [104]	-0.34465	0.18317	52	58	1.8%	-0.34 [-0.70, 0.01]
Gregoire 2020 [107]	-0.33071	0.19678	48	47	1.8%	-0.33 [-0.72, 0.05]
Armes 2007 [97]	-0.32691	0.32344	20	16	1.4%	-0.33 [-0.96, 0.31]
Ream 2006 [113]	-0.24733	0.20615	43	43	1.8%	-0.25 [-0.65, 0.16]
Zhang 2020 [125]	-0.24272	0.15852	73	72	1.9%	-0.24 [-0.55, 0.07]
Van der Meulen 2014 [120]	-0.24166	0.14263	88	91	1.9%	-0.24 [-0.52, 0.04]
Courtier 2022 [101]	-0.21976	0.36077	20	11	1.3%	-0.22 [-0.93, 0.49]
Purcell 2011 [112]	-0.214	0.28623	21	24	1.5%	-0.21 [-0.78, 0.35]
Vargas 2014 [121]	-0.12503	0.13656	98	96	2.0%	-0.13 [-0.39, 0.14]
Andersen 2004 [96]	-0.05095	0.13618	106	90	2.0%	-0.05 [-0.32, 0.22]
Arving 2007 EG2 [98]	0	0.21283	40	40	1.7%	0.00 [-0.42, 0.42]
Bourmaud 2017 [99]	0.10555	0.1569	73	74	1.9%	0.11 [-0.20, 0.41]
Arving 2007 EG1 [98]	0.1877	0.20327	49	40	1.8%	0.19 [-0.21, 0.59]
Yuen 2006 [124]	0.20084	0.56799	6	6	0.8%	0.20 [-0.91, 1.31]
O'Brien 2014 [110]	0.38143	0.38221	14	12	1.2%	0.38 [-0.37, 1.13]
**Subtotal (95% CI)**			**1539**	**1495**	**47.8%**	**-0.45 [-0.64, -0.26]**
Heterogeneity: Tau² = 0.21; Chi² = 182.41, df = 27 (P < 0.00001); I² = 85%						
Test for overall effect: Z = 4.70 (P < 0.00001)						
**1.37.2 All studies**						
Peng 2019 [111]	-1.58714	0.17825	80	75	1.8%	-1.59 [-1.94, -1.24]
Reif 2013 [114]	-1.46785	0.14195	120	114	1.9%	-1.47 [-1.75, -1.19]
Sadeghi 2016 [115]	-1.35144	0.18399	69	66	1.8%	-1.35 [-1.71, -0.99]
Cohen & Fried 2007 [100]	-0.98294	0.23523	38	37	1.7%	-0.98 [-1.44, -0.52]
Sheikhzadeh 2021 [118]	-0.88708	0.32487	19	20	1.4%	-0.89 [-1.52, -0.25]
Yates 2005 [123]	-0.83769	0.196	50	54	1.8%	-0.84 [-1.22, -0.45]
Tu 2021 [119]	-0.60879	0.19534	50	50	1.8%	-0.61 [-0.99, -0.23]
Kim 2018 [108]	-0.59796	0.26445	29	26	1.6%	-0.60 [-1.12, -0.08]
Dolbeault 2009 [102]	-0.52081	0.14953	81	87	1.9%	-0.52 [-0.81, -0.23]
Goedendorp 2010 [106]	-0.51072	0.1592	76	72	1.9%	-0.51 [-0.82, -0.20]
Xian 2021 [122]	-0.44754	0.17684	60	59	1.8%	-0.45 [-0.79, -0.10]
Godino 2006 [105]	-0.43423	0.459	13	7	1.0%	-0.43 [-1.33, 0.47]
Fukui 2000 [103]	-0.37984	0.2728	25	25	1.6%	-0.38 [-0.91, 0.15]
Montgomery 2014 [109]	-0.36308	0.14256	91	90	1.9%	-0.36 [-0.64, -0.08]
Gaston-Johansson 2000 [104]	-0.34465	0.18317	52	58	1.8%	-0.34 [-0.70, 0.01]
Gregoire 2020 [107]	-0.33071	0.19678	48	47	1.8%	-0.33 [-0.72, 0.05]
Armes 2007 [97]	-0.32691	0.32344	20	16	1.4%	-0.33 [-0.96, 0.31]
Salvetti 2021 [116]	-0.25268	0.23449	31	36	1.7%	-0.25 [-0.71, 0.21]
Ream 2006 [113]	-0.24733	0.20615	43	43	1.8%	-0.25 [-0.65, 0.16]
Zhang 2020 [125]	-0.24272	0.15852	73	72	1.9%	-0.24 [-0.55, 0.07]
Van der Meulen 2014 [120]	-0.24166	0.14263	88	91	1.9%	-0.24 [-0.52, 0.04]
Courtier 2022 [101]	-0.21976	0.36077	20	11	1.3%	-0.22 [-0.93, 0.49]
Purcell 2011 [112]	-0.214	0.28623	21	24	1.5%	-0.21 [-0.78, 0.35]
Vargas 2014 [121]	-0.12503	0.13656	98	96	2.0%	-0.13 [-0.39, 0.14]
Andersen 2004 [96]	-0.05095	0.13618	106	90	2.0%	-0.05 [-0.32, 0.22]
Arving 2007 EG2 [98]	0	0.21283	40	40	1.7%	0.00 [-0.42, 0.42]
Schjolberg 2014 [117]	0.08881	0.2136	29	63	1.7%	0.09 [-0.33, 0.51]
Bourmaud 2017 [99]	0.10555	0.1569	73	74	1.9%	0.11 [-0.20, 0.41]
Arving 2007 EG1 [98]	0.1877	0.20327	49	40	1.8%	0.19 [-0.21, 0.59]
Yuen 2006 [124]	0.20084	0.56799	6	6	0.8%	0.20 [-0.91, 1.31]
O'Brien 2014 [110]	0.38143	0.38221	14	12	1.2%	0.38 [-0.37, 1.13]
**Subtotal (95% CI)**			**1612**	**1601**	**52.2%**	**-0.43 [-0.60, -0.25]**
Heterogeneity: Tau² = 0.20; Chi² = 189.71, df = 30 (P < 0.00001); I² = 84%						
Test for overall effect: Z = 4.71 (P < 0.00001)						
**Total (95% CI)**			**3151**	**3096**	**100.0%**	**-0.44 [-0.57, -0.31]**
Heterogeneity: Tau² = 0.20; Chi² = 372.27, df = 58 (P < 0.00001); I² = 84%						
Test for overall effect: Z = 6.73 (P < 0.00001)						
Test for subgroup differences: Chi² = 0.03, df = 1 (P = 0.85), I² = 0%						

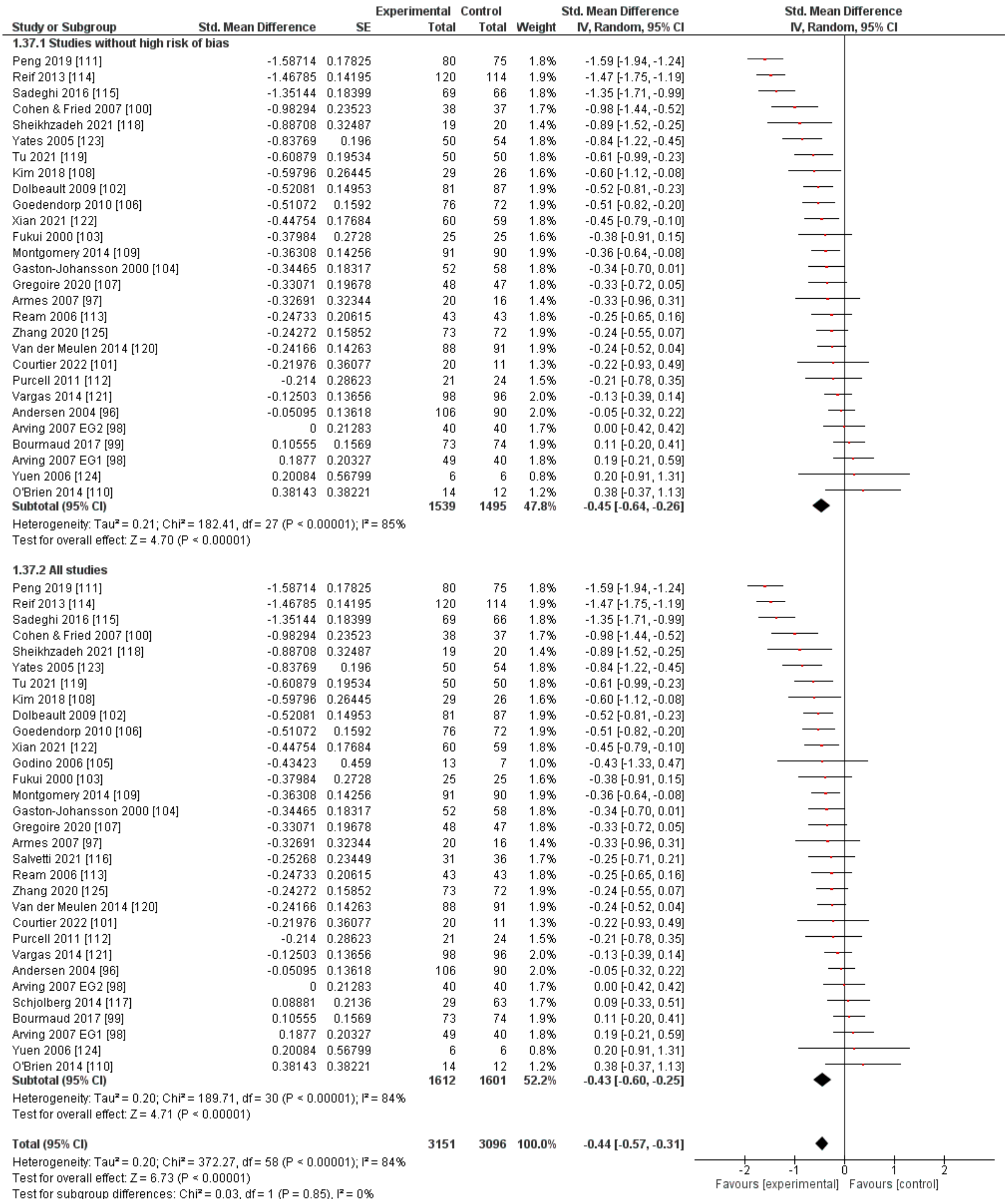

Figure S6.2.1. Sensitivity analysis for the comparison of the effect of psychosocial interventions without high risk of bias vs. all studies.
Note: High risk of bias was defined as at least one assessed high risk besides blinding of participants and personnel as well as blinding of outcome assessment.

Study or Subgroup	Std. Mean Difference	SE	Experimental Total	Control Total	Weight	Std. Mean Difference IV, Random, 95% CI
**1.35.1 Studies with n ≥25 per intervention arm**						
Peng 2019 [111]	-1.58714	0.17825	80	75	2.0%	-1.59 [-1.94, -1.24]
Reif 2013 [114]	-1.46785	0.14195	120	114	2.1%	-1.47 [-1.75, -1.19]
Sadeghi 2016 [115]	-1.35144	0.18399	69	66	1.9%	-1.35 [-1.71, -0.99]
Cohen & Fried 2007 [100]	-0.98294	0.23523	38	37	1.8%	-0.98 [-1.44, -0.52]
Yates 2005 [123]	-0.83769	0.196	50	54	1.9%	-0.84 [-1.22, -0.45]
Tu 2021 [119]	-0.60879	0.19534	50	50	1.9%	-0.61 [-0.99, -0.23]
Kim 2018 [108]	-0.59796	0.26445	29	26	1.7%	-0.60 [-1.12, -0.08]
Dolbeault 2009 [102]	-0.52081	0.14953	81	87	2.0%	-0.52 [-0.81, -0.23]
Goedendorp 2010 [106]	-0.51072	0.1592	76	72	2.0%	-0.51 [-0.82, -0.20]
Xian 2021 [122]	-0.44754	0.17684	60	59	2.0%	-0.45 [-0.79, -0.10]
Montgomery 2014 [109]	-0.36308	0.14256	91	90	2.1%	-0.36 [-0.64, -0.08]
Gaston-Johansson 2000 [104]	-0.34465	0.18317	52	58	1.9%	-0.34 [-0.70, 0.01]
Gregoire 2020 [107]	-0.33071	0.19678	48	47	1.9%	-0.33 [-0.72, 0.05]
Salvetti 2021 [116]	-0.25268	0.23449	31	36	1.8%	-0.25 [-0.71, 0.21]
Ream 2006 [113]	-0.24733	0.20615	43	43	1.9%	-0.25 [-0.65, 0.16]
Zhang 2020 [125]	-0.24272	0.15852	73	72	2.0%	-0.24 [-0.55, 0.07]
Van der Meulen 2014 [120]	-0.24166	0.14263	88	91	2.1%	-0.24 [-0.52, 0.04]
Vargas 2014 [121]	-0.12503	0.13656	98	96	2.1%	-0.13 [-0.39, 0.14]
Andersen 2004 [96]	-0.05095	0.13618	106	90	2.1%	-0.05 [-0.32, 0.22]
Arving 2007 EG2 [98]	0	0.21283	40	40	1.8%	0.00 [-0.42, 0.42]
Schjolberg 2014 [117]	0.08881	0.2136	29	63	1.8%	0.09 [-0.33, 0.51]
Bourmaud 2017 [99]	0.10555	0.1569	73	74	2.0%	0.11 [-0.20, 0.41]
Arving 2007 EG1 [98]	0.1877	0.20327	49	40	1.9%	0.19 [-0.21, 0.59]
**Subtotal (95% CI)**			**1474**	**1480**	**44.5%**	**-0.47 [-0.67, -0.26]**
Heterogeneity: Tau² = 0.22; Chi² = 180.47, df = 22 (P < 0.00001); I² = 88%						
Test for overall effect: Z = 4.45 (P < 0.00001)						
**1.35.2 All studies**						
Peng 2019 [111]	-1.58714	0.17825	80	75	2.0%	-1.59 [-1.94, -1.24]
Reif 2013 [114]	-1.46785	0.14195	120	114	2.1%	-1.47 [-1.75, -1.19]
Sadeghi 2016 [115]	-1.35144	0.18399	69	66	1.9%	-1.35 [-1.71, -0.99]
Cohen & Fried 2007 [100]	-0.98294	0.23523	38	37	1.8%	-0.98 [-1.44, -0.52]
Sheikhzadeh 2021 [118]	-0.88708	0.32487	19	20	1.5%	-0.89 [-1.52, -0.25]
Yates 2005 [123]	-0.83769	0.196	50	54	1.9%	-0.84 [-1.22, -0.45]
Tu 2021 [119]	-0.60879	0.19534	50	50	1.9%	-0.61 [-0.99, -0.23]
Kim 2018 [108]	-0.59796	0.26445	29	26	1.7%	-0.60 [-1.12, -0.08]
Dolbeault 2009 [102]	-0.52081	0.14953	81	87	2.0%	-0.52 [-0.81, -0.23]
Goedendorp 2010 [106]	-0.51072	0.1592	76	72	2.0%	-0.51 [-0.82, -0.20]
Xian 2021 [122]	-0.44754	0.17684	60	59	2.0%	-0.45 [-0.79, -0.10]
Godino 2006 [105]	-0.43423	0.459	13	7	1.1%	-0.43 [-1.33, 0.47]
Fukui 2000 [103]	-0.37984	0.2728	25	25	1.7%	-0.38 [-0.91, 0.15]
Montgomery 2014 [109]	-0.36308	0.14256	91	90	2.1%	-0.36 [-0.64, -0.08]
Gaston-Johansson 2000 [104]	-0.34465	0.18317	52	58	1.9%	-0.34 [-0.70, 0.01]
Gregoire 2020 [107]	-0.33071	0.19678	48	47	1.9%	-0.33 [-0.72, 0.05]
Armes 2007 [97]	-0.32691	0.32344	20	16	1.5%	-0.33 [-0.96, 0.31]
Salvetti 2021 [116]	-0.25268	0.23449	31	36	1.8%	-0.25 [-0.71, 0.21]
Ream 2006 [113]	-0.24733	0.20615	43	43	1.9%	-0.25 [-0.65, 0.16]
Zhang 2020 [125]	-0.24272	0.15852	73	72	2.0%	-0.24 [-0.55, 0.07]
Van der Meulen 2014 [120]	-0.24166	0.14263	88	91	2.1%	-0.24 [-0.52, 0.04]
Courtier 2022 [101]	-0.21976	0.36077	20	11	1.4%	-0.22 [-0.93, 0.49]
Purcell 2011 [112]	-0.214	0.28623	21	24	1.6%	-0.21 [-0.78, 0.35]
Vargas 2014 [121]	-0.12503	0.13656	98	96	2.1%	-0.13 [-0.39, 0.14]
Andersen 2004 [96]	-0.05095	0.13618	106	90	2.1%	-0.05 [-0.32, 0.22]
Arving 2007 EG2 [98]	0	0.21283	40	40	1.8%	0.00 [-0.42, 0.42]
Schjolberg 2014 [117]	0.08881	0.2136	29	63	1.8%	0.09 [-0.33, 0.51]
Bourmaud 2017 [99]	0.10555	0.1569	73	74	2.0%	0.11 [-0.20, 0.41]
Arving 2007 EG1 [98]	0.1877	0.20327	49	40	1.9%	0.19 [-0.21, 0.59]
Yuen 2006 [124]	0.20084	0.56799	6	6	0.9%	0.20 [-0.91, 1.31]
O'Brien 2014 [110]	0.38143	0.38221	14	12	1.3%	0.38 [-0.37, 1.13]
**Subtotal (95% CI)**			**1612**	**1601**	**55.5%**	**-0.43 [-0.60, -0.25]**
Heterogeneity: Tau² = 0.20; Chi² = 189.71, df = 30 (P < 0.00001); I² = 84%						
Test for overall effect: Z = 4.71 (P < 0.00001)						
**Total (95% CI)**			**3086**	**3081**	**100.0%**	**-0.44 [-0.58, -0.31]**
Heterogeneity: Tau² = 0.20; Chi² = 370.24, df = 53 (P < 0.00001); I² = 86%						
Test for overall effect: Z = 6.55 (P < 0.00001)						
Test for subgroup differences: Chi² = 0.08, df = 1 (P = 0.77), I² = 0%						

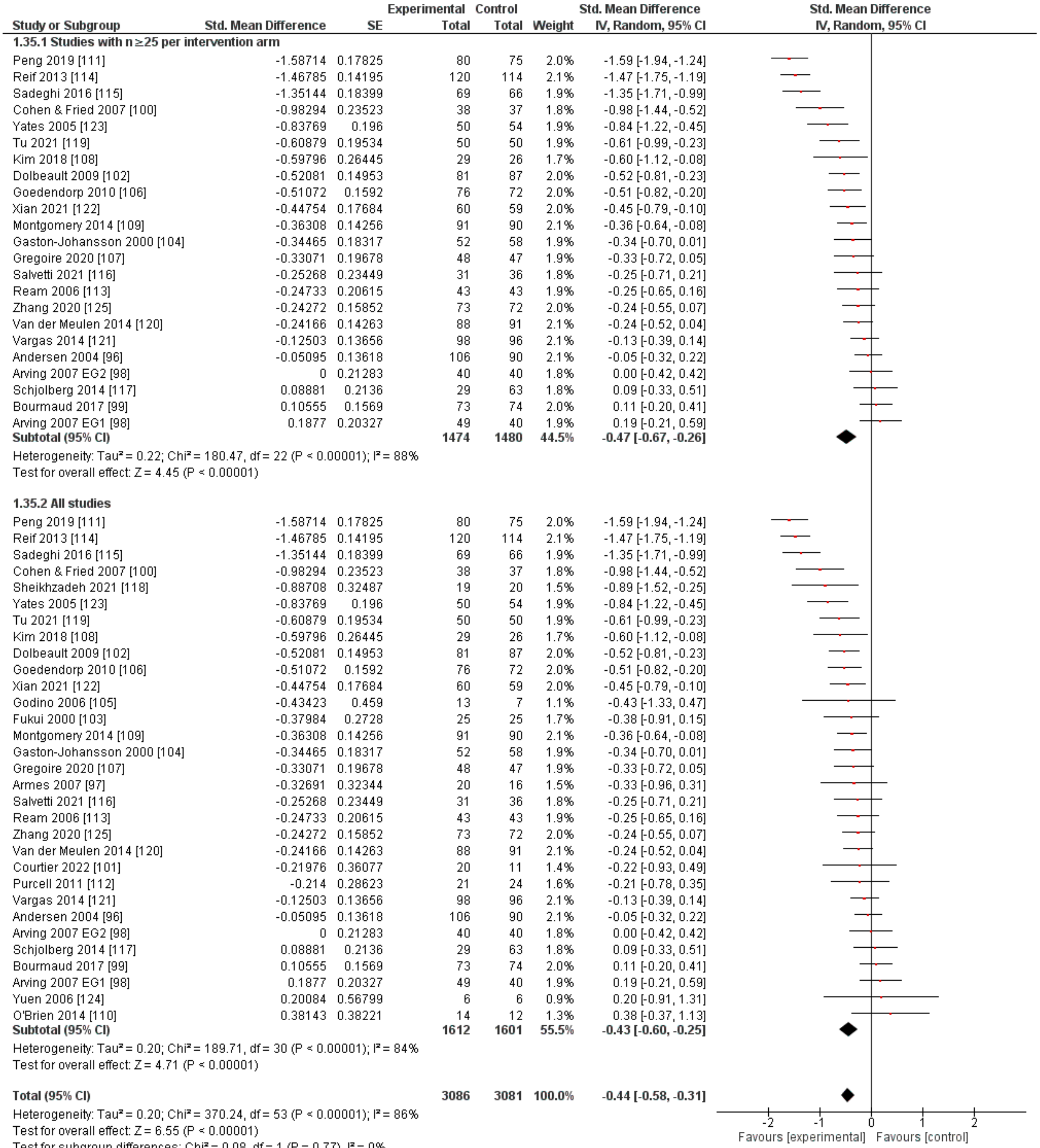

Figure S6.2.2. Sensitivity analysis for the comparison of the effect of psychosocial interventions with more than 25 patients per intervention arm vs. all studies.

Study or Subgroup	Mean Difference	SE	Experimental Total	Control Total	Weight	Mean Difference IV, Random, 95% CI
**1.29.1 Standard Care**						
Peng 2019 [111]	-1.58714	0.17825	80	75	3.5%	-1.59 [-1.94, -1.24]
Cohen & Fried 2007 [100]	-0.98294	0.23523	38	37	3.2%	-0.98 [-1.44, -0.52]
Tu 2021 [119]	-0.60879	0.19534	50	50	3.4%	-0.61 [-0.99, -0.23]
Kim 2018 [108]	-0.59796	0.26445	29	26	3.0%	-0.60 [-1.12, -0.08]
Goedendorp 2010 [106]	-0.51072	0.1592	76	72	3.6%	-0.51 [-0.82, -0.20]
Xian 2021 [122]	-0.44754	0.17684	60	59	3.5%	-0.45 [-0.79, -0.10]
Godino 2006 [105]	-0.43423	0.459	13	7	2.0%	-0.43 [-1.33, 0.47]
Gaston-Johansson 2000 [104]	-0.34465	0.18317	52	58	3.5%	-0.34 [-0.70, 0.01]
Armes 2007 [97]	-0.32691	0.32344	20	16	2.7%	-0.33 [-0.96, 0.31]
Salvetti 2021 [116]	-0.25268	0.23449	31	36	3.2%	-0.25 [-0.71, 0.21]
Ream 2006 [113]	-0.24733	0.20615	43	43	3.4%	-0.25 [-0.65, 0.16]
Zhang 2020 [125]	-0.24272	0.15852	73	72	3.6%	-0.24 [-0.55, 0.07]
Van der Meulen 2014 [120]	-0.24166	0.14263	88	91	3.7%	-0.24 [-0.52, 0.04]
Courtier 2022 [101]	-0.21976	0.36077	20	11	2.5%	-0.22 [-0.93, 0.49]
Purcell 2011 [112]	-0.214	0.28623	21	24	2.9%	-0.21 [-0.78, 0.35]
Andersen 2004 [96]	-0.05095	0.13618	106	90	3.7%	-0.05 [-0.32, 0.22]
Arving 2007 EG2 [98]	0	0.21283	40	40	3.3%	0.00 [-0.42, 0.42]
Schjolberg 2014 [117]	0.08881	0.2136	29	63	3.3%	0.09 [-0.33, 0.51]
Arving 2007 EG1 [98]	0.1877	0.20327	49	40	3.4%	0.19 [-0.21, 0.59]
Yuen 2006 [124]	0.20084	0.56799	6	6	1.6%	0.20 [-0.91, 1.31]
O'Brien 2014 [110]	0.38143	0.38221	14	12	2.4%	0.38 [-0.37, 1.13]
**Subtotal (95% CI)**			**938**	**928**	**65.5%**	**-0.33 [-0.53, -0.14]**
Heterogeneity: Tau² = 0.14; Chi² = 84.57, df = 20 (P < 0.00001); I² = 76%						
Test for overall effect: Z = 3.42 (P = 0.0006)						
**1.29.2 Wait-List-Control**						
Reif 2013 [114]	-1.46785	0.14195	120	114	3.7%	-1.47 [-1.75, -1.19]
Sadeghi 2016 [115]	-1.35144	0.18399	69	66	3.5%	-1.35 [-1.71, -0.99]
Sheikhzadeh 2021 [118]	-0.88708	0.32487	19	20	2.7%	-0.89 [-1.52, -0.25]
Dolbeault 2009 [102]	-0.52081	0.14953	81	87	3.7%	-0.52 [-0.81, -0.23]
Fukui 2000 [103]	-0.37984	0.2728	25	25	3.0%	-0.38 [-0.91, 0.15]
Gregoire 2020 [107]	-0.33071	0.19678	48	47	3.4%	-0.33 [-0.72, 0.05]
Bourmaud 2017 [99]	0.10555	0.1569	73	74	3.6%	0.11 [-0.20, 0.41]
**Subtotal (95% CI)**			**435**	**433**	**23.6%**	**-0.69 [-1.18, -0.20]**
Heterogeneity: Tau² = 0.39; Chi² = 74.73, df = 6 (P < 0.00001); I² = 92%						
Test for overall effect: Z = 2.78 (P = 0.006)						
**1.29.3 Attention Control**						
Yates 2005 [123]	-0.83769	0.196	50	54	3.4%	-0.84 [-1.22, -0.45]
Montgomery 2014 [109]	-0.36308	0.14256	91	90	3.7%	-0.36 [-0.64, -0.08]
Vargas 2014 [121]	-0.12503	0.13656	98	96	3.7%	-0.13 [-0.39, 0.14]
**Subtotal (95% CI)**			**239**	**240**	**10.9%**	**-0.42 [-0.79, -0.05]**
Heterogeneity: Tau² = 0.08; Chi² = 8.90, df = 2 (P = 0.01); I² = 78%						
Test for overall effect: Z = 2.20 (P = 0.03)						
**Total (95% CI)**			**1612**	**1601**	**100.0%**	**-0.43 [-0.60, -0.25]**
Heterogeneity: Tau² = 0.20; Chi² = 189.71, df = 30 (P < 0.00001); I² = 84%						
Test for overall effect: Z = 4.71 (P < 0.00001)						
Test for subgroup differences: Chi² = 1.81, df = 2 (P = 0.41), I² = 0%						

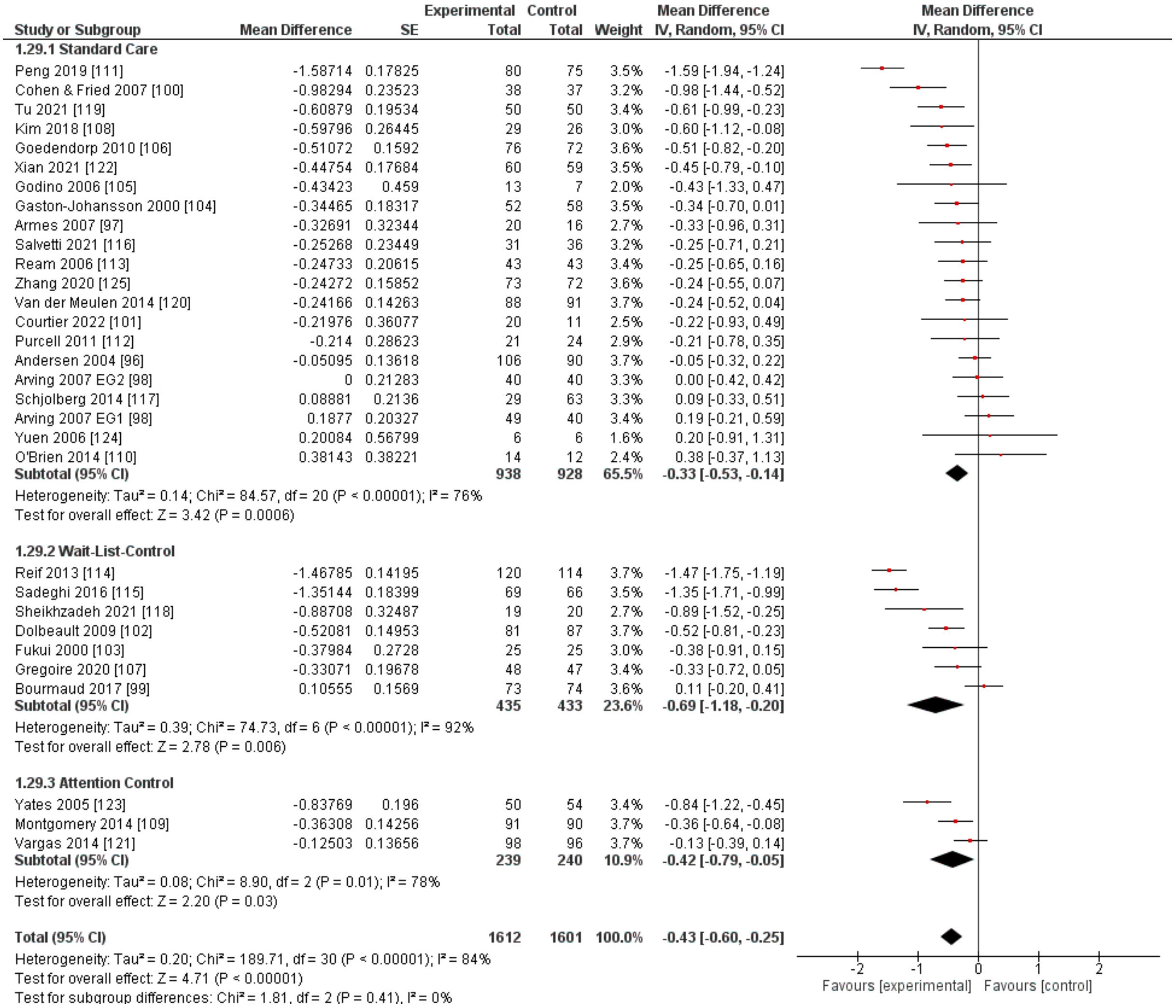

Figure S6.2.3. Comparison of effects of psychosocial interventions with different control groups (standard care vs. waitlist-control vs. attention control).

General note: For psychosocial interventions, we did not perform a sensitivity analysis with respect to a specific study population because this only concerned the study from Kim (2018 [108]; study population at high risk for depression), whose SMD was close to the mean effect (SMD = -0.60; 95% CI -1.12, -0.08)).

Section S6.3. Mindfulness-based Interventions

Study or Subgroup	Std. Mean Difference	SE	Experimental Total	Control Total	Weight	Std. Mean Difference IV, Random, 95% CI
**1.11.1 Studies with n ≥25 per intervention arm**						
Liu 2019 [133]	-1.7055	0.22506	49	53	4.0%	-1.71 [-2.15, -1.26]
Gok Metin 2019 [128]	-0.89918	0.25885	32	29	3.7%	-0.90 [-1.41, -0.39]
Park 2020 [135]	-0.79615	0.23169	38	36	4.0%	-0.80 [-1.25, -0.34]
Ng 2021 [134]	-0.75125	0.22452	38	40	4.0%	-0.75 [-1.19, -0.31]
Bower 2021 [127]	-0.54298	0.15907	77	72	4.7%	-0.54 [-0.85, -0.23]
Bower 2015 [126]	-0.52003	0.24178	35	30	3.9%	-0.52 [-0.99, -0.05]
Lengacher 2012 [131]	-0.35999	0.21228	40	42	4.1%	-0.36 [-0.78, 0.06]
Lengacher 2016 [132]	-0.3429	0.11075	152	147	5.1%	-0.34 [-0.56, -0.13]
Hoffman 2012 [129]	-0.33473	0.13098	103	111	4.9%	-0.33 [-0.59, -0.08]
Witek Janusek 2019 [139]	-0.11005	0.16259	70	67	4.6%	-0.11 [-0.43, 0.21]
**Subtotal (95% CI)**			**634**	**627**	**42.9%**	**-0.61 [-0.86, -0.36]**
Heterogeneity: Tau² = 0.12; Chi² = 43.81, df = 9 (P < 0.00001); I² = 79%						
Test for overall effect: Z = 4.80 (P < 0.00001)						
**1.11.2 All studies**						
Rahmani 2014 [136]	-1.81888	0.49409	12	12	2.0%	-1.82 [-2.79, -0.85]
Johns 2015 [130]	-1.8047	0.40036	18	17	2.5%	-1.80 [-2.59, -1.02]
Liu 2019 [133]	-1.7055	0.22506	49	53	4.0%	-1.71 [-2.15, -1.26]
Van der Lee 2012 [138]	-1.21064	0.25017	59	24	3.8%	-1.21 [-1.70, -0.72]
Gok Metin 2019 [128]	-0.89918	0.25885	32	29	3.7%	-0.90 [-1.41, -0.39]
Park 2020 [135]	-0.79615	0.23169	38	36	4.0%	-0.80 [-1.25, -0.34]
Ng 2021 [134]	-0.75125	0.22452	38	40	4.0%	-0.75 [-1.19, -0.31]
Sheikhzadeh 2021 [118]	-0.65274	0.3164	19	20	3.2%	-0.65 [-1.27, -0.03]
Bower 2021 [127]	-0.54298	0.15907	77	72	4.7%	-0.54 [-0.85, -0.23]
Bower 2015 [126]	-0.52003	0.24178	35	30	3.9%	-0.52 [-0.99, -0.05]
Lengacher 2012 [131]	-0.35999	0.21228	40	42	4.1%	-0.36 [-0.78, 0.06]
Lengacher 2016 [132]	-0.3429	0.11075	152	147	5.1%	-0.34 [-0.56, -0.13]
Hoffman 2012 [129]	-0.33473	0.13098	103	111	4.9%	-0.33 [-0.59, -0.08]
Witek Janusek 2019 [139]	-0.11005	0.16259	70	67	4.6%	-0.11 [-0.43, 0.21]
Van der Gucht 2020 [137]	-0.10156	0.3847	12	13	2.6%	-0.10 [-0.86, 0.65]
**Subtotal (95% CI)**			**754**	**713**	**57.1%**	**-0.73 [-0.98, -0.49]**
Heterogeneity: Tau² = 0.17; Chi² = 69.17, df = 14 (P < 0.00001); I² = 80%						
Test for overall effect: Z = 5.92 (P < 0.00001)						
**Total (95% CI)**			**1388**	**1340**	**100.0%**	**-0.68 [-0.85, -0.51]**
Heterogeneity: Tau² = 0.14; Chi² = 113.68, df = 24 (P < 0.00001); I² = 79%						
Test for overall effect: Z = 7.80 (P < 0.00001)						
Test for subgroup differences: Chi² = 0.48, df = 1 (P = 0.49), I² = 0%						

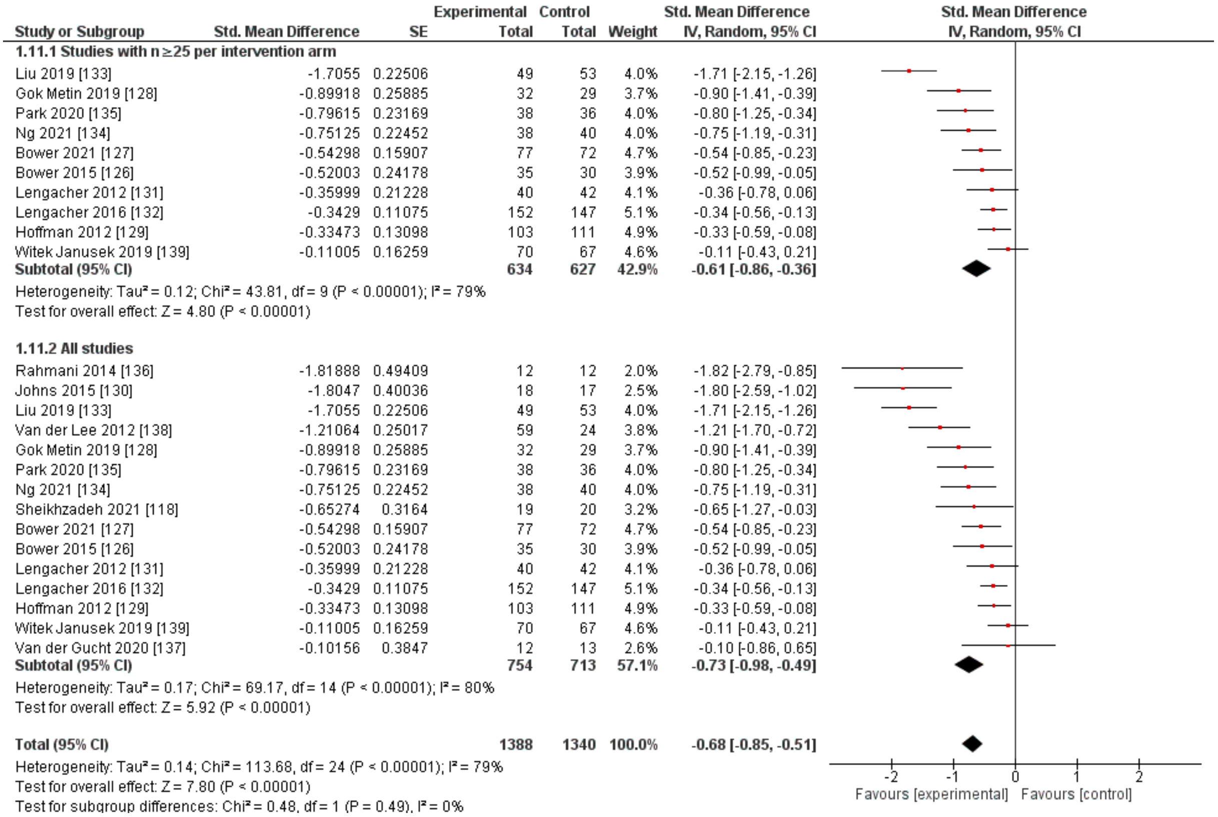

Figure S6.3.1. Sensitivity analysis for the comparison of the effect of mindfulness-based interventions with more than 25 patients per intervention arm vs. all studies.

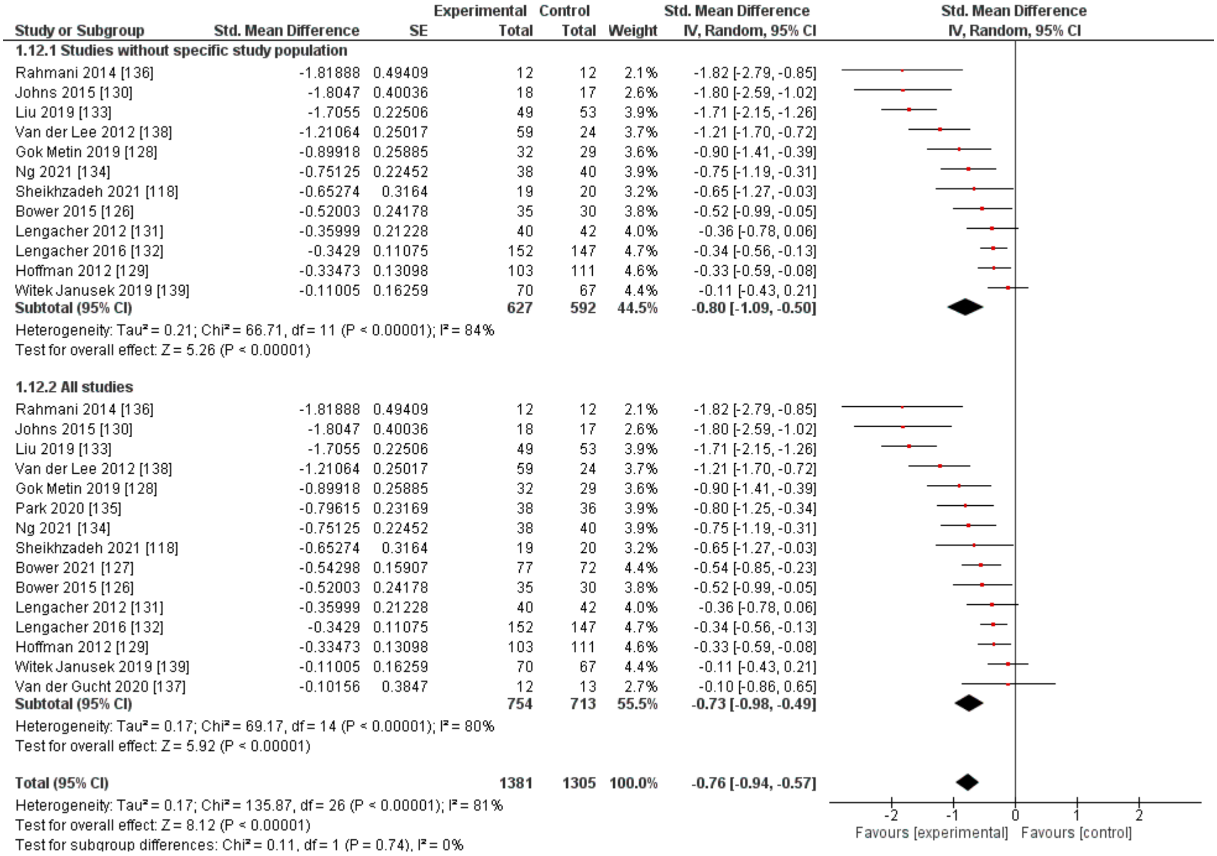

Figure S6.3.2. Sensitivity analysis for the comparison of mindfulness-based interventions including study populations without specific physical or mental impairment vs. all studies.
Note: Bower (2021) [127] specifically included study participants with depression; Park (2020) [135] with depression and anxiety; Van der Gucht (2020) [137] with cognitive impairments.

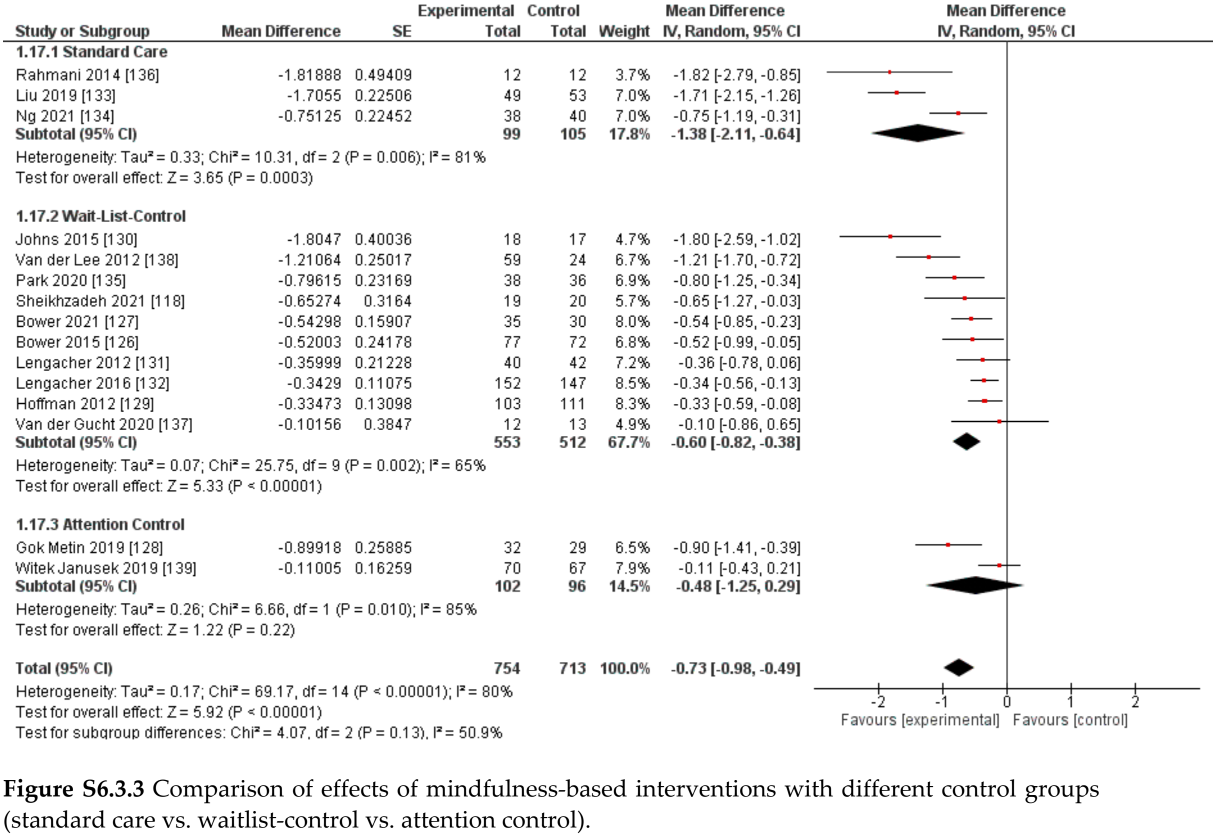

Figure S6.3.3 Comparison of effects of mindfulness-based interventions with different control groups (standard care vs. waitlist-control vs. attention control).

General note: We did not calculate a sensitivity analysis with regard to risk of bias for mindfulness-based interventions, because only the study by Van der Gucht (2020) [137] had a high bias (besides blinding of participants and personnel as well as blinding of outcome assessment), which showed an effect below-average on CRF (SMD = -0.10; 95% CI -0.86, 0.65).

References

82. Lin, P.J.; Kleckner, I.R.; Loh, K.P.; Inglis, J.E.; Peppone, L.J.; Janelsins, M.C.; Kamen, C.S.; Heckler, C.E.; Culakova, E.; Pigeon, W.R.; et al. Influence of Yoga on Cancer-Related Fatigue and on Mediational Relationships Between Changes in Sleep and Cancer-Related Fatigue: A Nationwide, Multicenter Randomized Controlled Trial of Yoga in Cancer Survivors. *Integr. Cancer Ther.* **2019**, *18*, 1534735419855134. https://doi.org/10.1177/1534735419855134.
95. Zhi, W.I.; Baser, R.E.; Zhi, L.M.; Talukder, D.; Li, Q.S.; Paul, T.; Patterson, C.; Piulson, L.; Seluzicki, C.; Galantino, M.L.; et al. Yoga for cancer survivors with chemotherapy-induced peripheral neuropathy: Health-related quality of life outcomes. *Cancer Med.* **2021**, *10*, 5456–5465. https://doi.org/10.1002/cam4.4098.
108. Kim, Y.H.; Choi, K.S.; Han, K.; Kim, H.W. A psychological intervention programme for patients with breast cancer under chemotherapy and at a high risk of depression: A randomised clinical trial. *J. Clin. Nurs.* **2018**, *27*, 572–581. https://doi.org/10.1111/jocn.13910.
127. Bower, J.E.; Partridge, A.H.; Wolff, A.C.; Thorner, E.D.; Irwin, M.R.; Joffe, H.; Petersen, L.; Crespi, C.M.; Ganz, P.A. Targeting Depressive Symptoms in Younger Breast Cancer Survivors: The Pathways to Wellness Randomized Controlled Trial of Mindfulness Meditation and Survivorship Education. *J. Clin. Oncol.* **2021**, *39*, 3473–3484. https://doi.org/10.1200/jco.21.00279.
135. Park, S.; Sato, Y.; Takita, Y.; Tamura, N.; Ninomiya, A.; Kosugi, T.; Sado, M.; Nakagawa, A.; Takahashi, M.; Hayashida, T.; et al. Mindfulness-Based Cognitive Therapy for Psychological Distress, Fear of Cancer Recurrence, Fatigue, Spiritual Well-Being, and Quality of Life in Patients with Breast Cancer-A Randomized Controlled Trial. *J. Pain Symptom Manag.* **2020**, *60*, 381–389. https://doi.org/10.1016/j.jpainsymman.2020.02.017.
137. Van der Gucht, K.; Ahmadoun, S.; Melis, M.; de Cloe, E.; Sleurs, C.; Radwan, A.; Blommaert, J.; Takano, K.; Vandenbulcke, M.; Wildiers, H.; et al. Effects of a mindfulness-based intervention on cancer-related cognitive impairment: Results of a randomized controlled functional magnetic resonance imaging pilot study. *Cancer* **2020**, *126*, 4246–4255. https://doi.org/10.1002/cncr.33074.
