# Supplementary material for: Meta-Analysis of Randomized Controlled Trials on Yoga, Psychosocial, and Mindfulness-Based Interventions for Cancer-Related Fatigue: What Intervention Characteristics Are Related to Higher Efficacy?
Source: Cancers (Basel). 2022 Apr 15;14(8):2016. doi: 10.3390/cancers14082016 (PMC9032769; doi:10.3390/cancers14082016)
Supplement: Supplementary file 1 [file cancers-14-02016-s001.zip › Supplementary Tables S1_search strategies_proof.pdf]

Table 1. 1. Search strategy for psychosocial interventions in PsycInfo and CINAHL.

ID	Search
#1	TI (psychosocial OR psychotherapy OR psychological OR cognitive OR behavior* OR CBT OR psychoeducat* OR imagery OR "energy conservation" OR "stress management" OR “self-management” OR “emotion regulation”) OR AB (psychosocial OR psychotherapy OR psychological OR cognitive OR behavior* OR CBT OR psychoeducat* OR imagery OR "energy conservation" OR "stress management" OR “self-management” OR “emotion regulation”)
#2	TI (cancer* OR tumor OR tumour OR malignancy OR radiotherapy OR neoplas* OR carcinoma* OR leukemi* OR leukaemia* OR lymphoma*OR "bone marrow transplant*" OR "stem cell transplant*" OR chemotherapy) OR AB (cancer* OR tumor OR tumour OR malignancy OR radiotherapy OR neoplas* OR carcinoma* OR leukemi* OR leukaemia* OR lymphoma*OR "bone marrow transplant*" OR "stem cell transplant*" OR chemotherapy)
#3	TI fatigue OR AB fatigue
#4	TI (random* OR RCT OR intervention) OR AB (random* OR RCT OR intervention)
#5	#1 AND #2 AND #3 AND #4

Note: * = all words beginning with the same letters before the asterisk were included in the search.

Table 1. 2. Search strategy for psychosocial interventions in Pubmed.

ID	Search
#1	psychosocial[Title/Abstract] OR psychotherapy[Title/Abstract] OR psychological[Title/Abstract] OR cognitive[Title/Abstract] OR behavior*[Title/Abstract] OR behaviour*[Title/Abstract] OR CBT[Title/Abstract] OR psychoeducat*[Title/Abstract] OR imagery[Title/Abstract] OR "energy conservation"[Title/Abstract] OR "stress management"[Title/Abstract] OR “self-management”[Title/Abstract] OR “emotion regulation”[Title/Abstract]
#2	cancer*[Title/Abstract] OR tumor[Title/Abstract] OR tumour[Title/Abstract] OR malignancy[Title/Abstract] OR radiotherapy[Title/Abstract] OR neoplas*[Title/Abstract] OR carcinoma*[Title/Abstract] OR leukemi*[Title/Abstract] OR leukaemia*[Title/Abstract] OR lymphoma*[Title/Abstract] OR "bone marrow transplant*"[Title/Abstract] OR "stem cell transplant*"[Title/Abstract] OR chemotherapy[Title/Abstract]
#3	fatigue[Title/Abstract]
#4	random*[Title/Abstract] OR RCT[Title/Abstract] OR intervention[Title/Abstract]
#5	#1 AND #2 AND #3 AND #4

Note: * = all words beginning with the same letters before the asterisk were included in the search.

Table 1. 3. Search strategy for mindfulness-based interventions in PsycInfo and CINAHL.

ID	Search
#1	TI (mindful OR mindfulness OR MBSR OR MBCT OR body-mind OR mind-body) OR AB (mindful OR mindfulness OR MBSR OR MBCT OR body-mind OR mind-body)
#2	TI (cancer* OR tumor OR tumour OR malignancy OR radiotherapy OR neoplas* OR carcinoma* OR leukemi* OR leukaemia* OR lymphoma*OR "bone marrow transplant*" OR "stem cell transplant*" OR chemotherapy) OR AB (cancer* OR tumor OR tumour OR malignancy OR radiotherapy OR neoplas* OR carcinoma* OR leukemi* OR leukaemia* OR lymphoma*OR "bone marrow transplant*" OR "stem cell transplant*" OR chemotherapy)
#3	TI fatigue OR AB fatigue
#4	TI (random* OR RCT OR intervention) OR AB (random* OR RCT OR intervention)
#5	#1 AND #2 AND #3 AND #4

Note: * = all words beginning with the same letters before the asterisk were included in the search.

Table 1. 4. Search strategy for mindfulness-based interventions in Pubmed.

ID	Search
#1	mindful[Title/Abstract] OR mindfulness[Title/Abstract] OR MBSR[Title/Abstract] OR MBCT[Title/Abstract] OR body-mind[Title/Abstract] OR mind-body[Title/Abstract]
#2	cancer*[Title/Abstract] OR tumor[Title/Abstract] OR tumour[Title/Abstract] OR malignancy[Title/Abstract] OR radiotherapy[Title/Abstract] OR neoplas*[Title/Abstract] OR carcinoma*[Title/Abstract] OR leukemi*[Title/Abstract]

	OR leukaemia*[Title/Abstract] OR lymphoma*[Title/Abstract] OR "bone marrow transplant*"[Title/Abstract] OR "stem cell transplant*"[Title/Abstract] OR chemotherapy[Title/Abstract]
#3	fatigue[Title/Abstract]
#4	random*[Title/Abstract] OR RCT[Title/Abstract] OR intervention[Title/Abstract]
#5	#1 AND #2 AND #3 AND #4

Note: * = all words beginning with the same letters before the asterisk were included in the search.

Table 1. 5. Search strategy for yoga interventions in PsycInfo and CINAHL.

ID	Search
#1	TI (yoga OR yogic OR asana OR pranayama OR Dhyana OR dharana) OR AB (yoga OR yogic OR asana OR pranayama OR Dhyana OR dharana)
#2	TI (cancer* OR tumor OR tumour OR malignancy OR radiotherapy OR neoplas* OR carcinoma* OR leukemi* OR leukaemia* OR lymphoma*OR "bone marrow transplant*" OR "stem cell transplant*" OR chemotherapy) OR AB (cancer* OR tumor OR tumour OR malignancy OR radiotherapy OR neoplas* OR carcinoma* OR leukemi* OR leukaemia* OR lymphoma*OR "bone marrow transplant*" OR "stem cell transplant*" OR chemotherapy)
#3	TI fatigue OR AB fatigue
#4	TI (random* OR RCT OR intervention) OR AB (random* OR RCT OR intervention)
#5	#1 AND #2 AND #3 AND #4

Note: * = all words beginning with the same letters before the asterisk were included in the search.

Table 1. 6. Search strategy for yoga interventions in Pubmed.

ID	Search
#1	yoga[Title/Abstract] OR yogic[Title/Abstract] OR asana[Title/Abstract] OR pranayama[Title/Abstract] OR Dhyana[Title/Abstract] OR dharana[Title/Abstract]
#2	cancer*[Title/Abstract] OR tumor[Title/Abstract] OR tumour[Title/Abstract] OR malignancy[Title/Abstract] OR radiotherapy[Title/Abstract] OR neoplas*[Title/Abstract] OR carcinoma*[Title/Abstract] OR leukemi*[Title/Abstract] OR leukaemia*[Title/Abstract] OR lymphoma*[Title/Abstract] OR "bone marrow transplant*"[Title/Abstract] OR "stem cell transplant*"[Title/Abstract] OR chemotherapy[Title/Abstract]
#3	fatigue[Title/Abstract]
#4	random*[Title/Abstract] OR RCT[Title/Abstract] OR intervention[Title/Abstract]
#5	#1 AND #2 AND #3 AND #4

Note: * = all words beginning with the same letters before the asterisk were included in the search.
