# Supplementary material for: Meta-Analysis of Randomized Controlled Trials on Yoga, Psychosocial, and Mindfulness-Based Interventions for Cancer-Related Fatigue: What Intervention Characteristics Are Related to Higher Efficacy?
Source: Cancers (Basel). 2022 Apr 15;14(8):2016. doi: 10.3390/cancers14082016 (PMC9032769; doi:10.3390/cancers14082016)
Supplement: Supplementary file 1 [file cancers-14-02016-s001.zip › Supplementary Tables S2_Description of interventions_Proof.pdf]

Table S2. 1. Characteristics of included yoga interventions.

Study (Author + Year + Country)	Description of study population	Instrument(s) used to measure fatigue[a]	Description of intervention[b]	Description of control
Banasik [71] (2011) United States	*n* = 14<br>M_{Age} = 62.9<br>Breast Cancer<br>Stage: II-IV<br>After treatment (not reported which)	**FACT-B**	Content: Iyengar yoga, moderate physical effort, variety between sessions<br>Setting: group-based[c]<br>Duration: 8 weeks<br>Session number: 16 (*M* = 14)<br>Session length: 1.5h<br>Additional home practice: no	Wait-List Control
Chandwani [72] (2010) United States	*n* = 58<br>M_{Age} = 45.7<br>Breast Cancer<br>Stage: 0-III<br>During treatment (radiotherapy, some after surgery and/or chemotherapy)	**BFI**	Content: based on Patanjali´s yoga tradition, breathing techniques, meditation, moderate physical effort, no variety between sessions<br>Setting: individually (majority)<br>Duration: 6 weeks<br>Session number: 12 (*M* = 10.23[c])<br>Session length: 1h<br>Additional home practice: yes (daily; practice log)	Wait-List Control
Chandwani [73] (2014) United States	*n* = 97<br>M_{Age} = 51.8<br>Breast Cancer<br>Stage: 0-III<br>During treatment (adjuvant radiotherapy, some after surgery and/or chemotherapy)	**BFI**	Content: based on Patanjali´s yoga tradition, breathing techniques, meditation, moderate physical effort, no variety between sessions<br>Setting: individually (majority)<br>Duration: 6 weeks<br>Session number: up to 18 (*M* = 13.8)<br>Session length: 1h<br>Additional home practice: yes (no specific time recommended; practice log)	Wait-List Control
Chaoul [74] (2018) United States	*n* = 143<br>M_{Age} = 49.2<br>Breast Cancer<br>Stage: I-III	**BFI**	Content: Tibetan yoga, breathing techniques, meditation, imagery, mild physical effort, no variety between sessions<br>Setting: individually (majority)	Wait-List Control

	During treatment (neo-/adjuvant chemotherapy)		Duration: length of chemotherapy (majority completed within 12 weeks)<br>Session number: 4 (M = 3.28[c]) + 3 booster sessions during follow-up (M = 0.81[c])<br>Session length: 1.25-1.5h<br>Additional home practice: yes (daily; no practice log)	
Cohen [75] (2004) United States	n = 30<br>M_{Age} = 51<br>Hodgkin/non-Hodgkin Lymphoma<br>Stage: I-IV<br>During or after treatment (chemotherapy)	**BFI**	Content: Tibetan yoga, meditation, imagery, mild physical effort<br>(not reported: variety between sessions)<br>Setting: individually<br>Duration: 7 weeks<br>Session number: 7 (M = 4.6[c])<br>Session length: 1h<br>Additional home practice: yes (daily; no practice log)	Wait-List Control
Cramer [76] (2015) Germany	n = 40[d]<br>M_{Age} = 49.2<br>Breast Cancer<br>Stage: I-III<br>After treatment (surgery, and/or radiotherapy, and/or chemotherapy)	**FACIT-F**	Content: Hatha yoga, breathing techniques, meditation, moderate physical effort, variety between sessions<br>Setting: group-based<br>Duration: 12 weeks<br>Session number: 12 (M = 9.7)<br>Session length: 1.5h<br>Additional home practice: yes (daily; practice log)	Wait-List Control
Cramer [77] (2016) Germany	n = 54[d]<br>M_{Age} = 68.3<br>Colorectal Cancer<br>Stage: I-III<br>After/during treatment (after surgery, some during/after chemotherapy or after radiotherapy)	**FACIT-F**	Content: Hatha yoga, breathing techniques, meditation, moderate physical effort, variety between sessions<br>Setting: group-based<br>Duration: 10 weeks<br>Session number: 10 (M = 5.3)<br>Session length: 1.5h<br>Additional home practice: yes (daily; practice log)	Wait-List Control
Danhauer [78] (2009) United States	n = 27<br>M_{Age} = 55.8<br>Breast Cancer<br>Stage: DCIS, I-IV<br>After/during treatment (after surgery, some during	**FACT-F**	Content: Restorative yoga, breathing awareness (no techniques specified), meditation, mild physical effort, no variety between sessions<br>Setting: group-based<br>Duration: 10 weeks<br>Session number: 10 (M = 5.8)	Wait-List Control

	chemotherapy and/or radiotherapy)		Session length: 1.25h<br>Additional home practice: no	
Dhruva [79] (2012) United States	n = 16[e]<br>M_{Age} = 54.2<br>Mixed Entities (50% Breast Cancer)<br>Stage: not reported<br>During treatment (adjuvant or neoadjuvant chemotherapy)	**R-PFS**	Content: Pranayama breathing, breathing techniques, no physical effort, no variety between sessions<br>Setting: individually/group-based<br>Duration: two consecutive cycles of chemotherapy<br>Session number: 3[c]<br>Session length: 1h<br>Additional home practice: yes (twice a day; practice log)	Wait-List Control (received same intervention as EG in 2nd cycle of chemotherapy)
Jong [80] (2018) Netherlands	n = 68<br>M_{Age} = 51<br>Breast Cancer<br>Stage: I-III<br>During treatment (during neo-/adjuvant chemotherapy, some before/after radiotherapy)	**MFI (general**, physical fatigue, mental fatigue, reduced activity, reduced motivation)<br>FQL (frustrating, exhausting, pleasant, frightening)	Content: Dru yoga, breathing techniques, imagery, moderate physical effort, no variety between sessions<br>Setting: group-based<br>Duration: 12 weeks<br>Session number: 12 (M = 8[c])<br>Session length: 1.25h<br>Additional home practice: yes (daily; practice log)	Wait-List Control
Kiecolt-Glaser[d] [81] (2014) United States	n = 186<br>M_{Age} = 51.6<br>Breast Cancer<br>Stage: 0-IIIa<br>After treatment (surgery, some additional chemotherapy and/or radiotherapy)	**MFSI-SF**	Content: Hatha yoga, breathing techniques, moderate physical effort, variety between sessions<br>Setting: group-based<br>Duration: 12 weeks<br>Session number: 24 (M = 18.1)<br>Session length: 1.5h<br>Additional home practice: yes (no specific time recommended; practice log)	Wait-List Control
Lin [82] (2019) United States	n = 328<br>M_{Age} = 54.3<br>Mixed Entities (77% Breast Cancer)<br>Stage: 0-IV	**MFSI (total**, general, physical, emotional, mental, vigor)	Content: Hatha and Restorative yoga, breathing techniques, meditation, imagery, moderate physical effort, no variety between sessions<br>Setting: group-based<br><br>Duration: 4 weeks	Standard Care

	After treatment (surgery and/or chemotherapy and/or radiotherapy and/or hormone therapy)		Session number: 8-12 (*M* = 6.5)<br>Session length: 1.25h<br>Additional home practice: yes (once per week; no practice log)	
Littman [83] (2012) United States	*n* = 57<br>M_{Age} = 59.4<br>Breast Cancer<br>Stage: 0-III<br>After treatment (not reported which)	**FACIT**	Content: Hatha yoga (Viniyoga), breathing techniques, meditation, moderate physical effort, no variety between sessions<br>Setting: group-based<br>Duration: 6 months<br>Session number: *M* = 19.6 (2-3 facility-based classes a week encouraged)<br>Session length: 1.25h<br>Additional home practice: yes (remainder until reaching 5 weekly practices; reminder)	Wait-List Control
Loudon [84] (2014) Australia	*n* = 23<br>M_{Age} = 57.8<br>Breast Cancer<br>Stage: DCIS, I-III<br>After treatment (surgery, partly plus chemotherapy and/or radiotherapy)	**VAS (Fatigue severity**, fatique limit activity)	Content: based on Satyananda yoga tradition, breathing techniques, meditation, imagery, moderate physical effort, no variety between sessions<br>Setting: group-based<br>Duration: 8 weeks<br>Session number: 8 (*M* = 7.76[c])<br>Session length: 1.5h<br>Additional home practice: yes (daily, practice log)	Wait-List Control
Moadel [85] (2007) United States	*n* = 106[f]<br>M_{Age} = 54.8<br>Breast Cancer<br>Stage: I-IV<br>After/during treatment (surgery and/or chemotherapy and/or radiotherapy and/or hormone therapy)	**FACIT-F**	Content: Hatha yoga, breathing techniques, meditation, mild physical effort (not reported: variety between sessions)<br>Setting: group-based<br>Duration: 12 weeks<br>Session number: 12 (*M* = 7)<br>Session length: 1.5h<br>Additional home practice: yes (daily, no practice log)	Wait-List Control
Pasyar [86] (2019) Iran	*n* = 27<br>M_{Age} = 51.7<br>Breast Cancer	**EORTC QOL-C30**	Content: no specific yoga style, breathing techniques, mild physical effort, variety between sessions	Standard Care

	Stage: not reported<br>After treatment (surgery, partly plus chemotherapy and/or radiotherapy)		Setting: individually[c]<br>Duration: 8 weeks<br>Session number: 16<br>Session length: 0.75h<br>Additional home practice: yes (one session per week; practice log)	
Prakash [87]<br>(2020)<br>India	*n* = 83<br>M_{Age}: not reported<br>Breast Cancer<br>Stage: not reported<br>During adjuvant chemotherapy	**EORTC QOL-C30**	Content: no specific yoga style (neck and shoulder exercises), breathing techniques, mild physical effort, no variety between sessions<br>Setting: individually[c]<br>Duration: 18 weeks<br>Session number: 6<br>Session length: *M* = 1.16h<br>Additional home practice: yes (twice daily; no practice log)	Standard Care
Pruthi [88]<br>(2012)<br>United States	*n* = 28<br>M_{Age} = 56.5<br>Breast Cancer<br>Stage: not reported (non-metastatic)<br>During/after treatment (after surgery, some during adjuvant chemotherapy and/or radiotherapy and/or hormone therapy)	**BFI**	Content: gentle Hatha yoga, breathing awareness (no techniques specified), mild physical effort, no variety between sessions<br>Setting: individually and group-based<br>Duration: 12 weeks<br>Session number: 11 (*M* = 5.33[c])<br>Session length: 1h<br>Additional home practice: yes (at least once per week; no practice log)	Wait-List Control
Sohl [89]<br>(2016)<br>United States	*n* = 11<br>M_{Age} = 61<br>Colorectal Cancer<br>Stage: 0-IV<br>During treatment (chemotherapy)	**FACT-F**	Content: yoga skills training (seated), breathing techniques, meditation, mild physical effort, no variety between sessions<br>Setting: individually<br>Duration: 8 weeks<br>Session number: 3 (*M* = 2.91[c])<br>Session length: 0.25h<br>Additional home practice: yes (4 times per week; no practice log)	Attention Control (empathic attention and recorded education)

Sohl [90]<br>(2022)<br>United States	*n* = 34<br>M_{Age} = 58.5<br>Gastrointestinal Cancer<br>Stage: II–IV<br>During treatment<br>(chemotherapy)	**PROMIS-Cancer**	Content: yoga skills training (seated), breathing techniques, meditation, mild physical effort, no variety between sessions<br>Setting: individually<br>Duration: 8 weeks<br>Session number: 4 (*M* = 3.1)<br>Session length: 0.5h<br>Additional home practice: yes (daily; practice log)	Attention Control (empathic attention)
Taso [91]<br>(2014)<br>Taiwan	*n* = 60<br>M_{Age} = 49.3<br>Breast Cancer<br>Stage: I-III<br>During treatment<br>(chemotherapy)	**BFI**	Content: Yoga based on Anusara, breathing awareness (no techniques specified), meditation, moderate physical effort, no variety between sessions<br>Setting: group-based[c]<br>Duration: 8 weeks<br>Session number: 16 (*M* =15.09[c])<br>Session length: 1h<br>Additional home practice: yes (no time specified; no practice log)	Standard Care
Taylor [92]<br>(2018)<br>United States	*n* = 20<br>M_{Age} = 53.8<br>Breast Cancer<br>Stage: I-III<br>After treatment (different adjuvant treatments)	**BFI**	Content: restorative yoga, breathing awareness (no techniques specified), meditation, mild physical effort, no variety between sessions<br>Setting: group-based<br>Duration: 8 weeks<br>Session number: 8 (*M* = 4.88[c])<br>Session length: 1.25h<br>Additional home practice: no	Wait-List Control
Vadiraja [93]<br>(2009)<br>India	*n* = 75<br>M_{Age} = not reported (Range: 30-70)<br>Breast Cancer<br>Stage: I-III<br>During treatment<br>(radiotherapy, after surgery and/or chemotherapy)	**EORTC QLQ-C30**	Content: no specific yoga style, breathing techniques, meditation, imagery, mild physical effort, no variety between sessions<br>Setting: individually<br>Duration: 6 weeks<br>Session number: at least 18 (*M* = 21.31[c])<br>Session length: 1h<br>Additional home practice: yes (daily; no practice log)	Standard Care

Zetzl [94]<br>(2021)<br>Germany	n = 136[d, e]<br>M_{Age} = 60.4<br>Mixed Entities (49.1% Breast Cancer)<br>Stage: not reported (curative & palliative)<br>After/during treatment (chemotherapy and/or radiotherapy and/or hormone therapy and/or antibody therapy)	**EORTC QLQ-C30**	Content: no specific yoga style, breathing awareness (no techniques specified), meditation, moderate physical effort, no variety between sessions<br>Setting: group-based[c]<br>Duration: 8 weeks<br>Session number: 8 (M = 6.1[c])<br>Session length: 1h<br>Additional home practice: no	Wait-List Control
Zhi [95]<br>(2021)<br>United States	n = 35<br>M_{Age} = 61.7<br>Breast (92.7%), Ovarian, or Uterine Cancer<br>Stage: I-III<br>After treatment (chemotherapy)	**BFI**	Content: no specific yoga style, breathing techniques, moderate physical effort, no variety between sessions<br>Setting: group-based<br>Duration: 8 weeks<br>Session number: 16<br>Session length: 1h<br>Additional home practice: yes (5 times per week; no practice log)	Wait-List Control

Notes: EG = experimental group; MAge: Mean age of all participants at baseline; n refers to the total number of participants analyzed for fatigue assessment at post intervention; instruments marked in bold were used for analyzing total fatigue scores.
a Instruments:.
BFI: Brief Fatigue Inventory.
EORTC QLQ-C30: European Organization for Research and Treatment of Cancer Core Quality of Life Questionnaire - Fatigue Scale
FACIT: Functional Assessment of Chronic Illness Therapy
FACIT-F: Functional Assessment of Chronic Illness Therapy - Fatigue
FACT-B: Functional Assessment of Cancer Therapy - Breast Cancer
FACT-F: Functional Assessment of Cancer Therapy - Fatigue
FSI: Fatigue Symptom Inventory
FQL: Fatigue Quality List
MFI: Multidimensional Fatigue Inventory
MFSI: Multidimensional Fatigue Scale Inventory
MFSI-SF: Multidimensional Fatigue Syndrome Inventory - Short Form.
PROMIS-Cancer: Patient-Reported Outcomes Measurement Information System - Cancer
R-PFS: Revised Piper Fatigue Scale
VAS: Visual Analogue Scale
[b] Content:

breathing techniques: use of at least one technique aimed at conscious breathing.
imagery: any exercise aimed at creating visual representations.
meditation: any mindfulness practice aimed at concentration on the present moment.
physical effort: no physical effort = no movement exercise; mild = only sitting or lying positions; moderate = yoga exercises were performed in different positions (or assessment based on intervention description).
variety between sessions: yoga exercises changed from session to session.
session number: indicated is the planned session number and, if indicated, average participation in sessions by participants in parentheses.
practice at home: indicated in parentheses is the frequency of practice and the type of recommendation (voluntarily, asked, encouraged, expected).
c The information was not explicitly given in the manuscript but could be inferred.
d Missing post-intervention values were imputed.
e Participants had to report some degree of fatigue as inclusion criteria.
f n=22 outliers were omitted.

Table S2. 2. Characteristics of included psychosocial interventions.

Study (Author + Year + Country)	Description of study population	Instrument(s) used to measure fatigue[a]	Description of intervention[b]	Description of control
Andersen [96] (2004) United States	*n* = 196 M_{Age} = 50.8 Breast Cancer Stage: II-III During treatment (adjuvant chemotherapy)	**POMS-F**	Content: work on cognitions, work on behavior, social resources, relaxation Setting: group-based, face to face and via telephone (in absence) Duration: 4 months Session number: 18 (*M* = 17) (+ 8 follow up sessions) Session length: 1.5h	Standard care
Armes [97] (2007) United Kingdom	*n* = 36[c] M_{Age} = 59.1 Mixed Entities (33% Colon Cancer patients) Stage: I-IV During treatment (chemotherapy)	**VAS (global fatigue)**	Content[d]: CRF education, work on cognitions, work on behavior Setting: individually, face to face Duration: 9-12 weeks Session number: 3 (*M* = 2.6[e]) Session length: 1h	Standard care
Arving [98] (2007) EG 1 Sweden	*n* = 89 M_{Age} = 55 Breast Cancer Stage: 0-IV During treatment (adjuvant chemotherapy, radiotherapy, and/or hormone therapy)	**EORTC QLQ-C30**	Content: work on cognitions, work on behavior, social resources, relaxation, homework (practice strategies) (all conducted by psychologists) Setting: individually, face to face and via telephone Duration: *M* = 210 days Session number: 0-23 (*M* = 4.5) ession length: 0.75-1h	Standard Care
Arving [98] (2007) EG 2 Sweden	*n* = 80 M_{Age} = 55 Breast Cancer Stage: 0-IV During treatment (adjuvant chemotherapy,	**EORTC QLQ-C30**	Content: work on cognitions, work on behavior, social resources, relaxation, homework (practice strategies) (all conducted by nurses) Setting: individually, face to face and via telephone Duration: *M* = 172 days Session number: 0-16 (*M* = 3.8)	Standard Care

	radiotherapy, and/or hormone therapy)		Session length: 0.75-1h	
Bourmaud [99] (2017) France	n = 147[c] M_{Age} = 55.9 Mixed Entities (58.5% Breast Cancer) Stage: all During treatment (chemotherapy and/or radiotherapy)	**FACT-F** PFS (behavioural/severity, affective meaning, sensory, cognitive/mood) EORTC QLQ-C30 VAS (fatigue severity)	Content[d]: CRF education, work on behavior Setting: group-based, face to face Duration: 6 weeks Session number: 5 (M = 3.77) Session length: 2h	Wait-List Control
Cohen & Fried [100] (2007) Israel	n = 75 M_{Age} = 54.4 Breast Cancer Stage: I-II During treatment (adjuvant chemotherapy and/or radiotherapy)	**FSI (severity,** interference, frequency**)**	Content: work on cognitions, work on behavior, homework (practice strategies) Setting: group-based, face to face Duration: 9 weeks Session number: 9 Session length: 1.5h	Standard Care
Courtier [101] (2022) United Kingdom	n = 31[c] M_{Age} = 58.3 Breast Cancer Stage: 0–IIIA During treatment (radiotherapy, partly after chemotherapy)	**FACIT-F**	Content[d]: CRF education, work on cognitions, work on behavior, work on emotions, social resources, homework (activity and sleep diary) Setting: individually, face to face Duration: 3 weeks Session number: 3 Session length: 1h	Standard Care
Dolbeault [102] (2009) France	n = 168 M_{Age} = 53.1 Breast Cancer Stage: "Early stage" After treatment (radiotherapy alone or combined with chemotherapy)	**POMS-F** EORTC QLQ-C30	Content: work on cognitions, work on behavior, social resources, relaxation Setting: group-based, face to face Duration: 8 weeks Session number: 8 Session length: 2h	Wait-List Control

Fukui [103] (2000) Japan	$n = 50$<br>$M_{Age} = 53.5$<br>Breast Cancer<br>Stage: I-III<br>After treatment (surgery, some after chemotherapy)	**POMS-F**	Content: work on cognitions, work on behavior, social resources, relaxation, homework (practice relaxation)<br>Setting: group-based, face to face<br>Duration: 6 weeks<br>Session number: 6<br>Session length: 1.5h	Wait-List Control
Gaston-Johansson [104] (2000) United States	$n = 110$<br>M_{Age} = not reported<br>Breast Cancer<br>Stage: II-IV<br>During treatment (neoadjuvant chemotherapy; after autologous bone marrow or peripheral blood stem cell transplantation)	**VAS (degree of fatigue)**	Content: work on cognitions, relaxation, homework (practice relaxation)<br>Setting: individually[e], face to face<br>Duration: 9 days<br>Session number: 4<br>Session length: 1h (initial session) – 0.25h (Follow-up sessions)	Standard Care
Godino [105] (2006) Spain	$n = 20$<br>$M_{Age} = 60.3$<br>Colon or Gastric Cancer<br>Stage: not reported<br>During treatment (chemotherapy)	**FACT-F**	Content: CRF education, work on behavior, homework (diary)<br>Setting: individually, face to face<br>Duration: 6 months[e]<br>Session number: 3<br>Session length: not reported (0.5h assumed)	Standard Care
Goedendorp [106] (2010) Netherlands	$n = 148$[f]<br>$M_{Age} = 56.4$<br>Mixed Entities (47.3% Breast Cancer patients)<br>Stage: not reported (curative treatment)<br>During treatment (surgery, chemotherapy, radiotherapy, and/or hormone therapy)	**CIS-fat**	Content[d]: CRF education, work on cognitions, work on behavior, social resources<br>Setting: individually, face to face and via telephone<br>Duration: 6 months<br>Session number: up to 10 ($M = 6.2$)<br>Session length: 1h	Standard Care
Grégoire [107] (2020) Belgium	$n = 95$[c, f]<br>$M_{Age} = 53.9$<br>Mixed Entities	**MFI (general fatigue**, physical fatigue, mental	Content: work on cognitions, work on behavior, relaxation, hypnosis, homework (self-care tasks and diary)	Wait-List Control

	(78.9% Breast Cancer patients)<br>Stage: not reported (non-metastatic)<br>After treatment (surgery, chemotherapy, radiotherapy, and/or hormone therapy)	fatigue, reduced motivation, reduced activity)	Setting: group-based, face to face<br>Duration: 8 weeks<br>Session number: 8 (M = 6.5)<br>Session length: 2h	
Kim [108]<br>(2018)<br>South Korea	n = 55<br>M_{Age} = 48<br>Breast Cancer<br>Stage: I-III<br>During treatment (adjuvant chemotherapy)	**EORTC QLQ-C30**	Content: work on cognitions, work on behavior, work on emotions, social resources<br>Setting: individually, face-to-face and via telephone<br>Duration: 7 weeks<br>Session number: 7<br>Session length: 0.5-1h	Standard Care
Montgomery [109]<br>(2014)<br>United States	n = 181<br>M_{Age} = 55.8<br>Breast Cancer<br>Stage: 0-III<br>During treatment (radiotherapy, some with chemotherapy before and/or adjuvant hormone therapy)	**FACIT-F**<br>VAS (fatigue, muscle weakness)	Content[d]: work on cognitions, work on behavior, relaxation, hypnosis, homework (workbook)<br>Setting: individually, face to face<br>Duration: length of radiotherapy<br>Session number: individual (M = 12.29)<br>Session length: 15 min (regular sessions); 30 min (initial and final session)	Attention Control (conversation-based support)
O'Brien [110]<br>(2014)<br>Australia	n = 26<br>M_{Age} = 61.3<br>Mixed Entities (60% Breast Cancer patients)<br>Stage: not reported<br>During treatment (chemotherapy or radiotherapy)	**MFI** (**general**, physical, mental fatigue, reduced activity, reduced motivation)	Content[d]: CRF education, work on behavior<br>Setting: individually, face to face and via telephone<br>Duration: 4 weeks<br>Session number: 3 (2 via telephone)<br>Session length: 1h (face to face session; telephone sessions not specified)	Standard Care
Peng [111]<br>(2019)<br>China	n = 155<br>M_{Age} = 60.4<br>Lung Cancer	**EORTC QLQ-C30**	Content: work on cognitions, work on behavior, social resources<br>Setting: group-based, face to face	Standard Care

	Stage: III-IV<br>During treatment<br>(radiotherapy)		Duration: 8 weeks<br>Session number: 8<br>Session length: 1.5h	
Purcell [112]<br>(2011)<br>Australia	*n* = 45<br>M_{Age} = 58.8<br>Mixed Entities<br>(35.8% Breast Cancer patients)<br>Stage: not reported<br>Before & after treatment<br>(radiotherapy)	**MFI** (**general**, physical, mental fatigue, reduced activity, reduced motivation)	Content[d]: CRF education, work on behavior<br>Setting: group-based, face to face and via telephone<br>Duration: length of radiotherapy + 5-6 weeks<br>Session number: 4 (2 via telephone)<br>Session length: 1h (face to face sessions; telephone sessions not specified)	Standard Care
Ream [113]<br>(2006)<br>United Kingdom	*n* = 86<br>M_{Age} = 56.5<br>Mixed Entities<br>(22% esophageal cancer)<br>Stage: not reported<br>(curative and palliative)<br>During treatment<br>(chemotherapy)	**VAS (mean fatigue score**, extent of fatigue, distress caused by fatigue, disruption to work/chores by fatigue, disruption to pastimes/hobbies by fatigue)	Content[d]: CRF education, work on behavior, homework (diary)<br>Setting: individually, face to face<br>Duration: three chemotherapy cycles (9 weeks assumed)<br>Session number: 3<br>Session length: not reported (1h assumed)	Standard Care
Reif [114]<br>(2013)<br>Germany	*n* = 234[c]<br>M_{Age} = 57.7<br>Mixed Entities<br>(58,6% breast cancer)<br>Stage: not reported<br>After treatment<br>(all kind of therapies)	**FAQ**<br>EORTC QLQ-C30	Content[d]: CRF education, work on cognitions, work on behavior, work on emotions, homework (diary, different exercises)<br>Setting: group-based, face to face<br>Duration: 6 weeks<br>Session number: 6<br>Session length: 1.5h	Wait-List Control
Sadeghi [115]<br>(2016)<br>Iran	*n* = 135[f]<br>M_{Age} = 55.7<br>Breast Cancer<br>Stage: l-lllA<br>During treatment<br>(chemotherapy)	**CFS (total**, physical, cognitive, affective)	Content[d]: work on behavior, homework (monitoring, activity list)<br>Setting: group-based, face to face<br>Duration: 5 weeks<br>Session number: 5<br>Session length: 1.5h	Wait-List Control

Salvetti [116] (2021) Brazil	*n* = 108[e] M_{Age} = 55.2 Mixed Entities (50% colorectal cancer) Stage: not reported (37,5% with metastasis) During treatment (chemotherapy or radiotherapy)	**EORTC-QLQ-C30**	Content: CRF education, work on cognitions, work on behavior, relaxation, homework (practice relaxation) Setting: individually, face to face Duration: 6 weeks Session number: 6 Session length: *M* = 0.75h	Standard Care
Schjolberg [117] (2014) Norway	*n* = 92[c] M_{Age} = 55.3 Breast Cancer Stage: I-II After treatment (chemotherapy, radiotherapy; some during their final radiation, and/or during hormone therapy)	**FQ (total**, physical, mental, chronic) LFS (fatigue, energy)	Content[d]: CRF education, work on behavior, homework (diary) Setting: group-based, face to face Duration: 3 weeks Session number: 3 Session length: 2h	Standard Care
Sheikhzadeh [118] (2021) Iran	*n* = 39 M_{Age} = 47.8 Mixed Entities (67% breast cancer) Stage: not reported (41.4% with metastasis) Treatment status: not reported (chemotherapy, hormone therapy, target therapy, and/or radiotherapy)	**CFS**	Content: work on cognitions, work on behavior, relaxation Setting: group-based, face to face Duration: 8 weeks Session number: 8 Session length: 1.5h	Wait-List Control
Tu [119] (2021) China	*n* = 100 M_{Age} = 56.9[e] Lung Cancer Stage: II-IV During treatment (chemotherapy)	**CFS-Total Fatigue**	Content: work on cognitions, work on emotions, social resources Setting: individually, face to face Duration: 4 weeks Session number: 8 Session length: 0.5-0.67h	Standard Care

Van der Meulen [120] (2014) Netherlands	n = 179[f] M_{Age} = 60.4 Head and Neck Cancer Stage: I-IV After treatment (surgery and/or radiotherapy and/or chemotherapy)	**EORTC QLQ-C30**	Content: work on cognitions, social resources, work on emotions/relaxation (if indicated) Setting: individually, face to face Duration: 1 year Session number: maximum 6 (M = 4.00) Session length: 0.75-1h	Standard Care
Vargas [121] (2014) United States	n = 194 M_{Age} = 50.3 (at diagnosis) Breast Cancer Stage: 0-lll During treatment (adjuvant radiotherapy and/or chemotherapy and/or hormone therapy)	**9-point Likert scale (fatigue intensity**, fatigue-related daytime interference)	Content[d]: work on cognitions, work on behavior, social resources, relaxation, homework (workbook) Setting: group-based, face to face Duration: 10 weeks Session number: 10 (M = 6.78) Session length: 2h	Attention Control (one-day 6 h psycho-education session)
Xian [122] (2021) China	n = 119[f] M_{Age} = 60.7 Colorectal Cancer Stage: II-IV During treatment (chemotherapy, partly surgery)	**CFS-C (Total**, Physical, Affective, Cognitive)	Content[d]: work on behavior, homework Setting: individually, face to face Duration: 24 weeks Session number: 6 Session length: 0.5h	Standard Care
Yates [123] (2005) Australia	n = 104 M_{Age} = 49.4 Breast Cancer Stage: I-II During treatment (adjuvant chemotherapy)	**FACT** 11-Point Numeric Rating Scales (worst, best, average fatigue) R-PFS (distress, interference, severity)	Content[d]: CRF education, work on cognitions, work on behavior, social resources Setting: individually, face to face and via telephone Duration: 2 weeks Session number: 3 (2 via telephone) Session length: 10 min (telephone) - 20 min (face to face)	Attention Control (general cancer education sessions)
Yuen [124] (2006) United States	n = 12[c] M_{Age} = 55.4	**PFS (total**, affective meaning, work	Content[d]: work on behavior, homework (activity log)	Standard Care

	Mixed Entities (not reported which)<br>Stage: not reported<br>After treatment (radiotherapy)	on behavior/severity, cognitive/mood, sensory)	Setting: individually, face to face and via telephone<br>Duration: 4 weeks<br>Session number: 4 (3 via telephone)<br>Session length: 1-2h (face to face); 15-30 minutes (telephone)	
Zhang [125] (2020) China	*n* = 145<br>M_{Age} = 60.3<br>Colorectal Cancer<br>Stage: I-IV<br>After treatment (surgery and/or chemotherapy, and/or radiotherapy)	**EORTC QLQ-C30**	Content: work on cognitions, work on behavior, work on emotions, relaxation, homework (practice assignments)<br>Setting: individually, face to face<br>Duration: 3 months<br>Session number: 6<br>Session length: 1h	Standard Care

Notes: EG = experimental group; MAge: Mean age of all participants at baseline; Instruments marked in bold were used for analyzing total fatigue scores; *n* refers to the total number of participants analyzed for fatigue assessment at post intervention.
a Instruments:.
CFS-C: Cancer-Related Fatigue Scale
CIS-fat: Checklist Individual Strength (CIS) – Fatigue Subscale.
EORTC QLQ-C30: European Organization for Research and treatment of Cancer Core Quality of Life Questionnaire – Fatigue Scale
FACT-F: Functional Assessment of Cancer Therapy – Fatigue
FAQ: Fatigue Assessment Questionnaire
FQ: Norwegian Fatigue Questionnaire (Norwegian version of The Fatigue Formula).
FSI: Fatigue Symptom Inventory
LFS: Lee Fatigue Scale
MFA: Multidimensional Fatigue Assessment Questionnaire
MFI: Multidimensional Fatigue Inventory
PFS: Piper Fatigue Scale
POMS-F: Profile of Moods State - Fatigue Symptom Subscale.
R-PFS: Revised Piper Fatigue Scale
VAS: Visual Analog Scale
b Content:.
CRF education: psychoeducative information on cancer-related fatigue.
work on cognitions: use of at least one therapeutic technique to establish more functional thinking patterns.
work on behavior: use of at least one therapeutic technique that rehearses more functional behavior.
work on emotions: advice and/or exercises to better cope with emotions.
social resources: activation of social resources through strengthening social support and/or communication training.
relaxation: therapeutic technique designed to put participants in a more relaxed state of mind, including mental imagery.
hypnosis: technique aimed at an altered state of consciousness, characterized by high susceptibility to external influence.

session number: indicated is the planned session number and, if indicated, average participation in sessions by participants in parentheses.
c Participants had to report some degree of cancer-related fatigue as inclusion criteria.
d Intervention focus was on cancer-related fatigue.
e The information was not explicitly given in the manuscript but could be inferred.
f Missing post-intervention values were imputed.

Table S2. 3. Characteristics of included mindfulness-based interventions.

Study (Author + Year + Country)	Description of study population	Instrument(s) used to measure fatigue[a]	Description of intervention[b]	Description of control
Bower [126] (2015) United States	*n* = 65<br>M_{Age} = 46.8<br>Breast Cancer<br>Stage: 0-III<br>After treatment (chemotherapy, radiotherapy, Herceptin intake)	**FSI** (scale not reported)	Content: meditation (experiential practice, gentle movement exercises)<br>*(Mindful Awareness Practices program)*<br>Setting: group-based<br>Duration: 6 weeks<br>Session number: 6 (*M*=5.24)<br>Session length: 2h<br>Additional home practice: yes (daily; practice log)	Wait-List Control
Bower [127] (2021) United States	*n* = 149<br>M_{Age} = 45.4<br>Breast Cancer<br>Stage: 0-III<br>After treatment (surgery, radiotherapy, chemotherapy, and/or hormone therapy)	**FSI** (scale not reported)	Content: meditation (experiential practice, gentle movement exercises)<br>*(Mindful Awareness Practices program)*<br>Setting: group-based<br>Duration: 6 weeks (+4 and 8 weeks after the 6-week program)<br>Session number: 6 (*M*= 4.5) + 2 1h booster sessions<br>Session length: 2h<br>Additional home practice: yes (daily; practice log)	Wait-List Control
Gok Metin [128] (2019) Turkey	*n* = 61[c]<br>M_{Age} = 50.4<br>Breast Cancer<br>Stage: I-III<br>During treatment (chemotherapy)	**BFI** (**severity**, interference)	Content: meditation (breathing)<br>*(Mindfulness meditation)*<br>Setting: individually<br>Duration: 12 weeks<br>Session number: 1 (+ daily text messages or phone calls)<br>Session length: 0.67h<br>Additional home practice: yes (daily; reminder)	Attention Control (one education session)
Hoffman [129] (2012) United Kingdom	*n* = 214<br>M_{Age} = 49.6<br>Breast Cancer	**POMS-F**	Content: meditation (body scan, sitting), yoga exercises	Wait-List Control

	Stage: 0-III<br>After treatment<br>(surgery, chemotherapy and/or radiotherapy)		*(Mindfulness-based Stress Reduction)*<br>Setting: group-based<br>Duration: 8 weeks<br>Session number: 9 (*M* = 6.26, excluding 6h-session)<br>Session length: 2-2.25h plus one 6h-session<br>Additional home practice: yes (daily; practice log)	
Johns [130]<br>(2015)<br>United States	*n* = 35[d]<br>M_{Age} = 57.3<br>Mixed Entities<br>(85.7% Breast Cancer)<br>Stage: I-IV<br>After treatment<br>(chemotherapy, and/or radiotherapy)	**FSI** (**severity**, interference, frequency)<br>SF-36	Content[e]: CRF education, meditation (body scan, compassion, sitting, walking), yoga exercises<br>*(Mindfulness-based Stress Reduction – CRF)*<br>Setting: group-based<br>Duration: 7 weeks<br>Session number: 7 (*M* = 6.16)<br>Session length: 2h<br>Additional home practice: yes (daily; practice log)	Wait-List Control
Lengacher [131]<br>(2012)<br>United States	*n* = 82<br>M_{Age} = not reported<br>Breast Cancer<br>Stage: 0-III<br>After treatment<br>(adjuvant chemotherapy and/or radiotherapy)	**MDASI**	Content: meditation (body scan, sitting, walking), yoga exercises<br>*(Mindfulness-based Stress Reduction (for Breast Cancer))*<br>Setting: group-based<br>Duration: 6 weeks<br>Session number: 6<br>Session length: 2h<br>Additional home practice: yes (frequency not specified; no practice log)	Wait-List Control
Lengacher [132]<br>(2016)<br>United States	*n* = 299<br>M_{Age} = 57.1<br>Breast Cancer<br>Stage: 0-III<br>After treatment<br>(surgery, majority adjuvant chemotherapy and/or radiotherapy)	**FSI** (**severity**, interference)	Content: meditation (body scan, sitting, walking), yoga exercises<br>*(Mindfulness-based Stress Reduction (for Breast Cancer))*<br>Setting: group-based<br>Duration: 6 weeks<br>Session number: 6<br>Session length: 2h<br>Frequency: weekly	Wait-List Control

			Additional home practice: yes (frequency not specified; no practice log)	
Liu [133] (2019) China	*n* = 102<br>M_{Age} = 42.8<br>Thyroid Cancer<br>Stage: I-IV<br>During treatment (thyroid hormone withdrawal, before radioactive iodine therapy)	**EORTC-QLQ-C30**	Content: meditation (body scan, breathing, nonjudgmental awareness, sitting, walking), yoga exercises<br>(*Mindfulness-based Stress Reduction*)<br>Setting: group-based<br>Duration: 8 weeks<br>Session number: 8 (*M* = 6.76)<br>Session length: 2h[f]<br>Additional home practice: yes (frequency not specified; no practice log)	Standard Care
Ng [134] (2021) Malaysia	*n* = 78[d]<br>M_{Age} = 54.6<br>Haematological Cancers (58.9% Lymphoma)<br>Stage: not reported<br>Treatment status: not reported	**FACIT-F**<br>ESAS-F	Content[e]: meditation (breathing)<br>*(Mindful Breathing Session*)<br>Setting: individually<br>Duration: 1 day<br>Session number: 1<br>Session length: 0.75h[f]<br>Additional home practice: no	Standard Care
Park [135] (2020) Japan	*n* = 74[c]<br>M_{Age} = 53.7<br>Breast Cancer<br>Stage: 0-III<br>Treatment status: not reported	**BFI**	Content: meditation (breathing, body scan, compassion, eating, walking), work on cognitions, yoga exercises<br>*(Mindfulness-based cognitive therapy)*<br>Setting: group-based<br>Duration: 8 weeks<br>Session number: 8<br>Session length: 2h<br>Additional home practice: yes (daily; practice log)	Wait-List Control
Rahmani [136] (2014) Iran	*n* = 24<br>M_{Age} = 43.7<br>Breast Cancer<br>Stage: I-III<br>During treatment (chemotherapy)	**EORTC-QLQ-C30**	Content: meditation (breathing, body scan, eating, sitting), yoga exercises<br>(*Mindfulness-based Stress Reduction*)<br>Setting: group-based<br>Duration: 8 weeks<br>Session number: 8<br>Session length: 2h	Standard Care

			Additional home practice: no	
Sheikhzadeh [118] (2021) Iran	*n* = 39<br>M_{Age} = 47.8<br>Breast Cancer (67.2%), Lymphoma, Colon Cancer<br>Stage: not reported (41.4% with metastasis)<br>Treatment status: not reported (chemotherapy, hormone therapy, target therapy, and/or radiotherapy)	**CFS**	Content: CRF education, meditation (body scan, breathing, eating), work on cognitions<br>(*Mindfulness Based Cognitive Therapy*)<br>Setting: group-based<br>Duration: 8 weeks<br>Session number: 8<br>Session length: 1.5h<br>Additional home practice: no	Wait-List Control
Van der Gucht [137] (2020) Belgium	*n* = 25<br>M_{Age} = 45.5<br>Breast Cancer<br>Stage: "early stage"<br>After treatment (chemotherapy, partly radiotherapy and/or hormone therapy)	**CIS - fatigue severity**	Content: meditation (body scan, breathing, insight, walking), work on cognitions, yoga exercises<br>(*Mindfulness-Based Intervention*)<br>Setting: group-based<br>Duration: 8 weeks<br>Session number: 4<br>Session length: 3h<br>Additional home practice: yes (daily; reminder)	Wait-List Control
Van der Lee [138] (2012) Netherlands	*n* = 83[d]<br>M_{Age} = 51.3<br>Mixed Entities (63% Breast Cancer)<br>Stage: not reported<br>After treatment (surgery, chemotherapy, radiotherapy, and/or hormone therapy)	**CIS-fat**	Content[e]: CRF education, meditation (body scan, breathing, compassion, eating), work on cognitions<br>(*Mindfulness-based Cognitive Therapy*)<br>Setting: group-based<br>Duration: 9 weeks<br>Session number: 9 (+1 follow-up session) (*M*=8)<br>Session length: 2.5h and one 6h-session<br>Additional home practice: yes (6 days a week; practice log)	Wait-List Control
Witek Janusek [139] (2019) United States	*n* = 137<br>M_{Age} = 55.1<br>Breast Cancer<br>Stage: 0-III	**MFSI-SF**	Content: meditation (breathing, sitting, walking), yoga exercises<br>(*Mindfulness-based Stress Reduction*)<br>Setting: group-based	Attention Control (educational series)

After/during treatment (some with adjuvant chemotherapy, radiotherapy, and/or hormone therapy)	Duration: 8 weeks<br>Session number: 9<br>Session length: 2.5h and one 6h-session<br>Additional home practice: yes (no frequency specified; no practice log)

Notes: MAge: Mean age of all participants at baseline; *n* refers to the total number of participants analyzed for fatigue assessment at post intervention; instruments marked in bold were used for analyzing total fatigue scores.
a Instruments:.
BFI: Brief Fatigue Inventory
CIS-fat: Checklist Individual Strength – Fatigue Severity Subscale.
EORTC QLQ-C30: European Organization for Research and Treatment of Cancer Core Quality of Life Questionnaire – Fatigue Scale.
ESAS: Edmonton Symptom Assessment System – Fatigue Subscale
FACT-F: Functional Assessment of Cancer Therapy – Fatigue
FSI: Fatigue Symptom Inventory
MDASI: M.D. Anderson Symptom Inventory
MFSI-SF: Multidimensional Fatigue Scale Inventory - Short Form
POMS: Profile of Moods State - Fatigue Symptom Subscale
SF-12: Medical Outcomes Study Short-Form Health Survey
SF-36: Medical Outcomes Study Short-Form Health Survey.
VAS: Visual Analog Scale.
b Content:.
CRF education: psychoeducative information on cancer-related fatigue.
meditation: any mindfulness practice aimed at concentration on the present moment.
body scan: mindfulness exercise in which attention is focused on different areas of the body.
work on cognitions: explicit working on cognitions/thoughts according to Mindfulness-based Cognitive Therapy.
yoga exercises: any yoga type.
session number: planned session number and, if indicated, average participation in sessions by participants in parentheses.
c Missing post-intervention values were imputed.
d Participants had to report some degree of fatigue as inclusion criteria.
e Intervention focus was on cancer-related fatigue.
f The information was not explicitly given in the manuscript but could be inferred.

References

71. Banasik, J.; Williams, H.; Haberman, M.; Blank, S.E.; Bendel, R. Effect of iyengar yoga practice on fatigue and diurnal salivary cortisol concentration in breast cancer survivors. *J Am Acad Nurse Pract* **2011**, *23*, 135-142.
72. Chandwani, K.D.; Perkins, G.; Nagendra, H.R.; Raghuram, *n*.V.; Spelman, A.; Nagarathna, R.; Johnson, K.; Fortier, A.; Arun, B.; Wei, Q., *et al.* Randomized, controlled trial of yoga in women with breast cancer undergoing radiotherapy. *J Clin Oncol* **2014**, *32*, 1058-1065.
73. Chandwani, K.D.; Thornton, B.; Perkins, G.H.; Arun, B.; Raghuram, N.V.; Nagendra, H.R.; Wei, Q.; Cohen, L. Yoga improves quality of life and benefit finding in women undergoing radiotherapy for breast cancer. *J Soc Integr Oncol* **2010**, *8*, 43-55.
74. Chaoul, A.; Milbury, K.; Spelman, A.; Basen-Engquist, K.; Hall, M.H.; Wei, Q.; Shih, Y.-C.T.; Arun, B.; Valero, V.; Perkins, G.H., *et al.* Randomized trial of tibetan yoga in patients with breast cancer undergoing chemotherapy. *Cancer* **2018**, *124*, 36-45.
75. Cohen, L.; Warneke, C.; Fouladi, R.T.; Rodriguez, M.A.; Chaoul-Reich, A. Psychological adjustment and sleep quality in a randomized trial of the effects of a tibetan yoga intervention in patients with lymphoma. *Cancer* **2004**, *100*, 2253-2260.
76. Cramer, H.; Pokhrel, B.; Fester, C.; Meier, B.; Gass, F.; Lauche, R.; Eggleston, B.; Walz, M.; Michalsen, A.; Kunz, R., *et al.* A randomized controlled bicenter trial of yoga for patients with colorectal cancer. *Psychooncology* **2016**, *25*, 412-420.
77. Cramer, H.; Rabsilber, S.; Lauche, R.; Kümmel, S.; Dobos, G. Yoga and meditation for menopausal symptoms in breast cancer survivors-a randomized controlled trial. *Cancer* **2015**, *121*, 2175-2184.
78. Danhauer, S.C.; Mihalko, S.L.; Russell, G.B.; Campbell, C.R.; Felder, L.; Daley, K.; Levine, E.A. Restorative yoga for women with breast cancer: Findings from a randomized pilot study. *Psychooncology* **2009**, *18*, 360-368.
79. Dhruva, A.; Miaskowski, C.; Abrams, D.; Acree, M.; Cooper, B.; Goodman, S.; Hecht, F.M. Yoga breathing for cancer chemotherapy-associated symptoms and quality of life: Results of a pilot randomized controlled trial. *J Altern Complement Med* **2012**, *18*, 473-479.
80. Jong, M.C.; Boers, I.; Schouten van der Velden, A.P.; Meij, S.V.; Göker, E.; Timmer-Bonte, A.; van Wietmarschen, H.A. A randomized study of yoga for fatigue and quality of life in women with breast cancer undergoing (neo) adjuvant chemotherapy. *J Altern Complement Med* **2018**, *24*, 942-953.
81. Kiecolt-Glaser, J.K.; Bennett, J.M.; Andridge, R.; Peng, J.; Shapiro, C.L.; Malarkey, W.B.; Emery, C.F.; Layman, R.; Mrozek, E.E.; Glaser, R. Yoga's impact on inflammation, mood, and fatigue in breast cancer survivors: A randomized controlled trial. *J Clin Oncol* **2014**, *32*, 1040-1049.
82. Lin, P.J.; Kleckner, I.R.; Loh, K.P.; Inglis, J.E.; Peppone, L.J.; Janelsins, M.C.; Kamen, C.S.; Heckler, C.E.; Culakova, E.; Pigeon, W.R., *et al.* Influence of yoga on cancer-related fatigue and on mediational relationships between changes in sleep and cancer-related fatigue: A nationwide, multicenter randomized controlled trial of yoga in cancer survivors. *Integr Cancer Ther* **2019**, *18*, 1-11.
83. Littman, A.J.; Bertram, L.C.; Ceballos, R.; Ulrich, C.M.; Ramaprasad, J.; McGregor, B.; McTiernan, A. Randomized controlled pilot trial of yoga in overweight and obese breast cancer survivors: Effects on quality of life and anthropometric measures. *Support Care Cancer* **2012**, *20*, 267-277.
84. Loudon, A.; Barnett, T.; Piller, N.; Immink, M.A.; Williams, A.D. Yoga management of breast cancer-related lymphoedema: A randomised controlled pilot-trial. *BMC Complement Altern Med* **2014**, *14*, 214.
85. Moadel, A.B.; Shah, C.; Wylie-Rosett, J.; Harris, M.S.; Patel, S.R.; Hall, C.B.; Sparano, J.A. Randomized controlled trial of yoga among a multiethnic sample of breast cancer patients: Effects on quality of life. *J Clin Oncol* **2007**, *25*, 4387-4395.
86. Pasyar, N.; Barshan Tashnizi, N.; Mansouri, P.; Tahmasebi, S. Effect of yoga exercise on the quality of life and upper extremity volume among women with breast cancer related lymphedema: A pilot study. *Eur J Oncol Nurs* **2019**, *42*, 103-109.
87. Prakash, K.; Saini, S.K.; Pugazhendi, S. Effectiveness of yoga on quality of life of breast cancer patients undergoing chemotherapy: A randomized clinical controlled study. *Indian J Palliat Care* **2020**, *26*, 323-331.
88. Pruthi, S.; Stan, D.L.; Jenkins, S.M.; Huebner, M.; Borg, B.A.; Thomley, B.S.; Cutshall, S.M.; Singh, R.; Kohli, S.; Boughey, J.C., *et al.* A randomized controlled pilot study assessing feasibility and impact of yoga practice on quality of life, mood, and perceived stress in women with newly diagnosed breast cancer. *Glob Adv Health Med* **2012**, *1*, 30-35.

89. Sohl, S.J.; Danhauer, S.C.; Birdee, G.S.; Nicklas, B.J.; Yacoub, G.; Aklilu, M.; Avis, N.E. A brief yoga intervention implemented during chemotherapy: A randomized controlled pilot study. *Complement Ther Med* **2016**, *25*, 139-142.
90. Sohl, S.J.; Tooze, J.A.; Johnson, E.N.; Ridner, S.H.; Rothman, R.L.; Lima, C.R.; Ansley, K.C.; Wheeler, A.; Nicklas, B.; Avis, N.E., *et al.* A randomized controlled pilot study of yoga skills training versus an attention control delivered during chemotherapy administration. *J Pain Symptom Manag* **2022**, *63*, 23-32.
91. Taso, C.J.; Lin, H.S.; Lin, W.L.; Chen, S.M.; Huang, W.T.; Chen, S.W. The effect of yoga exercise on improving depression, anxiety, and fatigue in women with breast cancer: A randomized controlled trial. *J Nurs Res* **2014**, *22*, 155-164.
92. Taylor, T.R.; Barrow, J.; Makambi, K.; Sheppard, V.; Wallington, S.F.; Martin, C.; Greene, D.; Yeruva, S.L.H.; Horton, S. A restorative yoga intervention for african-american breast cancer survivors: A pilot study. *J Racial Ethn Health Disparities* **2018**, *5*, 62-72.
93. Vadiraja, S.H.; Rao, M.R.; Nagendra, R.H.; Nagarathna, R.; Rekha, M.; Vanitha, N.; Gopinath, S.K.; Srinath, B.; Vishweshwara, M.; Madhavi, Y., *et al.* Effects of yoga on symptom management in breast cancer patients: A randomized controlled trial. *Int J Yoga Therap* **2009**, *2*, 73-79.
94. Zetzl, T.; Renner, A.; Pittig, A.; Jentschke, E.; Roch, C.; van Oorschot, B. Yoga effectively reduces fatigue and symptoms of depression in patients with different types of cancer. *Support Care Cancer* **2021**, *29*, 2973-2982.
95. Zhi, W.I.; Baser, R.E.; Zhi, L.M.; Talukder, D.; Li, Q.S.; Paul, T.; Patterson, C.; Piulson, L.; Seluzicki, C.; Galantino, M.L., *et al.* Yoga for cancer survivors with chemotherapy-induced peripheral neuropathy: Health-related quality of life outcomes. *Cancer Med* **2021**, *10*, 5456-5465.
96. Andersen, B.L.; Farrar, W.B.; Golden-Kreutz, D.M.; Glaser, R.; Emery, C.F.; Crespin, T.R.; Shapiro, C.L.; Carson, W.E., 3rd. Psychological, behavioral, and immune changes after a psychological intervention: A clinical trial. *J Clin Oncol* **2004**, *22*, 3570-3580.
97. Armes, J.; Chalder, T.; Addington-Hall, J.; Richardson, A.; Hotopf, M. A randomized controlled trial to evaluate the effectiveness of a brief, behaviorally oriented intervention for cancer-related fatigue. *Cancer* **2007**, *110*, 1385-1395.
98. Arving, C.; Sjödén, P.O.; Bergh, J.; Hellbom, M.; Johansson, B.; Glimelius, B.; Brandberg, Y. Individual psychosocial support for breast cancer patients: A randomized study of nurse versus psychologist interventions and standard care. *Cancer nursing* **2007**, *30*, E10-19.
99. Bourmaud, A.; Anota, A.; Moncharmont, C.; Tinquaut, F.; Oriol, M.; Trillet-Lenoir, V.; Bajard, A.; Parnalland, S.; Rotonda, C.; Bonnetain, F., *et al.* Cancer-related fatigue management: Evaluation of a patient education program with a large-scale randomised controlled trial, the peps fatigue study. *Br J Cancer* **2017**, *116*, 849-858.
100. Cohen, M.; Fried, G. Comparing relaxation training and cognitive-behavioral group therapy for women with breast cancer. *Research on Social Work Practice.* **2007**, *17*, 313-323.
101. Courtier, N.; Armes, J.; Smith, A.; Radley, L.; Hopkinson, J.B. Targeted self-management limits fatigue for women undergoing radiotherapy for early breast cancer: Results from the active randomised feasibility trial. *Support Care Cancer* **2022**, *30*, 389-400.
102. Dolbeault, S.; Cayrou, S.; Brédart, A.; Viala, A.L.; Desclaux, B.; Saltel, P.; Gauvain-Piquard, A.; Hardy, P.; Dickes, P. The effectiveness of a psycho-educational group after early-stage breast cancer treatment: Results of a randomized french study. *Psychooncology* **2009**, *18*, 647-656.
103. Fukui, S.; Kugaya, A.; Okamura, H.; Kamiya, M.; Koike, M.; Nakanishi, T.; Imoto, S.; Kanagawa, K.; Uchitomi, Y. A psychosocial group intervention for japanese women with primary breast carcinoma. *Cancer* **2000**, *89*, 1026-1036.
104. Gaston-Johansson, F.; Fall-Dickson, J.M.; Nanda, J.; Ohly, K.V.; Stillman, S.; Krumm, S.; Kennedy, M.J. The effectiveness of the comprehensive coping strategy program on clinical outcomes in breast cancer autologous bone marrow transplantation. *Cancer nursing* **2000**, *23*, 277-285.
105. Godino, C.; Jodar, L.; Durán, A.; Martínez, I.; Schiaffino, A. Nursing education as an intervention to decrease fatigue perception in oncology patients. *Eur J Oncol Nurs* **2006**, *10*, 150-155.
106. Goedendorp, M.M.; Peters, M.E.; Gielissen, M.F.; Witjes, J.A.; Leer, J.W.; Verhagen, C.A.; Bleijenberg, G. Is increasing physical activity necessary to diminish fatigue during cancer treatment? Comparing cognitive behavior therapy and a brief nursing intervention with usual care in a multicenter randomized controlled trial. *Oncologist* **2010**, *15*, 1122-1132.
107. Grégoire, C.; Faymonville, M.E.; Vanhaudenhuyse, A.; Charland-Verville, V.; Jerusalem, G.; Willems, S.; Bragard, I. Effects of an intervention combining self-care and self-hypnosis on fatigue and associated symptoms in post-treatment cancer patients: A randomized-controlled trial. *Psychooncology* **2020**, *29*, 1165-1173.

108. Kim, Y.H.; Choi, K.S.; Han, K.; Kim, H.W. A psychological intervention programme for patients with breast cancer under chemotherapy and at a high risk of depression: A randomised clinical trial. *J Clin Nurs* **2018**, *27*, 572-581.
109. Montgomery, G.H.; David, D.; Kangas, M.; Green, S.; Sucala, M.; Bovbjerg, D.H.; Hallquist, M.N.; Schnur, J.B. Randomized controlled trial of a cognitive-behavioral therapy plus hypnosis intervention to control fatigue in patients undergoing radiotherapy for breast cancer. *J Clin Oncol* **2014**, *32*, 557-563.
110. O'Brien, L.; Loughnan, A.; Purcell, A.; Haines, T. Education for cancer-related fatigue: Could talking about it make people more likely to report it? *Support Care Cancer* **2014**, *22*, 209-215.
111. Peng, W.; Zhang, H.; Li, Z. Responses of lung cancer survivors undergoing gamma knife surgery to supportive group psychotherapy. *Medicine* **2019**, *98*, e14693.
112. Purcell, A.; Fleming, J.; Burmeister, B.; Bennett, S.; Haines, T. Is education an effective management strategy for reducing cancer-related fatigue? *Support Care Cancer* **2011**, *19*, 1429-1439.
113. Ream, E.; Richardson, A.; Alexander-Dann, C. Supportive intervention for fatigue in patients undergoing chemotherapy: A randomized controlled trial. *J Pain Symptom Manag* **2006**, *31*, 148-161.
114. Reif, K.; de Vries, U.; Petermann, F.; Görres, S. A patient education program is effective in reducing cancer-related fatigue: A multi-centre randomised two-group waiting-list controlled intervention trial. *Eur J Oncol Nurs* **2013**, *17*, 204-213.
115. Sadeghi, E.; Gozali, N.; Moghaddam Tabrizi, F. Effects of energy conservation strategies on cancer related fatigue and health promotion lifestyle in breast cancersurvivors: A randomized control trial. *Asian Pac J Cancer Prev* **2016**, *17*, 4783-4790.
116. Salvetti, M.G.; Donato, S.C.T.; Machado, C.S.P.; de Almeida, N.G.; Santos, D.V.D.; Kurita, G.P. Psychoeducational nursing intervention for symptom management in cancer patients: A randomized clinical trial. *Asia Pac J Oncol Nurs* **2021**, *8*, 156-163.
117. Schjolberg, T.K.; Dodd, M.; Henriksen, N.; Asplund, K.; Cvancarova Småstuen, M.; Rustoen, T. Effects of an educational intervention for managing fatigue in women with early stage breast cancer. *Eur J Oncol Nurs* **2014**, *18*, 286-294.
118. Sheikhzadeh, M.; Zanjani, Z.; Baari, A. Efficacy of mindfulness-based cognitive therapy and cognitive behavioral therapy for anxiety, depression, and fatigue in cancer patients: A randomized clinical trial. *Iran J Psychiatry* **2021**, *16*, 271-280.
119. Tu, M.; Wang, F.; Shen, S.; Wang, H.; Feng, J. Influences of psychological intervention on negative emotion, cancer-related fatigue and level of hope in lung cancer chemotherapy patients based on the perma framework. *Iran J Public Health* **2021**, *50*, 728-736.
120. van der Meulen, I.C.; May, A.M.; de Leeuw, J.R.; Koole, R.; Oosterom, M.; Hordijk, G.J.; Ros, W.J. Long-term effect of a nurse-led psychosocial intervention on health-related quality of life in patients with head and neck cancer: A randomised controlled trial. *Br J Cancer* **2014**, *110*, 593-601.
121. Vargas, S.; Antoni, M.H.; Carver, C.S.; Lechner, S.C.; Wohlgemuth, W.; Llabre, M.; Blomberg, B.B.; Glück, S.; DerHagopian, R.P. Sleep quality and fatigue after a stress management intervention for women with early-stage breast cancer in southern florida. *Int J Behav Med* **2014**, *21*, 971-981.
122. Xian, X.; Zhu, C.; Chen, Y.; Huang, B.; Xiang, W. Effect of solution-focused therapy on cancer-related fatigue in patients with colorectal cancer undergoing chemotherapy: A randomized controlled trial. *Cancer nursing* **2021**.
123. Yates, P.; Aranda, S.; Hargraves, M.; Mirolo, B.; Clavarino, A.; McLachlan, S.; Skerman, H. Randomized controlled trial of an educational intervention for managing fatigue in women receiving adjuvant chemotherapy for early-stage breast cancer. *J Clin Oncol* **2005**, *23*, 6027-6036.
124. Yuen, H.K.; Mitcham, M.; Morgan, L. Managing post-therapy fatigue for cancer survivors using energy conservation training. *J Allied Health* **2006**, *35*, 121e-139e.
125. Zhang, X.; Liu, J.; Zhu, H.; Zhang, X.; Jiang, Y.; Zhang, J. Effect of psychological intervention on quality of life and psychological outcomes of colorectal cancer patients. *Psychiatry* **2020**, *83*, 58-69.
126. Bower, J.E.; Crosswell, A.D.; Stanton, A.L.; Crespi, C.M.; Winston, D.; Arevalo, J.; Ma, J.; Cole, S.W.; Ganz, P.A. Mindfulness meditation for younger breast cancer survivors: A randomized controlled trial. *Cancer* **2015**, *121*, 1231-1240.
127. Bower, J.E.; Partridge, A.H.; Wolff, A.C.; Thorner, E.D.; Irwin, M.R.; Joffe, H.; Petersen, L.; Crespi, C.M.; Ganz, P.A. Targeting depressive symptoms in younger breast cancer survivors: The pathways to wellness randomized controlled trial of mindfulness meditation and survivorship education. *J Clin Oncol* **2021**, *39*, 3473-3484.

128. Gok Metin, Z.; Karadas, C.; Izgu, N.; Ozdemir, L.; Demirci, U. Effects of progressive muscle relaxation and mindfulness meditation on fatigue, coping styles, and quality of life in early breast cancer patients: An assessor blinded, three-arm, randomized controlled trial. *Eur J Oncol Nurs* **2019**, *42*, 116-125.
129. Hoffman, C.J.; Ersser, S.J.; Hopkinson, J.B.; Nicholls, P.G.; Harrington, J.E.; Thomas, P.W. Effectiveness of mindfulness-based stress reduction in mood, breast- and endocrine-related quality of life, and well-being in stage 0 to iii breast cancer: A randomized, controlled trial. *J Clin Oncol* **2012**, *30*, 1335-1342.
130. Johns, S.A.; Brown, L.F.; Beck-Coon, K.; Monahan, P.O.; Tong, Y.; Kroenke, K. Randomized controlled pilot study of mindfulness-based stress reduction for persistently fatigued cancer survivors. *Psychooncology* **2015**, *24*, 885-893.
131. Lengacher, C.A.; Reich, R.R.; Post-White, J.; Moscoso, M.; Shelton, M.M.; Barta, M.; Le, N.; Budhrani, P. Mindfulness based stress reduction in post-treatment breast cancer patients: An examination of symptoms and symptom clusters. *J Behav Med* **2012**, *35*, 86-94.
132. Lengacher, C.A.; Reich, R.R.; Paterson, C.L.; Ramesar, S.; Park, J.Y.; Alinat, C.; Johnson-Mallard, V.; Moscoso, M.; Budhrani-Shani, P.; Miladinovic, B., *et al.* Examination of broad symptom improvement resulting from mindfulness-based stress reduction in breast cancer survivors: A randomized controlled trial. *J Clin Oncol* **2016**, *34*, 2827-2834.
133. Liu, T.; Zhang, W.; Xiao, S.; Xu, L.; Wen, Q.; Bai, L.; Ma, Q.; Ji, B. Mindfulness-based stress reduction in patients with differentiated thyroid cancer receiving radioactive iodine therapy: A randomized controlled trial. *Cancer Manag Res* **2019**, *11*, 467-474.
134. Ng, D.L.; Gan, G.G.; Anuar, N.A.; Tung, Y.Z.; Lai, N.Z.; Tan, Y.W.; Said, S.N.M.; Madihie, A.; Chai, C.S.; Tan, S.B. The effect of a single session of 30-min mindful breathing in reducing fatigue among patients with haematological cancer - a randomised controlled trial. *BMC Palliat Care* **2021**, *20*, 160.
135. Park, S.; Sato, Y.; Takita, Y.; Tamura, N.; Ninomiya, A.; Kosugi, T.; Sado, M.; Nakagawa, A.; Takahashi, M.; Hayashida, T., *et al.* Mindfulness-based cognitive therapy for psychological distress, fear of cancer recurrence, fatigue, spiritual well-being, and quality of life in patients with breast cancer-a randomized controlled trial. *J Pain Symptom Manag* **2020**, *60*, 381-389.
136. Rahmani, S.; Talepasand, S.; Ghanbary-Motlagh, A. Comparison of effectiveness of the metacognition treatment and the mindfulness-based stress reduction treatment on global and specific life quality of women with breast cancer. *Iran J Cancer Prev* **2014**, *7*, 184-196.
137. Van der Gucht, K.; Ahmadoun, S.; Melis, M.; de Cloe, E.; Sleurs, C.; Radwan, A.; Blommaert, J.; Takano, K.; Vandenbulcke, M.; Wildiers, H., *et al.* Effects of a mindfulness-based intervention on cancer-related cognitive impairment: Results of a randomized controlled functional magnetic resonance imaging pilot study. *Cancer* **2020**, *126*, 4246-4255.
138. van der Lee, M.L.; Garssen, B. Mindfulness-based cognitive therapy reduces chronic cancer-related fatigue: A treatment study. *Psychooncology* **2012**, *21*, 264-272.
139. Witek Janusek, L.; Tell, D.; Mathews, H.L. Mindfulness based stress reduction provides psychological benefit and restores immune function of women newly diagnosed with breast cancer: A randomized trial with active control. *Brain Behav Immun* **2019**, *80*, 358-373.
