# Supplementary material for: Meta-Analysis of Randomized Controlled Trials on Yoga, Psychosocial, and Mindfulness-Based Interventions for Cancer-Related Fatigue: What Intervention Characteristics Are Related to Higher Efficacy?
Source: Cancers (Basel). 2022 Apr 15;14(8):2016. doi: 10.3390/cancers14082016 (PMC9032769; doi:10.3390/cancers14082016)
Supplement: Supplementary file 1 [file cancers-14-02016-s001.zip › Supplementary Tables S3_Model Selection_Proof.pdf]

Table S3. 1. Meta-regression models with the best model fit for explaining standard mean differences in yoga interventions.

	Model 1	Model 2	Model 3	Model 4	Model 5
	ß (SE)	ß (SE)	ß (SE)	ß (SE)	ß (SE)
**Intercept**	−0.62 (0.21)**	−0.99 (0.32)**	−0.21 (0.199)	−0.42 (0.17)*	−0.30 (0.16)
**Breathing technique[a]**	0.34 (0.23)	0.53 (0.26)	-	-	-
**Group setting[a]**	-	0.35 (0.24)	-	0.11 (0.23)	-
**Mental practice[a]**	-	-	−0.19 (0.23)	-	-
**Physical effort[a]**	-	-	-	-	−0.10 (0.22)
**Total intervention Time[b]**	−0.03 (0.09)	−0.11 (0.11)	−0.03 (0.10)	−0.04 (0.11)	0.00 (0.11)
**AICc**	39.43	39.91	41.11	41.66	41.71
**Weight**	0.15	0.12	0.06	0.05	0.05

a 1=yes; 0=no.
b Variable was z-standardized and fixed in all models.
Note: ß: regression coefficient; SE: standard error; AICc: Akaike's information criterion corrected; **p*<.05; ***p*<.01.
Five best models based on their AICc are shown (apart from intercept-only model); negative regression coefficients indicate a higher intervention effect on cancer-related fatigue.
Variety between sessions was not identified as relevant variable in the top−5 models.

Table S3. 2. Meta-regression models with the best model fit for explaining standard mean differences in psychosocial interventions.

	Model 1	Model 2	Model 3	Model 4	Model 5
	ß (SE)	ß (SE)	ß (SE)	ß (SE)	ß (SE)
**Intercept**	−0.09 (0.21)	−0.17 (0.20)	−0.12 (0.20)	−0.00 (0.24)	−0.01 (0.28)
**Group setting[a]**	−0.66 (0.20)**	−0.59 (0.20)**	−0.63 (0.22)**	−0.67 (0.20)**	−0.66 (0.20)**
**Relaxation[a]**	0.73 (0.16)***	0.79 (0.16)***	0.56 (0.16)**	0.71 (0.17)***	0.72 (0.16)***
**Work on cognitions[a]**	−0.72 (0.17)***	−0.85 (0.18)***	−0.74 (0.19)***	−0.78 (0.19)***	−0.74 (0.18)***
**CRF education[a]**	0.37 (0.15)*	0.40 (0.15)*	-	0.43 (0.17)*	0.39 (0.16)*
**Social resources**	-	0.22 (0.14)	-	-	-
**Focus on fatigue[a]**	-	-	-	−0.12 (0.17)	-
**Work on behavior[a]**	-	-	-	-	−0.10 (0.21)
**Total Intervention Time[b]**	0.23 (0.10)*	0.19 (0.10)	0.19 (0.11)	0.22 (0.10)*	0.24 (0.10)*
**AICc**	33.15	33.98	35.99	36.25	36.55
**Weight**	0.18	0.13	0.06	0.05	0.04

a 1=yes; 0=no.
b Variable was z-standardized and fixed in all models.
Note: ß: regression coefficient; SE: standard error; AICc: Akaike's information criterion corrected; CRF=cancer-related fatigue;

*$p<.05$; **$p<.01$;***$p<.001$.

Five best models based on their AICc are shown; negative regression coefficients indicate a higher intervention effect on cancer-related fatigue. Work on emotions was not identified as relevant variable in the top–5 models.

Table S3. 3. Meta-regression models with the best model fit for explaining standard mean differences in mindfulness-based interventions.

	Model 1	Model 2	Model 3	Model 4
	ß (SE)	ß (SE)	ß (SE)	ß (SE)
**Intercept**	−0.60 (0.16)**	−0.30 (0.63)	−0.53 (0.27)	−0.64 (0.17)
**CRF education**[a]	−0.39 (0.48)	-	-	-
**Group setting**[a]	-	−0.36 (0.67)	-	-
**Yoga exercises**[a]	-	-	−0.14 (0.34)	-
**Work on cognitions**[a]	-	-	-	0.05 (0.35)
**Total intervention time**[b, c]	0.06 (0.15)	0.10 (0.22)	0.04 (0.16)	0.01 (0.15)
**CRF as inclusion criterion**[a, c]	−0.33 (0.45)	−0.65 (0.41)	−0.61 (0.39)	−0.56 (0.38)
**AICc**	36.34	36.91	37.06	37.30
**Weight**	0.10	0.08	0.07	0.06

a 1=yes; 0=no.

b Variable was z-standardized.

c Variable was fixed in all models.

Note: ß: regression coefficient; SE: standard error; AICc: Akaike's information criterion corrected; CRF=cancer-related fatigue; **$p<.01$.

All four models within six units of the AICc of the best model are shown (apart from intercept-only model; negative regression coefficients indicate a higher intervention effect on cancer-related fatigue.

.
